# Transcriptome-wide selection and validation of a solid set of reference genes for gene expression studies in the cephalopod mollusk *Octopus vulgaris*

**DOI:** 10.3389/fnmol.2023.1091305

**Published:** 2023-05-17

**Authors:** Pamela Imperadore, Stefano Cagnin, Vittoria Allegretti, Caterina Millino, Francesca Raffini, Graziano Fiorito, Giovanna Ponte

**Affiliations:** ^1^Department of Biology and Evolution of Marine Organisms, Stazione Zoologica Anton Dohrn, Napoli, Italy; ^2^Department of Biology, University of Padova, Padova, Italy; ^3^CIR-Myo Myology Center, University of Padova, Padova, Italy

**Keywords:** *Octopus vulgaris*, reference genes, qRT-PCR, cephalopods, nervous system, molecular fingerprint

## Abstract

*Octopus vulgaris* is a cephalopod mollusk and an active marine predator that has been at the center of a number of studies focused on the understanding of neural and biological plasticity. Studies on the machinery involved in e.g., learning and memory, regeneration, and neuromodulation are required to shed light on the conserved and/or unique mechanisms that these animals have evolved. Analysis of gene expression is one of the most essential means to expand our understanding of biological machinery, and the selection of an appropriate set of reference genes is the prerequisite for the quantitative real-time polymerase chain reaction (qRT-PCR). Here we selected 77 candidate reference genes (RGs) from a pool of stable and relatively high-expressed transcripts identified from the full-length transcriptome of *O. vulgaris*, and we evaluated their expression stabilities in different tissues through *geNorm*, *NormFinder*, *Bestkeeper*, Delta-CT method, and *RefFinder*. Although various algorithms provided different assemblages of the most stable reference genes for the different kinds of tissues tested here, a comprehensive ranking revealed RGs specific to the nervous system (*Ov-RNF7* and *Ov-RIOK2*) and *Ov-EIF2A* and *Ov-CUL1* across all considered tissues. Furthermore, we validated RGs by assessing the expression profiles of nine target genes (*Ov-Naa15*, *Ov-Ltv1*, *Ov-CG9286*, *Ov-EIF3M*, *Ov-NOB1*, *Ov-CSDE1*, *Ov-Abi2*, *Ov-Homer2*, and *Ov-Snx20*) in different areas of the octopus nervous system (gastric ganglion, as control). Our study allowed us to identify the most extensive set of stable reference genes currently available for the nervous system and appendages of adult *O. vulgaris*.

## Introduction

Cephalopods and particularly the common octopus, *Octopus vulgaris,* are among the key invertebrate organisms recognized for their complex neural organization. The common octopus is an iconic species among cephalopods, at the center of a long tradition of research in diverse aspects of its biology and physiology (e.g., O’Brien et al., 2019).

The taxon belongs to Lophotrochozoa (i.e., a protostome animal), and thus it is very distant from vertebrates. Nevertheless, octopuses are known for possessing the largest nervous system among invertebrates in terms of the number of cells and body-to-brain size (Young, 1963; Packard and Albergoni, 1970; Giuditta et al., 1971; Packard, 1972), as well as their intricate neural network and manifold cellular complexity (Young, 1932; Shigeno and Ragsdale, 2015; Chung et al., 2022; Styfhals et al., 2022; Chung et al., 2023), with remarkable functional analogies to vertebrates (Shigeno et al., 2015, 2018). *Octopus vulgaris* has also served as an organism of study for the identification of the neural correlates of learning and memory and the search for a model of the brain (Young, 1964; Kandel, 1979; review in: Hochner et al., 2006; Borrelli and Fiorito, 2008; Marini et al., 2017). Nowadays, these mollusks continue to inspire the search for the biological and neural machinery underlying plasticity and cognition (Edelman and Seth, 2009; Albertin and Simakov, 2020; Ponte et al., 2022).

Over the last decade, a significant increase in the efforts of the scientific community has facilitated the release of a large set of genomic data (see Supplementary Info) for various cephalopod species, including the transcriptomes of *O. vulgaris* (Zhang et al., 2012; Petrosino, 2015; Petrosino et al., 2015; Liscovitch-Brauer et al., 2017; Petrosino et al., 2022; Prado-Álvarez et al., 2022; Styfhals et al., 2022) and reference genomes for more than 10 species (Supplementary Table 1). Although these resources still do not comprehensively represent the rich biological diversity of the approximately 800 living cephalopod species, their availability has greatly contributed to illuminating the biological and physiological complexity of these organisms and the ‘innovations’ they provided during their evolution (Albertin and Simakov, 2020; Albertin et al., 2022; Macchi et al., 2022; Schmidbaur et al., 2022).

These datasets, however, are not sufficient for the understanding of the molecular machinery implicated in neural plasticity (*sensu*: Cavallaro et al., 2002; Martinez et al., 2007; Asok et al., 2019). In addition, the current knowledge of the gene expression changes occurring in these animals during learning, memory, and behavioral plasticity is still poor. Only a few available studies are focused on some candidate molecules that are potentially involved in given functions. Thus, to the best of our knowledge, an investigation of the differential gene expression occurring in the brain of any cephalopod is still lacking. Here we contribute with a first step to fulfil this gap.

The accurate analysis of gene expression relies on the quantitative real-time polymerase chain reaction (qRT-PCR), one of the most utilized tools for assessing gene levels in different samples in experimental or biological conditions (Bustin, 2002; Huggett et al., 2005; Nolan et al., 2006). The technique offers numerous advantages (Bustin, 2002; Bustin et al., 2005). Nevertheless, its reliability and accuracy are based on the choice of reference genes (RGs) required for normalizing the expression levels of a given target gene. An ideal RG should have a moderate and stable expression level in different tissues, across biological phenomena, and under different experimental treatments (Huggett et al., 2005; Udvardi et al., 2008).

Most of the commonly used RGs for data normalization are the so-called housekeeping genes (e.g., elongation factor 1α, α-tubulin and β-tubulin, β-actin, and ubiquitin). Although widely employed in several species, in some instances they might lack the required stability when tested in different organisms and/or experimental contexts. In some circumstances, they may not match the requirements of an ideal candidate RG (Rubie et al., 2005; Hong et al., 2008; Eisenberg and Levanon, 2013).

In cephalopods, previous studies identified several candidate RGs (Supplementary Table 2) in a number of tissues (mainly brain masses), but to the best of our knowledge, they never encompassed testing of the peripheral ganglia or arms.

Our approach to build a list of potential stable reference genes in octopus was based on: (i) increasing the number of tissues to consider and (ii) exploring the available transcriptomes for *O. vulgaris* (Zhang et al., 2012; Petrosino, 2015; Petrosino et al., 2015, 2022). We selected genes that appeared stable and uniform in different tissues through *in silico* characterization of transcriptomes. Finally, we explored relative gene expression through qRT-PCR experiments by using a subset of target genes of the known expression *in silico*, thus validating our data and the use of the selected RGs in the brain and other ganglia. This approach allowed us to identify the most extensive set of stable reference genes currently available for adult *O. vulgaris* in the central and peripheral nervous system and in complex structures such as arms.

## Materials and methods

### *In silico* selection of candidate reference genes

**Figure 1:**
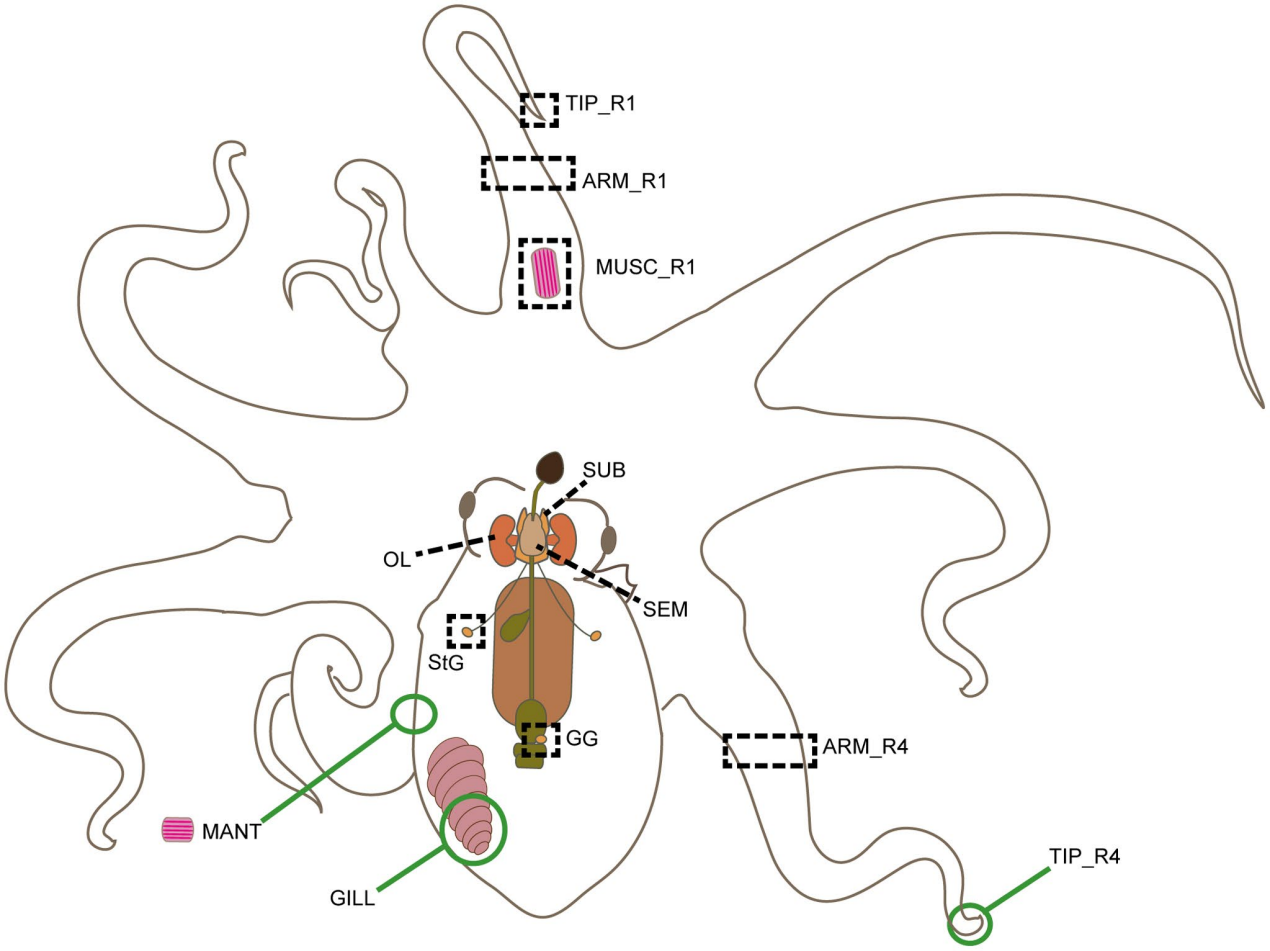
Schematic outline of *Octopus vulgaris* anatomy. Tissues sampled for transcriptomic analysis and biological validation are highlighted here. Black dotted rectangles and lines identify tissues included both in RNA-seq analysis and RT-qPCR experiments: supraoesophageal mass (SEM), suboesophageal mass (SUB) and left optic lobe (OL), gastric ganglion (GG), stellate ganglion (StG), R1 arm tip (Tip_R1), a piece of R1 and R4 arms (ARM_R1 and ARM_R4), and a piece of muscle from arm R1 (MUSC_R1). Green circles identify tissues only included in RT-qPCR experiments, i.e., a posterior portion of the left gill (GILL), a piece of muscles from the ventral side of the mantle without the skin (MANT), and R4 arm tip (Tip_R4).

Potential RGs were identified through *in silico* analysis of the RNA-seq available for *O. vulgaris* (for details, including assembly methods, see: Petrosino, 2015; Petrosino et al., 2015, 2022). The data included whole transcriptomes from nine tissues: the lobes of the adult octopus’ central nervous system (optic lobes, OL; supra-, SEM, and sub-oesophageal masses, SUB); the first anterior right arm (R1) with its distal extremity (Tip_R1), a proximal portion (ARM_R1), and muscle tissue (MUSC_R1; i.e., only muscle bundles, not the skin and arm nerve cord); the fourth posterior right arm (R4) with its proximal portion (ARM_R4); and two peripheral ganglia i.e. the left stellate and gastric ganglia (StG and GG, respectively; Figure 1).

As aforementioned, our rationale was to extend the biological diversity of the considered tissues. In addition, the anterior versus posterior arms were included on the basis of the scientific evidence of the potential variety of behavioral functions these may achieve (e.g., Mather, 1998; Huffard et al., 2005; Amodio et al., 2021).

Details on RNA isolation, quality and quantity assessment, and libraries construction are available in Petrosino (2015) and Petrosino et al. (2022) and not provided herein. Raw reads were analyzed by Trimmomatic (Bolger et al., 2014), which served for the filtering and trimming of low-quality bases. Normalization was performed, and the remaining reads were assembled in putative clustered transcripts to select unique sequences using Trinity (Grabherr et al., 2011). The raw reads were then mapped to the assembled transcriptome to measure the expression levels. Only annotated transcripts with a relative abundance greater than 1.5 counts per million (TPM) in all the biological replicates were considered. The annotation of these transcripts was finalized using the Annocript pipeline (Musacchia et al., 2015; Petrosino, 2015), thus counting 21,030 protein-coding sequences.

In order to identify the candidate RGs, we selected sequences based on their coefficient of variation (CV) of the relative abundance (TPM) of each transcript, i.e., the ratio of the standard deviation to the group mean of each transcript identified for four groups of tissues, as follows: (i) all the available tissues from adult individuals of *O. vulgaris* (**Adult**); (ii) the brain masses (**Brain**: SEM, SUB, and OL); (iii) The nervous tissues (**Nervous**: including tissues already listed in **Brain** group, plus StG and GG); (iv) tissues belonging to the arm (**Arm:** Tip_R1, ARM_R1, ARM R4, and MUSCLE_R1).

Genes were considered stable when their transcript’s CV was lower than 15%. For the **Adult** group, a cut-off of 20% CV was used to account for the higher tissue variability. Some genes were included in more than one group according to their CV values (Table 1 and Supplementary Figure 1).

**Table 1:** Genes identified in whole transcriptomes and validated in RT-qPCR experiments.

Transcript ID	Group	Gene name	Description	Accession number	CV%
c35016_g13_i1	Nervous system	*Ov-Gsk3b*	Glycogen synthase kinase-3 beta	MW800694	3.89
c34071_g2_i1	Nervous system	*Ov-mts*	Serine/threonine protein phosphatase PP2A	MW800693	4.27
c30725_g11_i1	Nervous System	*Ov-timm*	Mitochondrial import inner membrane translocase subunit Tim22	MW800652	4.40
c36083_g5_i1	Nervous System	*Ov-SUCLG2*	Succinate––CoA ligase [GDP-forming] subunit beta, mitochondrial	MW800659	4.52
c33604_g6_i1	Nervous System	*Ov-CHCHD7*	Coiled-coil-helix-coiled-coil-helix domain-containing protein 7	MW800655	4.63
c32222_g5_i1	Nervous system	*Ov-UBE2F*	NEDD8-conjugating enzyme UBE2F	MW800681	5.16
c34932_g8_i1	Nervous system	*Ov-MTX1*	Metaxin-1	MW800712	5.19
c31554_g1_i3	Nervous System	*Ov-gk5*	Putative glycerol kinase 5	MW800648	5.74
c35771_g14_i2	Nervous system	*Ov-Gnaq*	Guanine nucleotide-binding protein G(q) subunit alpha	MW800695	5.92
c35786_g9_i1	Nervous System	*Ov-Naa15*	N-alpha-acetyltransferase 15 NatA auxiliary subunit	MW800658	6.35
c30400_g11_i1	Nervous System	*Ov-wdr44*	WD repeat-containing protein 44	MW800651	6.97
c17784_g1_i1	Nervous System	*Ov-Klhdc*	Kelch domain-containing protein 4	MW800649	6.98
c35707_g2_i1	Nervous System	*Ov-PRMT5*	Protein arginine N-methyltransferase 5	MW800660	7.15
c32096_g14_i2	Nervous System	*Ov-Canx*	Calnexin	MW800654	7.33
c35499_g5_i1	Nervous System	*Ov-ube2c*	Ubiquitin-conjugating enzyme E2 C	MW800657	7.79
c33913_g6_i1	Nervous System	*Ov-PTPN12*	Tyrosine-protein phosphatase non-receptor type 12	MW800656	9.22
c31227_g1_i2	Nervous System	*Ov-tollip*	Toll-interacting protein	MW800653	9.24
c31322_g1_i1	Nervous System	*Ov-prrc1*	Protein PRRC1-A	MW800647	9.63
c28856_g1_i2	Nervous System	*Ov-CUL1*	Cullin-1	MW800650	9.91
c32222_g5_i1	ADULT	*Ov-UBE2F*	NEDD8-conjugating enzyme UBE2F	MW800681	8.81
c35707_g2_i1	ADULT	*Ov-PRMT5*	Protein arginine N-methyltransferase 5	MW800660	10.25
c25466_g1_i1	ADULT	*Ov-Ltv1*	Protein LTV1 homolog	MW800662	11.27
c35311_g1_i1	ADULT	*Ov-CPIJ005834*	Elongation factor G mitochondrial	MW800676	12.23
c35010_g2_i4	ADULT	*Ov-EIF2A*	Eukaryotic translation initiation factor 2A	MW800674	12.66
c29044_g1_i1	ADULT	*Ov-rpf1*	Ribosome production factor 1	MW800663	12.73
c31610_g1_i1	ADULT	*Ov-slc25a40*	Solute carrier family 25 member 40	MW800667	12.79
c33222_g7_i1	ADULT	*Ov-RIOK2*	Serine/threonine protein kinase RIO2	MW800670	12.85
c32170_g13_i2	ADULT	*Ov-Dap3*	28S ribosomal protein S29, mitochondrial	MW800668	12.87
c34313_g4_i1	ADULT	*Ov-Ppm1b*	Protein phosphatase 1B	MW800677	14.23
c34059_g14_i1	ADULT	*Ov-ATPAF2*	ATP synthase mitochondrial F1 complex assembly factor 2	MW800671	14.36
c30066_g9_i1	ADULT	*Ov-NOB1*	RNA-binding protein NOB1	MW800666	14.38
c32751_g1_i1	ADULT	*Ov-flr*	Actin-interacting protein 1	MW800669	15.29
c34776_g5_i1	ADULT	*Ov-usp10*	Ubiquitin carboxyl-terminal hydrolase 10	MW800673	15.39
c35032_g7_i2	ADULT	*Ov-Dnaja3*	DnaJ homolog subfamily A member 3, mitochondrial	MW800675	15.61
c34087_g16_i1	ADULT	*Ov-CSDE1*	Cold shock domain-containing protein E1	MW800672	15.63
c29524_g1_i1	ADULT	*Ov-EIF3M*	Eukaryotic translation initiation factor 3 subunit M	MW800665	15.71
c36175_g1_i1	ADULT	*Ov-BTBD17*	BTB/POZ domain-containing protein 17	MW800661	15.76
c29430_g1_i1	ADULT	*Ov-CG9286*	Protein BCCIP homolog	MW800664	16.21
c34939_g11_i1	ARM	*Ov-ESR16*	Ecdysteroid-regulated 16 kDa protein	MW800722	3.38
c35194_g4_i2	ARM	*Ov-C2CD2*	C2 domain containing protein 2 ×2	MW800723	3.69
c32350_g3_i1	ARM	*Ov-nAChRalpha1*	Acetylcholine receptor subunit alpha-like 1	MW800709	3.71
c29941_g6_i1	ARM	*Ov-14-3-3zeta*	14–3-3 protein zeta	MW800678	4.27
c36050_g13_i1	ARM	*Ov-Sdhd*	Succinate dehydrogenase ubiquinone cytochrome b small subunit, mitochondrial	MW800689	5.11
c34295_g8_i1	ARM	*Ov-Vbp1*	Prefoldin subunit 3	MW800685	5.33
c34563_g2_i1	ARM	*Ov-PCK1*	Phosphoenolpyruvate carboxykinase cytosolic GTP	MW800686	5.51
c35194_g4_i1	ARM	*Ov-C2CD2*	C2 domain containing protein 2 ×1	MW800687	5.53
c35789_g7_i1	ARM	*Ov-MRM2*	rRNA methyltransferase 2, mitochondrial	MW800688	6.77
c32876_g12_i1	ARM	*Ov-RNF7*	RING-box protein 2	MW800710	6.90
c30691_g3_i1	ARM	*Ov-RSU1*	Ras suppressor protein 1	MW800679	7.13
c31105_g4_i1	ARM	*Ov-BTBD2*	BTB/POZ domain-containing protein 2	MW800680	7.22
c26803_g1_i1	ARM	*Ov-RAD23B*	UV excision repair protein RAD23 homolog B	MW800690	7.25
c28934_g1_i1	ARM	*Ov-UGP2*	UTP––glucose-1-phosphate uridylyltransferase	MW800692	7.39
c28702_g2_i1	ARM	*Ov-Abhd18*	Protein ABHD18	MW800691	8.08
c33117_g3_i1	ARM	*Ov-Rnd3*	Rho-related GTP-binding protein RhoE	MW800683	8.09
c32876_g7_i5	ARM	*Ov-KCMF1*	E3 ubiquitin-protein ligase KCMF1	MW800682	9.26
c33305_g9_i1	ARM	*Ov-Abi2*	Abl interactor 2	MW800684	10.22
c32222_g5_i1	ARM	*Ov-UBE2F*	NEDD8-conjugating enzyme UBE2F	MW800681	11.77
c34071_g2_i1	BRAIN	*Ov-mts*	Serine/threonine protein phosphatase PP2A	MW800693	0.43
c28771_g3_i1	BRAIN	*Ov-AP5Z1*	AP-5 complex subunit zeta-1	MW800696	0.99
c34716_g8_i1	BRAIN	*Ov-USP15*	Ubiquitin carboxyl-terminal hydrolase 15	MW800700	1.29
c35771_g14_i2	BRAIN	*Ov-Gnaq*	Guanine nucleotide-binding protein G(q) subunit alpha	MW800695	1.60
c35361_g5_i1	BRAIN	*Ov-Fam160a2*	FTS and hook-interacting protein-like	MW800703	1.93
c34932_g8_i1	BRAIN	*Ov-MTX1*	Metaxin-1	MW800712	2.14
c34844_g11_i1	BRAIN	*Ov-WBP2*	WW domain-binding protein 2	MW800701	2.39
c30165_g11_i1	BRAIN	*Ov-wls*	Protein wntless	MW800721	3.05
c31295_g14_i1	BRAIN	*Ov-AP1M1*	AP-1 complex subunit mu-1	MW800711	3.05
c35016_g13_i1	BRAIN	*Ov-Gsk3b*	Glycogen synthase kinase-3 beta	MW800694	3.19
c35896_g5_i1	BRAIN	*Ov-Snx25*	Sorting nexin-25	MW800705	3.36
c35327_g8_i2	BRAIN	*Ov-FBXO38*	F-box only protein 38	MW800708	3.53
c35373_g3_i2	BRAIN	*Ov-Snx20*	Sorting nexin-20	MW800704	3.65
c30947_g6_i1	BRAIN	*Ov-syvn1*	E3 ubiquitin-protein ligase synoviolin	MW800698	3.85
c32955_g4_i1	BRAIN	*Ov-Homer2*	Homer protein homolog 2	MW800699	4.14
c35037_g6_i2	BRAIN	*Ov-PIP4K2B*	Phosphatidylinositol 5-phosphate 4-kinase type-2 beta	MW800702	4.94
c34087_g16_i1	BRAIN	*Ov-CSDE1*	Cold shock domain-containing protein E1	MW800672	5.09
c36137_g10_i4	BRAIN	*Ov-AGL*	Glycogen debranching enzyme	MW800706	5.44
c29565_g1_i1	BRAIN	*Ov-CERK*	Ceramide kinase	MW800697	5.52
c34695_g13_i5	BRAIN	*Ov-CNBP*	Cellular nucleic acid-binding protein	MW800707	5.96
***from previously published studies***					
c26807_g1_i1	Previously published	*Ov-eef1a*	Elongation factor 1-alpha (Xu and Zheng, 2018)	MW800714	16.81
c2281_g1_i1	Previously published	*Ov-Rpl6*	60S ribosomal protein L6 (Xu and Zheng, 2018)	MW800718	24.75
c5816_g1_i1	Previously published	*Ov-Rps27a*	Ubiquitin-40S ribosomal protein S27a (Sirakov et al., 2009)	MW800713	30.40
c29373_g3_i1	Previously published	*Ov-RPS18*	40S ribosomal protein S18 (Imperadore, 2017)	MW800720	33.43
c12855_g1_i1	Previously published	*Ov-TUBG1*	Tubulin gamma-1 chain (Xu and Zheng, 2018)	MW800715	36.73
c34110_g1_i1	Previously published	*Ov-MRPS5*	28S ribosomal protein S5, mitochondrial (Xu and Zheng, 2018)	MW800716	38.56
c30772_g3_i11	Previously published	*Ov-RpL23*	60S ribosomal protein L23 (Imperadore, 2017)	MW800719	42.20
c36025_g3_i2	Previously published	*Ov-Tuba1a*	Tubulin alpha-1A chain (Sirakov et al., 2009)	MW800717	80.40

List and details of the candidate reference genes for *Octopus vulgaris* identified and validated in this study (see the main text for details). Highlighted transcripts are those that have been identified as stable in different tissue groups. CV, Coefficient of Variation.

Eight RGs from previous studies on cephalopods (Sirakov et al., 2009; García-Fernández et al., 2016; Baldascino et al., 2017; Imperadore, 2017; Xu and Zheng, 2018; Whang et al., 2020; see also Table 1 and Supplementary Table 2) were also included for subsequent validation analyses.

### Sample collection and processing for RT-qPCR

To test the selected candidate RGs, tissues were harvested from five adult specimens of *O. vulgaris* (Supplementary Table 3) that did not show any signs of lesions, aberrant formations, or regenerating parts. From each octopus, 12 tissues were collected: SEM; SUB; OL; GG; StG; a portion of muscle from the ventral side of the mantle (MANT); arm tips from the anterior (Tip_R1) and posterior arms (Tip_R4); ARM_R1 and ARM_R4; MUSC_R1; and the left gill (GILL), considered here as a reference tissue for an internal organ differing from the muscles and nervous structures (Figure 1). The tissues were processed for RNA extraction; RNA integrity was tested using Agilent Bioanalyzer 2100 (see Supplementary Figure 2) and cDNA synthesis; cDNA samples were stored at −20°C until use (see Supplementary Info for specimens handling, sample harvesting, tissue processing, and RNA and cDNA processing and synthesis).

### Primer design and amplification efficiency analysis for qRT-PCR

Primer3 Plus software (Untergasser et al., 2012) was used to design specific primers (Supplementary Table 4) to amplify the candidate genes. The following parameters were utilized: optimal melting temperature at 60°C, amplicon size 100–200 bp, and primer size between 18 and 27 bp (optimum set at 20 bp). Template RNA sequences were retrieved from previously mentioned RNA-seq studies. To obtain the most efficient primer couples, hairpin, homodimer, and heterodimer structures were evaluated for each primer couple using the Multiple Primer Analyzer^1^ (modified after Breslauer et al., 1986). In addition, 12 primer couples from eight genes were selected from the literature and slightly modified, when needed, to match with *O. vulgaris* sequences, or they were designed *ex novo* based on published ones (Supplementary Table 4).

RT-qPCR was performed on four-fold cDNA dilutions (from 10 ng/μL to 0.15625 ng/μL; see Supplementary Info) to calculate the primers’ efficiency, using the formula $E=\left[\left(10^{-1}/m\right)-1\right]\times 100$ where m is the slope of the linear interpolation of dots representing Ct in the function of log_10_ [cDNA concentration].

To estimate the gene expression in each tissue, the primers were tested for RT-qPCR on individual samples in technical triplicates by using 2 μl cDNA [1.25 ng/μl] (see Supplementary Info for details).

### Expression stability

The 12 tissues included in this study are highly diverse in structure, function, and gene expression profile. Thus, to account for this variability (with highest variability showed by the arm tips), we considered three groups for the expression stability analyses: **Nervous** (SEM, SUB, OL, GG, and StG); all tissues excluding the arm tips (**Allex:** Nervous, plus GILL, MANT, ARM_R1, ARM_R4, and MUSC_R1); and **Adult** (all the tissues including the arm tips; see also above and Figure 1).

The expression stability of each candidate gene across all samples within each tissue group was investigated using the mean Ct values and four different algorithms: *geNorm* (Vandesompele et al., 2002), *NormFinder* (Mestdagh et al., 2009), *BestKeeper* (Pfaffl et al., 2004), and the Delta-CT method (2^−ΔΔCT^) (Livak and Schmittgen, 2001). *geNorm* estimates the average pairwise variation in a specific gene with all the other potential reference genes. *NormFinder* computes the stability value for each gene according to their minimum variance. Both the *geNorm* and *NormFinder* values are lower for more stable genes (Amable et al., 2013). *BestKeeper* relies on the concept that the more stable the gene expression, the lower the Ct variation if the cDNA quantity is constant (Amable et al., 2013). Finally, the Delta Ct algorithm (Livak and Schmittgen, 2001) takes into account the expression of each gene in all samples and its standard deviation (SD); the gene with the lowest SD is considered the most appropriate reference gene (Silver et al., 2006).

The results from these approaches were integrated in *RefFinder* (Xie et al., 2012) to obtain an overall rank of expression stability for each of the three tissue groups. The method ranks each gene in each group and calculates the geometric mean of ranks for each gene. More stable genes show smaller geometric means, as they are ranked higher by all the methods.

### Validation of reference genes

To validate the reliability of the data normalization, the combination of the two most stable candidate RGs, and of the most stable and unstable reference genes for the **Nervous** group were used to analyze the expression levels of the target genes. When two RGs were utilized for normalization, we relied on their geometric mean. The relative quantification of nine target genes was calculated for the **Nervous** group following Pfaffl's (2001) method, which takes into account the primer efficiencies of both targets and RGs.

### Data analysis

Following Zar (1999), statistical significance was assessed after an ANOVA test, followed by Bonferroni multiple comparison tests. For all analyses, we used SPSS (rel. 18.0, SPSS Inc. - Chicago, 2009), with the exceptions mentioned above. All tests were two-tailed, and the alpha was set at 0.05.

## Results

### *In silico* identification of candidate reference genes

Candidate RGs were identified from the transcriptome of *O. vulgaris* (Petrosino, 2015; Petrosino et al., 2022). Genes with a relatively stable expression *in silico* in four tissue groups (**Adult**, **Brain**, **Nervous, and Arm**) were selected according to the relative abundance of each transcript (TPM counts) and their coefficient of variation (CV). Using a CV cut-off of 20%, 32 transcripts (out a total of 64,477 unique transcripts) were selected for **Adult**. The CV cut-off was decreased to 15% to identify the most stable transcripts in the **Brain** (1,540 transcripts), **Nervous** system (357), and **Arm** (125). A total of 2,145 transcripts was identified. Because the annotation results were not further curated in the original studies (Musacchia et al., 2015; Petrosino, 2015; Petrosino et al., 2015, 2022), we excluded non-annotated transcripts, thus identifying 88 potential RGs for the four tissue groups (Table 1). Seven of them resulted shared among more than one group (highlighted in Table 1).

We observed the highest variability in CVs among samples belonging to the **Adult** group (19 genes; mean CV = 13.6%; CV range: 8.8–16.2%; Table 1). In this set, 12 genes showed CV values lower than 15% (i.e., *Ov-UBE2F*, *Ov-PRMT5*, *Ov-LTV1*, *Ov-CPIJ005834*, *Ov-EIF2A*, *Ov-rpf1*, *Ov-slc25a40*, *Ov-RIOK2*, *Ov-Dap3*, *Ov-Ppm1b*, *Ov-ATPAF2*, and *Ov-NOB1*; Table 1). Lower CV values were observed when the **Brain** group was considered (20 genes; mean CV = 3.28%; CV range: 0.4–6.0%; Table 1), with seven genes exhibiting CV values below 3% (i.e., *Ov-mts*, *Ov-AP5Z1*, *Ov-USP15*, *Ov-Gnaq*, *Ov-Fam160a2*, *Ov-MTX1*, and *Ov-WBP2*; Table 1). Nineteen candidate RGs were identified for the **Nervous** group (mean CV = 6.5%; CV range: 3.9–9.9%; Table 1), with average CVs higher than those observed for the **Brain** group. In this case, 12 genes had CV values lower than 7% (i.e., *Ov-Gsk3b*, *Ov-mts*, *Ov-timm*, *Ov-SUCLG2*, *Ov-CHCHD7*, *Ov-UBE2F*, *Ov-MTX1*, *Ov-gk5*, *Ov-Gnaq*, *Ov-Naa15*, *Ov-wdr44*, and *Ov-Klhdc*). For the **Arm** group (19 genes; mean CV = 6.7%; CV range: 3.4–11.8%), we observed similar CVs to the **Nervous** group, with 9 genes (10 transcripts) having CVs lower than 7% (i.e., *Ov-ESR16*, *Ov-C2CD2*, *Ov-nAChRalpha1*, *Ov-14-3-3zeta*, *Ov-Sdhd*, *Ov-Vbp1*, *Ov-PCK1*, *Ov-MRM2,* and *Ov-RNF7*; Table 1).

A total of 69 candidate RGs were selected for biological validation. In addition, we considered eight RGs used in previous studies (Sirakov et al., 2009; Imperadore, 2017; Xu and Zheng, 2018), raising the final number of genes to be tested through qRT-PCR experiments to 77 (Table 1).

### Candidate reference genes and their expression profiles

Eighty-one primer couples for the selected putative RGs were designed and tested for specificity and efficiency through standard PCR and qRT-PCR reactions (Supplementary Info), using the total mRNA extracted from 12 tissues (Figure 1) belonging to five *O. vulgaris* specimens. Three primer couples (i.e., *Ov-wls*, *Ov-ESR16*, and *Ov-C2CD2* isoform X2) exhibited no or multiple amplification products when tested for standard PCR and were excluded from subsequent analyses. All other primer couples resulted in a single amplification product at the expected amplicon size (Supplementary Figure 3) and were therefore tested for RT-qPCR. The primer sequences, amplicon size, product Tm, and amplification efficiencies are shown in Supplementary Table 4.

A total of 59 primer pairs showed amplification efficiencies between 98 and 102%, while 19 did not fall within this range and were excluded from further analyses (Supplementary Table 4).

The expression levels of the final list of candidate RGs (n = 59) were estimated in each tissue sample (technical triplicates) through qRT-PCR. The reference genes displayed a wide range of transcription levels, with average Ct values ranging from 18.17 to 37.27 (Supplementary Table 5). *Ov-Tuba1a* showed the lowest mean Ct (21.63), i.e., the highest abundance in tissues. High expression levels were also noted for *Ov-Rpl6*, *Ov-tollip,* and *Ov-RPS18* (mean Ct = 24.15, 24.40, and 24.79, respectively). In an opposite trend, *Ov-NOB1* and *Ov-TUBG1-FR* presented a relatively low expression level (mean Ct = 31.31 in both cases; Supplementary Table 5).

### Analysis of expression stability of the candidate reference genes

For the expression stability analyses, three tissue groups of increasing biological variability were considered (**Nervous**, **ALLex**, and **Adult**).

Our results suggested that the most suitable reference genes differed among the approaches used for the identification of RGs (see Methods), as well as among the groups considered, likely owing to their substantial tissue diversity (Table 2).

**Table 2:** Outcomes of the analysis for reference genes stability after application of the four algorithms (i.e., *GeNorm*, *NormFinder*, *BestKeeper*, and DeltaCt).

	*GeNorm* Algorithm						*NormFinder* algorithm					
	Nervous		Allex		Adult		Nervous		Allex		Adult	
	Gene name	Stab. value	Gene name	Stab. value	Gene name	Stab. value	Gene name	Stab. value	Gene name	Stab. value	Gene name	Stab. value
1	***Ov-CHCHD7**/* ***Ov-RNF7***	0.271	***Ov-CUL1**/* ***Ov-Naa15***	0.386	***Ov-Vbp1***	0.544	***Ov-RIOK2***	0.238	***Ov-EIF2A***	0.371	***Ov-EIF2A***	0.382
2	***Ov-RIOK2***	0.297	***Ov-RIOK2***	0.480	***Ov-EIF2A***	0.556	***Ov-slc25a40***	0.278	***Ov-Naa15***	0.373	***Ov-CUL1***	0.390
3	***Ov-UBE2F***	0.317	***Ov-slc25a40***	0.520	***Ov-RAD23B***	0.573	*Ov-RNF7*	0.296	***Ov-Ppm1b***	0.376	***Ov-Ppm1b***	0.469
4	***Ov-slc25a40***	0.333	***Ov-usp10***	0.534	***Ov-syvn1***	0.603	*Ov-CHCHD7*	0.299	***Ov-CUL1***	0.388	***Ov-Vbp1***	0.505
5	*Ov-BTBD17*	0.355	***Ov-KCMF1***	0.544	***Ov-CUL1***	0.625	***Ov-Ppm1b***	0.330	***Ov-RIOK2***	0.411	***Ov-syvn1***	0.527
6	***Ov-Naa15***	0.372	***Ov-Ppm1b***	0.555	***Ov-Ppm1b***	0.674	***Ov-syvn1***	0.336	***Ov-KCMF1***	0.444	***Ov-slc25a40***	0.584
7	***Ov-Ppm1b***	0.396	***Ov-EIF2A***	0.562	***Ov-CHCHD7***	0.691	***Ov-UBE2F***	0.340	***Ov-usp10***	0.477	***Ov-UBE2F***	0.597
8	***Ov-CUL1***	0.415	*Ov-EIF3M*	0.576	***Ov-RIOK2***	0.707	***Ov-Naa15***	0.349	*Ov-EIF3M*	0.495	***Ov-RAD23B***	0.599
9	*Ov-Snx25*	0.432	***Ov-CHCHD7***	0.611	***Ov-UBE2F***	0.722	*Ov-BTBD17*	0.354	***Ov-syvn1***	0.499	*Ov-Rnd3*	0.627
10	*Ov-Dnaja3*	0.445	*Ov-syvn1*	0.619	***Ov-RNF7***	0.738	*Ov-Abi2*	0.373	***Ov-slc25a40***	0.500	***Ov-RIOK2***	0.637
11	*Ov-usp10*	0.454	*Ov-Vbp1*	0.627	*Ov-Rnd3*	0.769	*Ov-EIF2A*	0.374	*Ov-Vbp1*	0.505	*Ov-CHCHD7*	0.680
12	*Ov-Fam160a2*	0.462	*Ov-RAD23B*	0.644	*Ov-syvn1*	0.782	*Ov-Ltv1*	0.399	*Ov-CHCHD7*	0.538	*Ov-Dap3*	0.686
13	*Ov-PTPN12*	0.470	*Ov-UBE2F*	0.653	*Ov-MRM2*	0.805	*Ov-usp10*	0.405	*Ov-RAD23B*	0.539	*Ov-MRM2*	0.687
14	*Ov-ATPAF2*	0.478	*Ov-Dap3*	0.661	*Ov-Fam160a2*	0.814	*Ov-AP5Z1*	0.406	*Ov-Dap3*	0.558	*Ov-usp10*	0.689
15	*Ov-AP5Z1*	0.485	*Ov-ATPAF2*	0.668	*Ov-usp10*	0.824	*Ov-CUL1*	0.419	*Ov-UBE2F*	0.562	*Ov-RNF7*	0.696
16	*Ov-syvn1*	0.491	*Ov-Rnd3*	0.675	*Ov-Dap3*	0.834	*Ov-EIF3M*	0.420	*Ov-ATPAF2*	0.574	*Ov-gk5*	0.697
17	*Ov-EIF2A*	0.498	*Ov-RNF7*	0.682	*Ov-gk5*	0.843	*Ov-CSDE1*	0.422	*Ov-MRPS5_FR*	0.585	*Ov-Fam160a2*	0.735
18	*Ov-Abi2*	0.503	*Ov-Ltv1*	0.689	*Ov-ATPAF2*	0.854	*Ov-PTPN12*	0.423	*Ov-MRM2*	0.592	*Ov-Naa15*	0.740
19	*Ov-KCMF1*	0.508	*Ov-MRM2*	0.695	*Ov-CG9286*	0.863	*Ov-Snx25*	0.424	*Ov-Rnd3*	0.595	*Ov-CPIJ005834*	0.762
20	*Ov-Ltv1*	0.515	*Ov-Fam160a2*	0.703	*Ov-Ltv1*	0.879	*Ov-BTBD2*	0.438	*Ov-Ltv1*	0.606	*Ov-Ltv1*	0.762
21	*Ov-CSDE1*	0.520	*Ov-MRPS5_FR*	0.710	*Ov-CPIJ005834*	0.886	*Ov-KCMF1*	0.439	*Ov-RNF7*	0.612	*Ov-Sdhd*	0.766
22	*Ov-prrc1*	0.525	*Ov-MRPS5_F1R1*	0.732	*Ov-Naa15*	0.893	*Ov-MRPS5_FR*	0.457	*Ov-Fam160a2*	0.646	*Ov-CG9286*	0.799
23	*Ov-BTBD2*	0.529	*Ov-CG9286*	0.742	*Ov-Sdhd*	0.900	*Ov-Rnd3*	0.457	*Ov-MRPS5_F1R1*	0.660	*Ov-ATPAF2*	0.806
24	*Ov-tollip*	0.534	*Ov-gk5*	0.751	*Ov-EIF3M*	0.915	*Ov-Dnaja3*	0.461	*Ov-gk5*	0.703	*Ov-EIF3M*	0.849
25	*Ov-EIF3M*	0.540	*Ov-mts*	0.768	*Ov-prrc1*	0.925	*Ov-Vbp1*	0.464	*Ov-CG9286*	0.729	*Ov-MRPS5_FR*	0.856
26	*Ov-Rnd3*	0.545	*Ov-CPIJ005834*	0.778	*Ov-Abhd18*	0.954	*Ov-ATPAF2*	0.478	*Ov-mts*	0.744	*Ov-prrc1*	0.863
27	*Ov-MRPS5_FR*	0.551	*Ov-prrc1*	0.787	*Ov-MRPS5_FR*	0.965	*Ov-MRM2*	0.486	*Ov-CPIJ005834*	0.771	*Ov-Abhd18*	0.890
28	*Ov-Vbp1*	0.558	*Ov-PTPN12*	0.797	*Ov-KCMF1*	0.976	*Ov-prrc1*	0.489	*Ov-PTPN12*	0.774	*Ov-KCMF1*	0.951
29	*Ov-Dap3*	0.565	*Ov-RPS18*	0.807	*Ov-C2CD2*	0.998	*Ov-Fam160a2*	0.502	*Ov-prrc1*	0.802	*Ov-rpf1*	0.969
30	*Ov-CG9286*	0.571	*Ov-Sdhd*	0.817	*Ov-UGP2*	1.009	*Ov-tollip*	0.504	*Ov-Sdhd*	0.809	*Ov-MRPS5_F1R1*	0.974
31	*Ov-MRM2*	0.578	*Ov-Abhd18*	0.837	*Ov-CSDE1*	1.018	*Ov-Dap3*	0.513	*Ov-RPS18*	0.835	*Ov-SUCLG2*	0.997
32	*Ov-UGP2*	0.585	*Ov-SUCLG2*	0.870	*Ov-rpf1*	1.027	*Ov-CG9286*	0.546	*Ov-Abhd18*	0.862	*Ov-CSDE1*	1.034
33	*Ov-RAD23B*	0.592	*Ov-tollip*	0.880	*Ov-MRPS5_F1R1*	1.046	*Ov-UGP2*	0.568	*Ov-tollip*	0.916	*Ov-timm*	1.035
34	*Ov-Sdhd*	0.599	*Ov-Abi2*	0.891	*Ov-Abi2*	1.055	*Ov-RAD23B*	0.578	*Ov-Abi2*	0.926	*Ov-C2CD2*	1.037
35	*Ov-WBP2*	0.606	*Ov-rpf1*	0.902	*Ov-SUCLG2*	1.064	*Ov-gk5*	0.579	*Ov-SUCLG2*	0.928	*Ov-UGP2*	1.048
36	*Ov-gk5*	0.614	*Ov-RpL23*	0.935	*Ov-timm*	1.073	*Ov-WBP2*	0.611	*Ov-rpf1*	0.972	*Ov-WBP2*	1.056
37	*Ov-RPS18*	0.621	*Ov-C2CD2*	0.957	*Ov-WBP2*	1.093	*Ov-Sdhd*	0.616	*Ov-RpL23*	1.023	*Ov-Abi2*	1.063
38	*Ov-mts*	0.630	*Ov-CSDE1*	0.969	*Ov-PTPN12*	1.103	*Ov-RPS18*	0.623	*Ov-C2CD2*	1.034	*Ov-PTPN12*	1.134
39	*Ov-MRPS5_F1R1*	0.641	*Ov-UGP2*	0.990	*Ov-BTBD17*	1.115	*Ov-MRPS5_F1R1*	0.702	*Ov-NOB1*	1.069	*Ov-BTBD17*	1.181
40	*Ov-CPIJ005834*	0.651	*Ov-NOB1*	1.000	*Ov-AP5Z1*	1.127	*Ov-mts*	0.709	*Ov-timm*	1.071	*Ov-AP5Z1*	1.233
41	*Ov-TUBG1_FR*	0.661	*Ov-timm*	1.010	*Ov-mts*	1.140	*Ov-CPIJ005834*	0.727	*Ov-CSDE1*	1.077	*Ov-mts*	1.277
42	*Ov-C2CD2*	0.674	*Ov-WBP2*	1.020	*Ov-Snx25*	1.179	*Ov-TUBG1_FR*	0.745	*Ov-WBP2*	1.082	*Ov-RPS18*	1.304
43	*Ov-SUCLG2*	0.690	*Ov-Snx25*	1.029	*Ov-tollip*	1.193	*Ov-C2CD2*	0.825	*Ov-UGP2*	1.113	*Ov-Snx25*	1.369
44	*Ov-rpf1*	0.705	*Ov-BTBD17*	1.052	*Ov-BTBD2*	1.206	*Ov-PCK1*	0.888	*Ov-Snx25*	1.122	*Ov-tollip*	1.378
45	*Ov-PCK1*	0.720	*Ov-Dnaja3*	1.063	*Ov-AGL*	1.231	*Ov-rpf1*	0.894	*Ov-Dnaja3*	1.211	*Ov-PCK1*	1.382
46	*Ov-Tuba1a*	0.738	*Ov-Tuba1a*	1.075	*Ov-RPS18*	1.245	*Ov-SUCLG2*	0.919	*Ov-Tuba1a*	1.216	*Ov-BTBD2*	1.398
47	*Ov-AGL*	0.758	*Ov-AP5Z1*	1.087	*Ov-PIP4K2B*	1.271	*Ov-Tuba1a*	1.035	*Ov-BTBD17*	1.216	*Ov-AGL*	1.425
48	*Ov-Abhd18*	0.777	*Ov-TUBG1_FR*	1.100	*Ov-PCK1*	1.284	*Ov-AGL*	1.061	*Ov-TUBG1_FR*	1.267	*Ov-PIP4K2B*	1.450
49	*Ov-RpL23*	0.798	*Ov-Homer2*	1.112	*Ov-Dnaja3*	1.298	*Ov-Abhd18*	1.095	*Ov-AP5Z1*	1.284	*Ov-Dnaja3*	1.544
50	*Ov-NOB1*	0.819	*Ov-BTBD2*	1.127	*Ov-Snx20*	1.314	*Ov-RpL23*	1.175	*Ov-Homer2*	1.312	*Ov-Snx20*	1.551
51	*Ov-Rpl6*	0.842	*Ov-AGL*	1.141	*Ov-NOB1*	1.330	*Ov-NOB1*	1.252	*Ov-PIP4K2B*	1.386	*Ov-NOB1*	1.576
52	*Ov-Homer2*	0.867	*Ov-PIP4K2B*	1.155	*Ov-Homer2*	1.346	*Ov-Rpl6*	1.283	*Ov-PCK1*	1.403	*Ov-TUBG1_FR*	1.655
53	*Ov-timm*	0.893	*Ov-PCK1*	1.169	*Ov-wdr44*	1.361	*Ov-Homer2*	1.365	*Ov-BTBD2*	1.437	*Ov-Homer2*	1.672
54	*Ov-TUBG1_F1R1*	0.920	*Ov-wdr44*	1.183	*Ov-TUBG1_FR*	1.396	*Ov-timm*	1.460	*Ov-AGL*	1.442	*Ov-wdr44*	1.684
55	*Ov-Snx20*	0.953	*Ov-TUBG1_F1R1*	1.197	*Ov-TUBG1_F1R1*	1.413	*Ov-TUBG1_F1R1*	1.512	*Ov-TUBG1_F1R1*	1.442	*Ov-TUBG1_F1R1*	1.719
56	*Ov-PIP4K2B*	0.990	*Ov-Snx20*	1.212	*Ov-RpL23*	1.434	*Ov-Snx20*	1.699	*Ov-wdr44*	1.443	*Ov-RpL23*	1.916
57	*Ov-wdr44*	1.031	*Ov-Rps27a_FR*	1.261	*Ov-Rpl6*	1.465	*Ov-PIP4K2B*	1.888	*Ov-Snx20*	1.535	*Ov-Rpl6*	2.370
58	*Ov-Rps27a_FR*	1.090	*Ov-Rpl6*	1.297	*Ov-Rps27a_FR*	1.500	*Ov-wdr44*	2.035	*Ov-Rps27a_FR*	2.047	*Ov-Rps27a_FR*	2.539
59					*Ov-Tuba1a*	1.537	*Ov-Rps27a_FR*	2.687	*Ov-Rpl6*	2.443	*Ov-Tuba1a*	2.677

	*BestKeeper* algorithm						Delta Ct method					
	Nervous		Allex		Adult		Nervous		Allex		Adult	
	**Gene name**	Std. dev	Gene name	Std. dev	Gene name	Std. dev	Gene name	Std. dev	Gene name	Std. dev	Gene name	Std. dev
1	***Ov-Abi2***	0.610	***Ov-Ltv1***	0.850	***Ov-Ltv1***	0.750	***Ov-RIOK2***	0.780	***Ov-Naa15***	1.020	***Ov-EIF2A***	1.190
2	***Ov-RPS18***	0.610	***Ov-RPS18***	0.910	***Ov-RpL23***	0.750	*Ov-RNF7*	0.790	***Ov-EIF2A***	1.020	***Ov-CUL1***	1.190
3	***Ov-RpL23***	0.630	***Ov-Abi2***	0.920	***Ov-RPS18***	0.800	***Ov-slc25a40***	0.790	***Ov-Ppm1b***	1.020	***Ov-Ppm1b***	1.210
4	***Ov-EIF3M***	0.640	***Ov-RpL23***	0.930	*Ov-prrc1*	0.820	*Ov-CHCHD7*	0.790	***Ov-CUL1***	1.030	***Ov-Vbp1***	1.240
5	*Ov-RNF7*	0.690	***Ov-EIF3M***	0.940	***Ov-gk5***	0.860	***Ov-UBE2F***	0.810	***Ov-RIOK2***	1.040	***Ov-syvn1***	1.240
6	***Ov-BTBD17***	0.700	***Ov-BTBD17***	0.980	***Ov-BTBD17***	0.860	***Ov-Ppm1b***	0.820	*Ov-KCMF1*	1.050	***Ov-slc25a40***	1.260
7	*Ov-Sdhd*	0.710	***Ov-RIOK2***	0.990	*Ov-slc25a40*	0.890	***Ov-Naa15***	0.820	***Ov-usp10***	1.060	***Ov-UBE2F***	1.270
8	*Ov-MRM2*	0.730	***Ov-gk5***	1.010	*Ov-Rnd3*	0.900	*Ov-BTBD17*	0.820	*Ov-EIF3M*	1.080	***Ov-RAD23B***	1.280
9	*Ov-RAD23B*	0.740	*Ov-NOB1*	1.030	*Ov-CPIJ005834*	0.930	***Ov-syvn1***	0.820	***Ov-syvn1***	1.080	*Ov-Rnd3*	1.290
10	*Ov-CHCHD7*	0.760	*Ov-MRPS5_F1R1*	1.050	***Ov-EIF3M***	0.940	***Ov-EIF2A***	0.830	***Ov-Vbp1***	1.080	***Ov-RIOK2***	1.300
11	*Ov-syvn1*	0.770	*Ov-Rnd3*	1.060	*Ov-Abi2*	0.940	*Ov-Abi2*	0.830	*Ov-slc25a40*	1.080	*Ov-usp10*	1.300
12	*Ov-EIF2A*	0.770	*Ov-UBE2F*	1.100	*Ov-MRPS5_F1R1*	0.950	*Ov-usp10*	0.850	*Ov-CHCHD7*	1.090	*Ov-MRM2*	1.310
13	*Ov-MRPS5_F1R1*	0.770	*Ov-RAD23B*	1.130	*Ov-RIOK2*	0.960	*Ov-CUL1*	0.850	*Ov-UBE2F*	1.100	*Ov-Dap3*	1.310
14	*Ov-RIOK2*	0.800	*Ov-CPIJ005834*	1.130	*Ov-usp10*	0.970	*Ov-EIF3M*	0.850	*Ov-RAD23B*	1.100	*Ov-CHCHD7*	1.320
15	*Ov-Ltv1*	0.810	*Ov-EIF2A*	1.140	*Ov-NOB1*	1.000	*Ov-Ltv1*	0.860	*Ov-Dap3*	1.110	*Ov-RNF7*	1.320
16	*Ov-CPIJ005834*	0.810	*Ov-syvn1*	1.150	*Ov-BTBD2*	1.000	*Ov-AP5Z1*	0.860	*Ov-ATPAF2*	1.120	*Ov-gk5*	1.330
17	*Ov-UBE2F*	0.810	*Ov-slc25a40*	1.170	*Ov-UBE2F*	1.000	*Ov-PTPN12*	0.860	*Ov-MRM2*	1.130	*Ov-Fam160a2*	1.330
18	*Ov-Vbp1*	0.810	*Ov-Sdhd*	1.170	*Ov-KCMF1*	1.020	*Ov-Snx25*	0.860	*Ov-Rnd3*	1.130	*Ov-Naa15*	1.350
19	*Ov-BTBD2*	0.850	*Ov-CUL1*	1.180	*Ov-MRM2*	1.020	*Ov-CSDE1*	0.870	*Ov-Ltv1*	1.130	*Ov-Sdhd*	1.360
20	*Ov-Rnd3*	0.850	*Ov-usp10*	1.200	*Ov-EIF2A*	1.040	*Ov-KCMF1*	0.870	*Ov-MRPS5_FR*	1.130	*Ov-Ltv1*	1.360
21	*Ov-NOB1*	0.860	*Ov-prrc1*	1.230	*Ov-Sdhd*	1.040	*Ov-Dnaja3*	0.870	*Ov-RNF7*	1.130	*Ov-CPIJ005834*	1.370
22	*Ov-MRPS5_FR*	0.870	*NAA15*	1.230	*NAA15*	1.050	*Ov-MRPS5_FR*	0.880	*Ov-Fam160a2*	1.150	*Ov-ATPAF2*	1.380
23	*Ov-UGP2*	0.890	*Ov-MRM2*	1.230	*Ov-Dap3*	1.060	*Ov-BTBD2*	0.880	*Ov-MRPS5_F1R1*	1.170	*Ov-CG9286*	1.380
24	*Ov-WBP2*	0.890	*Ov-MRPS5_FR*	1.250	*Ov-CSDE1*	1.060	*Ov-Rnd3*	0.880	*Ov-gk5*	1.190	*Ov-prrc1*	1.410
25	*Ov-slc25a40*	0.890	*Ov-timm*	1.260	*Ov-UGP2*	1.070	*Ov-ATPAF2*	0.890	*Ov-mts*	1.210	*Ov-EIF3M*	1.410
26	*Ov-Ppm1b*	0.890	*Ov-UGP2*	1.280	*Ov-syvn1*	1.080	*Ov-Vbp1*	0.890	*Ov-CG9286*	1.210	*Ov-MRPS5_FR*	1.440
27	*Ov-prrc1*	0.900	*Ov-rpf1*	1.290	*Ov-CUL1*	1.080	*Ov-prrc1*	0.900	*Ov-PTPN12*	1.230	*Ov-Abhd18*	1.450
28	*Ov-Tuba1a*	0.900	*Ov-BTBD2*	1.300	*Ov-CG9286*	1.090	*Ov-tollip*	0.900	*Ov-CPIJ005834*	1.240	*Ov-KCMF1*	1.450
29	*Ov-timm*	0.910	*Ov-Vbp1*	1.300	*Ov-C2CD2*	1.110	*Ov-Fam160a2*	0.900	*Ov-prrc1*	1.250	*Ov-rpf1*	1.500
30	*Ov-AP5Z1*	0.920	*Ov-Dap3*	1.310	*Ov-RAD23B*	1.130	*Ov-MRM2*	0.900	*Ov-Sdhd*	1.250	*Ov-MRPS5_F1R1*	1.510
31	*Ov-gk5*	0.930	*CHCHD*	1.330	*Ov-ATPAF2*	1.140	*Ov-Dap3*	0.920	*Ov-RPS18*	1.280	*Ov-CSDE1*	1.510
32	*Ov-CG9286*	0.940	*Ov-CG9286*	1.330	*Ov-rpf1*	1.160	*Ov-CG9286*	0.940	*Ov-Abhd18*	1.310	*Ov-C2CD2*	1.510
33	*Ov-Naa15*	0.940	*Ov-CSDE1*	1.330	*Ov-Dnaja3*	1.160	*Ov-UGP2*	0.940	*Ov-Abi2*	1.320	*Ov-SUCLG2*	1.520
34	*Ov-PCK1*	0.950	*Ov-Ppm1b*	1.330	*Ov-Ppm1b*	1.160	*Ov-RAD23B*	0.950	*Ov-tollip*	1.330	*Ov-UGP2*	1.530
35	*Ov-tollip*	0.960	*Ov-RNF7*	1.340	*Ov-SUCLG2*	1.190	*Ov-gk5*	0.960	*Ov-SUCLG2*	1.350	*Ov-WBP2*	1.540
36	*Ov-CUL1*	0.960	*Ov-PCK1*	1.360	*Ov-MRPS5_FR*	1.190	*Ov-Sdhd*	0.970	*Ov-rpf1*	1.380	*Ov-timm*	1.550
37	*Ov-ATPAF2*	0.960	*Ov-ATPAF2*	1.370	*Ov-Abhd18*	1.200	*Ov-WBP2*	0.980	*Ov-C2CD2*	1.400	*Ov-Abi2*	1.550
38	*Ov-Dap3*	0.970	*Ov-Abhd18*	1.380	*Ov-timm*	1.220	*Ov-RPS18*	0.980	*Ov-RpL23*	1.420	*Ov-PTPN12*	1.580
39	*Ov-TUBG1_FR*	0.980	*Ov-C2CD2*	1.380	*Ov-tollip*	1.230	*Ov-mts*	1.030	*Ov-CSDE1*	1.430	*Ov-BTBD17*	1.650
40	*Ov-usp10*	0.980	*Ov-KCMF1*	1.420	*Ov-RNF7*	1.250	*Ov-MRPS5_F1R1*	1.040	*Ov-WBP2*	1.440	*Ov-AP5Z1*	1.670
41	*Ov-CSDE1*	1.010	*Ov-TUBG1_FR*	1.480	*CHCHD*	1.270	*Ov-CPIJ005834*	1.060	*Ov-NOB1*	1.450	*Ov-mts*	1.670
42	*Ov-PTPN12*	1.020	*Ov-SUCLG2*	1.500	*Ov-Vbp1*	1.280	*Ov-TUBG1_FR*	1.070	*Ov-UGP2*	1.450	*Ov-RPS18*	1.730
43	*Ov-KCMF1*	1.030	*Ov-Rps27a_FR*	1.520	*Ov-Rps27a_FR*	1.300	*Ov-C2CD2*	1.130	*Ov-timm*	1.460	*Ov-Snx25*	1.770
44	*Ov-Snx25*	1.030	*Ov-Fam160a2*	1.540	*Ov-PTPN12*	1.340	*Ov-PCK1*	1.190	*Ov-Snx25*	1.470	*Ov-tollip*	1.770
45	*Ov-Dnaja3*	1.050	*Ov-Dnaja3*	1.650	*Ov-PCK1*	1.390	*Ov-rpf1*	1.190	*Ov-Tuba1a*	1.550	*Ov-BTBD2*	1.780
46	*Ov-Abhd18*	1.070	*Ov-PTPN12*	1.660	*Ov-Fam160a2*	1.420	*Ov-SUCLG2*	1.200	*Ov-BTBD17*	1.560	*Ov-AGL*	1.800
47	*Ov-Fam160a2*	1.080	*Ov-tollip*	1.670	*Ov-AGL*	1.440	*Ov-Tuba1a*	1.290	*Ov-Dnaja3*	1.560	*Ov-PCK1*	1.810
48	*Ov-rpf1*	1.120	*Ov-PIP4K2B*	1.720	*Ov-TUBG1_FR*	1.440	*Ov-AGL*	1.330	*Ov-AP5Z1*	1.590	*Ov-PIP4K2B*	1.840
49	*Ov-TUBG1_F1R1*	1.130	*Ov-AGL*	1.750	*Ov-mts*	1.460	*Ov-Abhd18*	1.360	*Ov-TUBG1_FR*	1.610	*Ov-Dnaja3*	1.910
50	*Ov-Rpl6*	1.140	*Ov-Snx20*	1.770	*Ov-wdr44*	1.490	*Ov-RpL23*	1.410	*Ov-Homer2*	1.630	*Ov-Snx20*	1.930
51	*Ov-SUCLG2*	1.180	*Ov-Rpl6*	1.770	*Ov-PIP4K2B*	1.500	*Ov-NOB1*	1.470	*Ov-PIP4K2B*	1.710	*Ov-NOB1*	1.940
52	*Ov-AGL*	1.230	*Ov-WBP2*	1.800	*Ov-TUBG1_F1R1*	1.500	*Ov-Rpl6*	1.510	*Ov-BTBD2*	1.710	*Ov-Homer2*	2.010
53	*Ov-C2CD2*	1.250	*Ov-TUBG1_F1R1*	1.830	*Ov-WBP2*	1.650	*Ov-Homer2*	1.570	*Ov-PCK1*	1.710	*Ov-TUBG1_FR*	2.020
54	*Ov-mts*	1.250	*Ov-mts*	1.840	*Ov-Rpl6*	1.680	*Ov-timm*	1.660	*Ov-AGL*	1.720	*Ov-wdr44*	2.030
55	*Ov-Homer2*	1.370	*Ov-Tuba1a*	1.850	*Ov-Snx20*	1.700	*Ov-TUBG1_F1R1*	1.710	*Ov-wdr44*	1.750	*Ov-TUBG1_F1R1*	2.080
56	*Ov-Snx20*	1.400	*Ov-wdr44*	1.890	*Ov-Tuba1a*	1.700	*Ov-Snx20*	1.880	*Ov-TUBG1_F1R1*	1.760	*Ov-RpL23*	2.220
57	*Ov-Rps27a_FR*	1.420	*Ov-AP5Z1*	2.020	*Ov-Homer2*	1.820	*Ov-PIP4K2B*	2.050	*Ov-Snx20*	1.820	*Ov-Rpl6*	2.640
58	*Ov-PIP4K2B*	1.660	*Ov-Snx25*	2.060	*Ov-Snx25*	1.860	*Ov-wdr44*	2.180	*Ov-Rps27a_FR*	2.280	*Ov-Rps27a_FR*	2.800
59	*Ov-wdr44*	1.710	*Ov-Homer2*	2.100	*Ov-AP5Z1*	2.050	*Ov-Rps27a_FR*	2.800	*Ov-Rpl6*	2.640	*Ov-Tuba1a*	2.920

	*RefFinder*					
	Nervous		Allex		Adult	
	Gene name	Geomean of ranking values	Gene name	Geomean of ranking values	Gene name	Geomean of ranking values
1	*Ov-RNF7*	2.340	***Ov-Naa15***	2.170	***Ov-EIF2A***	1.970
2	***Ov-RIOK2***	2.550	***Ov-CUL1***	4.560	***Ov-CUL1***	4.420
3	*Ov-CHCHD7*	3.560	***Ov-EIF2A***	4.920	*Ov-RAD23B*	5.630
4	***Ov-slc25a40***	5.130	***Ov-RIOK2***	5.850	*Ov-Vbp1*	6.110
5	***Ov-UBE2F***	6.770	***Ov-slc25a40***	6.510	***Ov-syvn1***	6.320
6	*Ov-BTBD17*	7.140	***Ov-Ppm1b***	6.980	***Ov-Ppm1b***	6.500
7	*Ov-Abi2*	8.040	*Ov-KCMF1*	7.650	***Ov-UBE2F***	8.010
8	***Ov-Ppm1b***	8.710	*Ov-usp10*	7.650	***Ov-slc25a40***	9.260
9	***Ov-syvn1***	10.020	*Ov-EIF3M*	9.240	***Ov-Ltv1***	9.810
10	***Ov-Naa15***	10.580	***Ov-Ltv1***	9.390	*Ov-Rnd3*	10.260
11	*Ov-EIF3M*	12.350	*Ov-Rnd3*	12.330	*Ov-RIOK2*	11.470
12	*Ov-EIF2A*	12.420	*Ov-Dap3*	13.960	*Ov-usp10*	13.000
13	*Ov-RPS18*	15.200	*Ov-syvn1*	14.050	*Ov-gk5*	13.210
14	*Ov-CUL1*	15.740	*Ov-UBE2F*	14.460	*Ov-MRM2*	13.780
15	*Ov-Ltv1*	15.920	*Ov-RpL23*	15.600	*Ov-Dap3*	15.610
16	*Ov-usp10*	16.440	*Ov-ATPAF2*	16.460	*Ov-RNF7*	15.840
17	*Ov-AP5Z1*	18.110	*Ov-gk5*	16.790	*Ov-CHCHD7*	16.430
18	*Ov-Snx25*	19.580	*Ov-prrc1*	16.900	*Ov-EIF3M*	17.500
19	*Ov-PTPN12*	20.600	*Ov-RPS18*	17.150	*Ov-Abi2*	18.500
20	*Ov-MRM2*	21.160	*Ov-Vbp1*	17.390	*Ov-CPIJ005834*	18.830
21	*Ov-BTBD2*	21.160	*Ov-RAD23B*	17.820	*Ov-Fam160a2*	18.870
22	*Ov-Dnaja3*	22.350	*Ov-MRM2*	19.230	*Ov-Sdhd*	18.990
23	*Ov-Rnd3*	23.120	*Ov-MRPS5_F1R1*	20.170	*Ov-Naa15*	20.060
24	*Ov-CSDE1*	23.230	*Ov-CHCHD7*	20.220	*Ov-RPS18*	20.070
25	*Ov-Vbp1*	23.430	*Ov-CPIJ005834*	20.510	*Ov-ATPAF2*	22.590
26	*Ov-MRPS5_FR*	23.880	*Ov-MRPS5_FR*	23.270	*Ov-prrc1*	23.480
27	*Ov-Sdhd*	23.900	*Ov-RNF7*	23.770	*Ov-CG9286*	23.490
28	*Ov-RAD23B*	24.390	*Ov-Abi2*	24.500	*Ov-BTBD17*	24.110
29	*Ov-ATPAF2*	24.510	*Ov-CG9286*	25.190	*Ov-MRPS5_F1R1*	24.470
30	*Ov-KCMF1*	24.660	*Ov-BTBD17*	26.120	*Ov-MRPS5_FR*	27.340
31	*Ov-RpL23*	24.750	*Ov-Fam160a2*	26.450	*Ov-RpL23*	28.950
32	*Ov-Fam160a2*	26.300	*Ov-Sdhd*	26.650	*Ov-Abhd18*	29.040
33	*Ov-prrc1*	26.420	*Ov-mts*	31.030	*KCMF1*	29.500
34	*Ov-tollip*	30.000	*Ov-NOB1*	32.210	*Ov-UGP2*	29.830
35	*Ov-MRPS5_F1R1*	30.010	*Ov-Abhd18*	33.440	*Ov-rpf1*	29.920
36	*Ov-UGP2*	31.090	*Ov-PTPN12*	33.530	*Ov-CSDE1*	30.710
37	*Ov-CG9286*	31.990	*Ov-CSDE1*	33.740	*Ov-C2CD2*	31.240
38	*Ov-Dap3*	32.350	*Ov-C2CD2*	34.100	*Ov-timm*	32.860
39	*Ov-WBP2*	33.310	*Ov-SUCLG2*	34.490	*Ov-NOB1*	33.370
40	*Ov-CPIJ005834*	33.370	*Ov-rpf1*	35.440	*Ov-SUCLG2*	34.730
41	*Ov-gk5*	34.430	*Ov-UGP2*	35.770	*Ov-BTBD2*	38.610
42	*Ov-NOB1*	40.850	*Ov-tollip*	36.210	*Ov-PTPN12*	40.120
43	*Ov-Tuba1a*	41.290	*Ov-BTBD2*	38.930	*Ov-WBP2*	40.280
44	*Ov-TUBG1_FR*	41.490	*Ov-timm*	40.960	*Ov-mts*	43.380
45	*Ov-PCK1*	41.710	*Ov-Dnaja3*	42.420	*Ov-PCK1*	44.430
46	*Ov-mts*	42.380	*Ov-WBP2*	45.300	*Ov-AP5Z1*	44.510
47	*Ov-CSDE1*	45.520	*Ov-Snx25*	46.880	*Ov-tollip*	44.980
48	*Ov-rpf1*	45.730	*Ov-TUBG1_FR*	48.240	*Ov-AGL*	45.950
49	*Ov-timm*	46.230	*Ov-Tuba1a*	48.880	*Ov-Snx25*	47.140
50	*Ov-SUCLG2*	46.680	*Ov-AGL*	50.720	*Ov-Dnaja3*	47.720
51	*Ov-Abhd18*	48.230	*Ov-AP5Z1*	50.800	*Ov-PIP4K2B*	47.750
52	*Ov-AGL*	48.970	*Ov-PCK1*	51.830	*Ov-Snx20*	50.000
53	*Ov-Rpl6*	51.490	*Ov-Homer2*	52.170	*Ov-TUBG1_FR*	50.400
54	*Ov-TUBG1_F1R1*	53.430	*Ov-PIP4K2B*	52.240	*Ov-wdr44*	52.720
55	*Ov-Homer2*	53.490	*Ov-wdr44*	53.460	*Ov-Rps27a_FR*	53.820
56	*Ov-Snx20*	56.000	*Ov-Rps27a_FR*	53.820	*Ov-TUBG1_F1R1*	54.240
57	*Ov-PIP4K2B*	57.250	*Ov-TUBG1_F1R1*	54.710	*Ov-Homer2*	54.440
58	*Ov-wdr44*	58.250	*Ov-Snx20*	56.750	*Ov-Rpl6*	55.440
59	*Ov-Rps27a_FR*	58.490	*Ov-Rpl6*	57.710	*Ov-Tuba1a*	57.970

The results from the four approaches were integrated using *RefFinder*. The integration of the results was done calculating the geometric mean of the rank of each gene in all algorithms. In boldface common genes for the first 10 positions are highlighted for each algorithm. Integration of the different algorithms using the geometric mean of the rank of each gene is also provided. Stab. value, Stability value; Std. dev, Standard deviation; Geomean, geometric mean.

***geNorm analysis.*** *Ov-CHCHD7* and *Ov-RNF7* were identified as the two most correlated genes and therefore scored as the most stable RGs for the **Nervous** tissues (Table 2), followed by *Ov-RIOK2*, *Ov-UBE2F*, *Ov-slc25a40*, *Ov-BTBD17*, *Ov-Naa15*, *Ov-Ppm1b*, *Ov-CUL1*, *Ov-Snx25*, and *Ov-Dnaja3* (Table 2). Interestingly, the genes *Ov-RpL23*, *Ov-Rpl6*, *Ov-TuBg1-F1R1*, and *Ov-Rps27a-FR*, recently utilized as RGs in RT-qPCR experiments in cephalopods (Supplementary Table 2), were demonstrated to be among the most unstable genes (Table 2).

When additional tissues were considered (**Allex**), *Ov-CUL1* and *Ov-Naa15* were identified as the two most correlated genes, followed by *Ov-RIOK2*, *Ov-slc25a40*, *Ov-usp10*, *Ov-KCMF1*, *Ov-Ppm1b*, *Ov-EIF2A*, *Ov-EIF3M*, *Ov-CHCHD7,* and *Ov-syvn1*. The least stable genes included *Ov-Rpl6*, *Ov-Rps27a-FR*, *Ov-TUBG1-F1R1*, and *Ov-TUBG1-FR* (Table 2). In the analysis of all the tissues (**Adult**), *Ov-Vbp1, Ov-EIF2A*, *Ov-RAD23B*, *Ov-syvn1*, *Ov-CUL1*, *Ov-Ppm1b*, *Ov-CHCHD7*, *Ov-RIOK2*, *Ov-UBE2F,* and *Ov-RNF7* emerged as the most stable genes. Similarly to **Nervous** and **Allex**, *Ov-TUBG1-F1R1*, *Ov-TUBG1-FR*, *Ov-RpL23*, *Ov-Rpl6*, *Ov-Rps27a-FR,* and *Ov-Tuba1a* were the least stable genes. Among the 10 most stable genes, only *Ov-Ppm1b*, *Ov-CHCHD7*, and *Ov-CUL1* were shared by the three groups, while *Ov-RIOK2*, *Ov-slc25a40*, *Ov-Naa15*, *Ov-RNF7*, *Ov-UBE2F*, and *Ov-EIF2A* were shared between two groups (Table 2).

***NormFinder analysis.*** We identified *Ov-RIOK2*, *Ov-slc25a40*, *Ov-RNF7*, *Ov-CHCHD7*, *Ov-Ppm1b*, *Ov-syvn1*, *Ov-UBE2F*, *Ov-Naa15*, *Ov-BTBD17*, and *Ov-Abi2* as the most stable genes (**Nervous**, Table 2). For the **Allex** and **Adult** groups, some of the top genes in **Nervous** ranked at lower values (e.g., *Ov-RIOK2* and *Ov-slc25a40*), while others were considered more stable (e.g., *Ov-EIF2A* and *Ov-CUL1;* Table 2). *Ov-Naa15*, *Ov-EIF2A*, *Ov-CUL1*, and *Ov-UBE2F* were identified as stable reference genes in both groups, while *Ov-RIOK2*, *Ov-slc25a40*, *Ov-Ppm1b*, and *Ov-syvn1* were shared in all the considered tissue groups (Table 2).

***BestKeeper analysis.*** *Ov-Abi2*, *Ov-Ltv1*, and *Ov-gk5* were shown to be the most stable genes, which were shared between two groups, while *Ov-RPS18*, *Ov-RpL23*, *Ov-EIF3M*, and *Ov-BTBD17* were shared between the three groups (see Table 2). *Ov-RPS18* and *Ov-RpL23* were identified as suitable reference genes by this algorithm.

***Delta Ct method.*** *Ov-RIOK2*, *Ov-RNF7*, *Ov-slc25a40*, *Ov-CHCHD7*, *Ov-UBE2F*, *Ov-Ppm1b*, *Ov-Naa15*, *Ov-BTBD17*, *Ov-syvn1*, and *Ov-EIF2A* emerged as the most stable genes for the **Nervous** tissues (Table 2). However, several other genes showed a comparable standard deviation (Table 2). *Ov-Naa15*, *Ov-EIF2A*, *Ov-Ppm1b*, *Ov-CUL1*, *Ov-RIOK2*, *Ov-KCMF1*, *Ov-usp10*, *Ov-EIF3M*, *Ov-syvn1,* and *Ov-Vbp1* were selected as references for the **Allex** group. When all tissues were considered (**Adult**), *Ov-EIF2A*, *Ov-CUL1*, *Ov-Ppm1b*, *Ov-Vbp1*, *Ov-syvn1*, *Ov-slc25a40*, *Ov-UBE2F*, *Ov-RAD23B*, *Ov-Rnd3*, and *Ov-RIOK2* were identified as the most stable reference genes. *Ov-slc25a40*, *Ov-Naa15*, *Ov-Vbp1*, *Ov-CUL1,* and *Ov-UBE2F* were shared between two groups, while *Ov-Ppm1b*, *Ov-syvn1*, *Ov-RIOK,2* and *Ov-EIF2A* were shared between the three groups (Table 2).

### Comprehensive ranking of the reference genes

By comparing the 10 most stable genes identified by the four approaches in the same tissue group (**Nervous**), *Ov-RIOK2*, *Ov-UBE2F*, *Ov-slc25a40*, *Ov-Naa15*, and *Ov-Ppm1b* were identified as common in at least three algorithms, while *Ov-CHCHD7*, *Ov-RNF7*, and *Ov-BTBD17* resulted from all the four methods (Table 2). When arms but not tips were included as tissues (**Allex** group), *Ov-CUL1*, *Ov-Naa15*, *Ov-RIOK2*, *Ov-usp10*, *Ov-KCMF1*, *Ov-EIF2A*, and *Ov-Ppm1b* (Table 2) emerged as the best reference genes using the three approaches, while *Ov-EIF3M* was shared among the four methods (Table 2). The analysis performed considering the **Adult** tissues led to the identification of *Ov-RAD23B*, *Ov-EIF2A*, *Ov-Vbp1*, *Ov-syvn1*, *Ov-CUL1*, *Ov-Ppm1b*, and *Ov-UBE2F* as reference genes by the three approaches, but none of them were shared in all the considered methods.

The results from the four approaches were integrated using *RefFinder* (Xie et al., 2012). Overall, the top 10 most stable genes in the **Nervous** tissues were *Ov-RNF7*, *Ov-RIOK2*, *Ov-CHCHD7*, *Ov-slc25a40*, *Ov-UBE2F*, *Ov-BTBD17*, *Ov-Abi2*, *Ov-Ppm1b*, *Ov-syvn1*, and *Ov-Naa15* (Table 2). For **Allex**, the most stable genes were *Ov-Naa15*, *Ov-CUL1*, *Ov-EIF2A*, *Ov-RIOK2*, *Ov-slc25a40*, *Ov-Ppm1b*, *Ov-KCMF1*, *Ov-usp10*, *Ov-EIF3M*, and *Ov-Ltv1* (Table 2). When all tissues were considered (**Adults**), *Ov-EIF2A*, *Ov-CUL1*, *Ov-RAD23B*, *Ov-Vbp1*, *Ov-syvn1*, *Ov-Ppm1b*, *Ov-UBE2F*, *Ov-slc25a40*, *Ov-Ltv1*, and *Ov-Rnd3* were identified as reference genes, with *Ov-EIF2A* proving to be the best reference gene (see the geometric mean of the rank, Table 2). We also plotted the raw Ct for the best RGs identified (Supplementary Figure 4). The most stable genes shared by the combination of tissues were *Ov-Naa15* and *Ov-RIOK2* (**Nervous** and **Allex**); *Ov-CUL1*, *Ov-EIF2A,* and *Ov-Ltv1* (**Allex** and **Adult**); and *Ov-syvn1* and *Ov-UBE2F* (**Nervous** and **Adult**). Meanwhile, the *Ov-slc25a40* and *Ov-Ppm1b* results were shared among the three groups (Table 2).

### Reference genes validation

To investigate the reliability of the selected candidate RGs, the expression profiles of nine target genes (i.e., *Ov-Naa15*, *Ov-Ltv1*, *Ov-CG9286*, *Ov-EIF3M*, *Ov-NOB1*, *Ov-CSDE1*, *Ov-Abi2*, *Ov-Homer2*, and *Ov-Snx20*) were assessed in tissues belonging to the nervous system. The role of these genes (see Supplementary Info: Selected target genes for validation) is still unknown in cephalopods. The selection was based on their known functions in different organisms, particularly those related to their involvement in neuronal signaling, cytoskeleton functions, axon guidance, synaptogenesis, and behavioral plasticity.

This also allowed comparison of gene expression profiles among brain masses and peripheral ganglia (**Nervous**). The gastric ganglion (GG) was considered as the reference ‘tissue’. A combination of the two most stable (*Ov-RNF7* and *Ov-RIOK2*), the most stable (*Ov-RNF7*), and the least stable (most unstable; *Ov-Rps27a-FR*) RGs was used to normalize the expression of the target genes.

**Figure 2:**
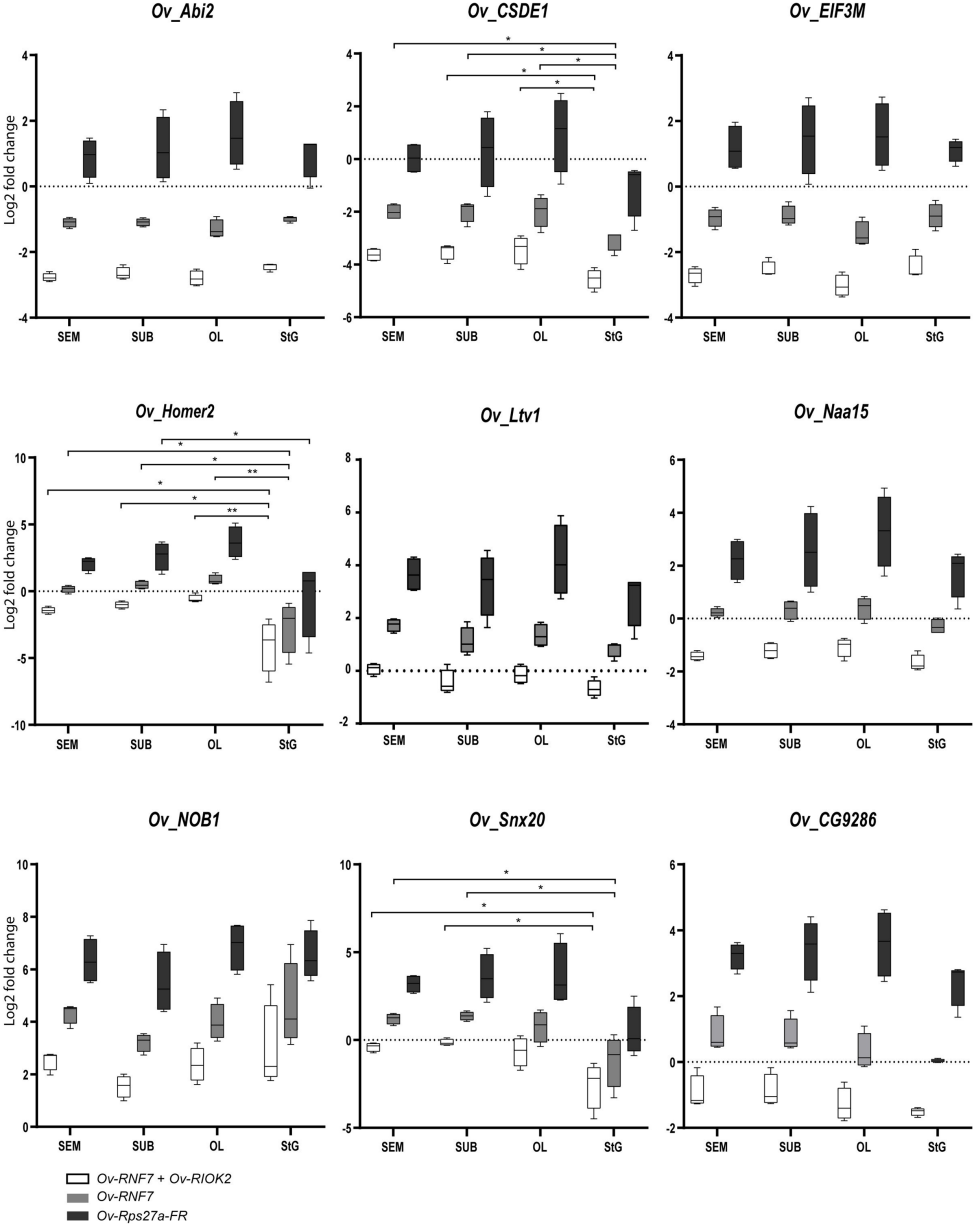
Relative expression levels of target genes: *Ov-Naa15*, *Ov-Ltv1*, *Ov-CG9286*, *Ov-EIF3M*, *Ov-NOB1*, *Ov-CSDE1*, *Ov-Abi2*, *Ov-Homer2*, and *Ov-Snx20* across all considered tissues (optic Lobe: OL; supraesophageal mass: SEM; suboesophageal mass: SUB; stellate ganglion: StG) normalized by the most stable reference gene combination (white bars), the most stable gene (grey), and the most unstable gene (black). Significant differences were assessed after ANOVA (*p* < 0.05, Bonferroni *post hoc*). See main text for details.

When the best RGs combination and the most stable gene were used for normalization of the expression of target genes in the nervous system of *O. vulgaris*, similar expression profiles were obtained for *Ov-CSDE1* and *Ov-Homer2* in the SEM, SUB, and OL, but the StG showed a significantly lower expression (Figure 2). A similar trend was also highlighted for *Ov-Snx20* (Figure 2) that showed a lower expression in the StG compared to the SEM and SUB. No significant differences resulted for the other target genes considered (Figure 2).

When the least stable reference gene *Ov-Rps27a-FR* was used for normalization, none of the nine genes investigated showed any significant change in expression except for *Ov-Homer2*, which appeared to be less expressed in the StG compared to the OL (Figure 2).

## Discussion

Exploring gene expression in the nervous system and other tissues helps to find molecular correlates of biological and neural plasticity, learning, and memory. In cephalopods, the study of the molecular machinery occurring in these processes is still limited. Few studies of cuttlefish (Agin et al., 2000, 2001, 2003; Focareta et al., 2014; Focareta and Cole, 2016; Bian et al., 2018), squid (Giuditta et al., 2002; Kimbell and McFall-Ngai, 2003; Burbach et al., 2019), and octopus (Zarrella, 2011; Zarrella et al., 2015; van Giesen et al., 2020; see also Prado-Álvarez et al., 2022) have been based on an exiguous number of specific candidate molecules involved in given functions or biological aspects of cephalopod plasticity.

Despite the availability of a few candidate RGs (review in Supplementary Table 2), the application of qRT-PCR in *O. vulgaris* also appears limited. Our approach was to expand the list of potential stable RGs in octopus through the use of the available transcriptomes (Petrosino, 2015; Petrosino et al., 2015, 2022), with the aim of facilitating a large-scale analysis of gene expression profiles under various conditions in different tissues.

We focused on genes that demonstrated stability and a uniform predicted expression within different tissues (peripheral and central nervous system and appendages). We explored their relative gene expression through qRT-PCR experiments using a subset of target genes. This approach allowed us to identify the most extensive set of stable reference genes currently available for the adult *O. vulgaris*.

Through *in silico* analysis of the octopus transcriptome, we found more than 2000 candidate RGs. However, we tested less than hundreds because of limitations in gene annotation. We identified a list of stable and uniformly expressed RGs across different body parts in adult individuals and in tissues including the nervous tissues (e.g., brain, gastric and stellate ganglia, and arm; Figure 1). The gene expression profiles of these potential RGs (n = 59) were assessed *via* qRT-PCR, and their stability was calculated and analyzed using different algorithms. The analysis of potential RGs in *O. vulgaris* revealed that there was no single reference gene that exhibited a constant expression level in all the samples, similarly to what has been reported in other organisms (e.g., Guo et al., 2014; Gao et al., 2017; Jin et al., 2019).

Via *RefFinder*, we identified RGs specific to the nervous system (**Nervous**, *Ov-RNF7*, and *Ov-RIOK2*), all tissues but the arm tips (**Allex**, *Ov-Naa15*, and *Ov-CUL1*), or those that are transcriptionally stable across all considered tissues (**Adult**, *Ov-EIF2A*, and *Ov-CUL1*, Table 2). In addition, *Ov-slc25a40* and *Ov-Ppm1b* were identified as shared best reference genes in the **Nervous, Allex,** and **Adult** groups of tissues (Table 2). Notably, the arm tips showed the highest variation in gene expression among the analyzed anatomical structures, likely due to the biological peculiarities of the octopus’ arm that maintains the ability of regeneration and indeterminate growth throughout adult ontogeny (Fossati et al., 2013, 2015; Nödl et al., 2015; Zullo et al., 2017; Tarazona et al., 2019; e.g., Zullo et al., 2019; van Giesen et al., 2020; see also De Sio and Imperadore, 2023).

The identified RGs are related to ubiquitination, rRNA processing, translation, and post-translational protein modifications, which are housekeeping functions in line with the typical references. Interestingly, none of them has ever been used as references in cephalopods before. Our approach—i.e., a large number of candidate transcripts and several tissues belonging to putatively different cell types (Styfhals et al., 2022)—provided more than 70 candidate RGs for *O. vulgaris.*

We also validated RGs by assessing the expression profiles of nine target genes (*Ov-Naa15*, *Ov-Ltv1*, *Ov-CG9286*, *Ov-EIF3M*, *Ov-NOB1*, *Ov-CSDE1*, *Ov-Abi2*, *Ov-Homer2*, and *Ov-Snx20*) in different tissues of the octopus nervous system. The expression after normalization by *Ov-RNF7* and *Ov-RIOK2* (the most stable RGs) differed from that of *Ov-Rps27a-FR* (the least stable gene), which is commonly used as an RG for data normalization (Figure 2).

In conclusion, we utilized different algorithms to evaluate the expression profiles of tens of candidate RGs of *O. vulgaris*. We identified those that can be used in the normalization of the qRT-PCR data and suggested RGs that can be used cautiously with different tissue groups.

Our findings will aid future investigations of the transcriptional landscape of cephalopods and facilitate the study of the molecular basis of neural plasticity and other phenomena.

## Data availability statement

The original contributions presented in the study are included in the article/Supplementary material, further inquiries can be directed to the corresponding author.

## Ethics statement

Ethical review and approval was not required for the animal study because killing animals solely for tissue removal does not require authorization from the National Competent Authority under Directive 2010/63/EU (European Parliament & Council of the European Union, 2010) and its transposition into National legislation. Sampling of octopuses from artisanal fishermen included in this study was authorized by the local Animal Welfare Body (Ethical Clearance: case 5/2021/ec AWB-SZN).

## Author contributions

PI and SC performed the experiments, data curation, and analysis and contributed to the study conceptualization. VA and CM contributed to the experiments and data curation. PI, SC, and GF contributed to writing the original draft. GF and GP contributed to the study conceptualization, investigation, writing, and funding acquisition. All authors contributed to the article and approved the submitted version.

## Funding

This work was supported by the Stazione Zoologica Anton Dohrn intramural research fund granted to GF and GP and by a HSA-Ceph 1/2019 grant to GP. VA was supported by a short-term fellowship from the Stazione Zoologica Anton Dohrn.

## Conflict of interest

The authors declare that the research was conducted in the absence of any commercial or financial relationships that could be identified as a potential conflict of interest.

## Publisher’s note

All claims expressed in this article are solely those of the authors and do not necessarily represent those of their affiliated organizations, or those of the publisher, the editors and the reviewers. Any product that may be evaluated in this article, or claim that may be made by its manufacturer, is not guaranteed or endorsed by the publisher.

## Footnotes

^1^ https://www.thermofisher.com/it/en/home/brands/thermo-scientific/molecular-biology/molecular-biology-learning-center/molecular-biology-resource-library/thermo-scientific-web-tools/multiple-primer-analyzer.html
