# Supplementary material for: Transcriptome-wide selection and validation of a solid set of reference genes for gene expression studies in the cephalopod mollusk Octopus vulgaris
Source: Front Mol Neurosci. 2023 May 17;16:1091305. doi: 10.3389/fnmol.2023.1091305 (PMC10230085; doi:10.3389/fnmol.2023.1091305)
Supplement: Supplementary file 1 [file Data_Sheet_1.pdf]

Supplementary Information to

Transcriptome-wide selection and validation of a solid set of reference genes for gene expression studies in the cephalopod mollusc *Octopus vulgaris*

Pamela Imperadore[1,*], Stefano Cagnin[2,3,*], Vittoria Allegretti[1], Caterina Millino[2], Francesca Raffini[1], Graziano Fiorito[1], Giovanna Ponte[1]

[1] Department of Biology and Evolution of Marine Organisms, Stazione Zoologica Anton Dohrn, Napoli, Italy

[2] Department of Biology, University of Padova, Padova, Italy

[3] CIR-Myo Myology Center, University of Padova, Padova, Italy

* These authors contributed equally to the work

Corresponding Author:
Dr Pamela Imperadore
Email: imperadore.p@gmail.com
pamela.imperadore@szn.it

Key Words: *Octopus vulgaris*; cephalopod molluscs; reference genes; RT-qPCR; molecular fingerprint; nervous system.

Table of Contents

Cephalopod genomic resources 3

Reference genes for cephalopods: previously published data 5

In silico gene expression levels for candidate reference genes for *O. vulgaris* 10

Supporting Data and Methods 11

- Animals and Samples 11
- RNA extraction and cDNA synthesis 12
- Primers' amplification specificity and efficiency 15
- Supplementary Table 4 – List of primers 18

Amplification of the candidate reference genes 32

Ct values of the candidate reference genes for *O. vulgaris* 33

Validation of the *in silico* gene expression profiles 39

Selected target genes for validation 39

References 42

Cephalopod genomic resources

A large set of genomic and transcriptomic data have been generated over the last decade for several cephalopod species thanks to the effort originated from various important initiatives including a series of meetings organized in Europe and the United States of America (Albertin et al., 2012; Ponte et al., 2013).

NCBI provides access to genomes available (https://www.ncbi.nlm.nih.gov/data-hub/genome/?taxon=6605) and counts over 200,000 hits as mRNA sequences (total number of hits belonging to cephalopods in GenBank: 147,354; in RefSeq: 60,096). In addition, over 90-thousand nucleotide sequences and more than 100-thousand ESTs are available (source: https://www.ncbi.nlm.nih.gov/nuccore; last visited: October 2022).

Here we provide a short list of published works that are based on these data (Supplementary Table 1) and represent the main genomic and transcriptomic resources currently available for these organisms. The overall picture emerging is the complexity and richness of cephalopods' physiological and morphological adaptations, a long list of novelties mediated by genome reorganization, gene families expansion, tissue-dependent mRNA editing, to mention some (e.g., Ritschard et al., 2019; Albertin and Simakov, 2020; Albertin et al., 2022; Schmidbaur et al., 2022).

Supplementary Table 1. List of genomes and transcriptomes available for cephalopods and reference to the published works where these resources are presented and/or discussed. Species are arranged alphabetically.

Species	Genomes	References	Transcriptomes	References
Architeuthis dux	✓	da Fonseca et al. (2020)		
Argonauta argo	✓	Yoshida et al. (2022)		
Doryteuthis (Loligo) pealeii	✓	Albertin et al. (2022)		
Euprymna albatrossae	✓	Heath-Heckman and Nishiguchi (2021)		
Euprymna hylleberg	✓	Heath-Heckman and Nishiguchi (2021)		
Euprymna scolopes	✓	Belcaid et al. (2019)	✓	Collins et al. (2012); Pankey et al. (2014)
Euprymna tasmanica			✓	Salazar et al. (2015)
Idiosepius paradoxus			✓	Sousounis et al. (2013)
Nautilus pompilius	✓	Zhang et al. (2021)	✓	Sousounis et al. (2013)
Octopus bimaculoides	✓	Albertin et al. (2015)	✓	Albertin et al. (2015)
Octopus maya			✓	
Octopus minor	✓	Kim et al. (2018)	✓	Kim et al. (2018)
Octopus sinensis	✓	Li et al. (2020)	✓	Sun et al. (2022)
Octopus vulgaris	✓	Zarrella et al. (2019)	✓	Zhang et al. (2012); Castellanos-Martínez et al. (2014); Petrosino (2015); Petrosino et al. (2022); García-Fernández et al. (2019); Prado-Álvarez et al. (2022)
Rondeletiola minor	✓	Heath-Heckman and Nishiguchi (2021)		
Sepia esculenta			✓	Zhang et al. (2019)
Sepia officinalis			✓	Benoist et al. (2020)
Sepia pharaonic	✓	Song et al. (2021)		
Sepiadarium austrinum			✓	Caruana et al. (2016)
Sepiella japonica			✓	Lü et al. (2016)
Sepiella maindroni			✓	Tian et al. (2018)
Sepietta neglecta	✓	Heath-Heckman and Nishiguchi (2021)		

Reference genes for cephalopods: previously published data

Supplementary Table 2. Overview of previous methods and main results from papers published to identify reference genes in cephalopod molluscs. For each paper we include: species, number of animals, tissues tested, Reference Genes (RGs), a summary of experimental conditions included in the work, the best reference genes resulted as outcome of the study based on the algorithm utilized [*BestKeeper* (Pfaffl et al., 2004), *geNorm* (Vandesompele et al., 2002; Mestdagh et al., 2009), *NormFinder* (Andersen et al., 2004), *RefFinder* (Xie et al., 2012)], a short overview of the main results of the study, and references.

Species	Number of Animals (N)	Tissues	RGs tested	Condition	Best reference genes (based on different algorithms)				Overall results	Reference
					BestKeeper	geNorm	NormFinder	RefFinder		
Octopus vulgaris	25	Mantle (without the skin)<br>SEM<br>SUB<br>OL	a-Tubulin (*tubA*)<br>b-actin (*actB*)<br>elongation factor 1A (*EEF1A*)<br>Ubiquitin/S27a (*ubi*)<br>16S rRNA (*16S*)<br>18S rRNA (*18S*)	**Condition 1:** *Octopus vulgaris* (30-2100g) collected throughout the year 2006	*ubi*	**N/A**	*ubi*		*NormFinder*: ubi and tubA as the best combination of reference genes for Conditions 1 and 2, when the mantle is used as control tissue for the study of gene expression in the brain.	Sirakov et al. (2009)
				**Condition 2:** *Octopus vulgaris* (200-550g) collected in June – July, 2006	*tubA-ubi*	*tubA-ubi*	*ubi*			
				**Condition 1 and 2**	*tubA*	**N/A**	*ubi*			

Species	Number of Animals (N)	Tissues	RGs tested	Condition	Best reference genes (based on different algorithms)				Overall results	Reference
					**BestKeeper**	**geNorm**	**NormFinder**	**RefFinder**		
Octopus vulgaris	NA	embryos, paralarvae	b-actin<br>EF1-a<br>Ubiquitin<br>a-Tubulin<br>GADPH<br>18S	**Condition 1:** *Octopus vulgaris* embryo and paralarvae (0, 10, 15, 20, 30, 34 days post hatching) non-infected	*TUB > UBI > EF1-a*	*UBI + EF1-a > 18S > TUB > GADPH > ACT*	*UBI > 18S > ACT*		*geNorm* and *NormFinder* identified as best refence genes:<br>*UBI*, *18S* and *EF1-a* for **Condition 1**; *UBI*, *ACT* and *18S* **Condition 2**. *BestKeeper* results support those genes.<br><br>The number of candidate genes required for normalization for both conditions = 5, based on the pairwise number variation analysis (Vn/n+1). Authors suggest the use of three reference genes enough for the normalization procedure.	García-Fernández et al. (2016)
		paralarvae collected at 1 h, 4 h and 24 h post-exposure		**Condition 2:** *Octopus vulgaris* paralarvae (20 days post hatching) exposed to *V. lentus* or *V. splendidus).*	*TUB > UBI > ACT*	*ACT + 18S > UBI > EF1-a> TUB > GADPH*	*UBI > 18S > ACT*			

Species	Number of Animals (N)	Tissues	RGs tested	Condition	Best reference genes (based on different algorithms)				Overall results	Reference
					**BestKeeper**	**geNorm**	**NormFinder**	**RefFinder**		
Octopus minor	20	brain<br>gill<br>digestive gland<br>hemocytes[1]	Elongation factor 1-alpha (*EF-1α*)<br>g-tubulin (*TUB*)<br>β-actin (*ACT*)<br>40S Ribosomal protein s18 b (*RPS18b*)<br>60S ribosomal protein L29 (*RPL29*)<br>28S ribosomal protein S5 (*RPS5*)<br>60S ribosomal protein L6 (*RPL6*)<br>Ornithine decarboxylase (*OD*)<br>Annexin A4 (*AA4*)	**Condition 1:** *Octopus minor* (86 g to 214 g) treated with 48-h half-lethal dose of ammonia for 8h<br>**Condition 2:** *Octopus minor* (86 g to 214 g) acclimatized in a normal culturing condition and defined as the control set	gills: *RPL6*, *RPS5* and *RPL29*.<br>hemolymph: *EF-1α*, *RPS18b* and *RPL29*	gill and digestive gland: *RPL29* and *RPL6*<br>brain: *TUB* and *RPL6*<br>hemolymph *EF-1α* and *RPS18b*	brain and gill: *RPS5*<br>digestive gland: *RPL6*<br>hemolymph: *EF-1α*	gill, digestive gland and brain: *RPL6*<br>hemolymph: *EF-1α*	Normalization evaluated on *geNorm* through pairwise variation analysis (Vn/n+1).<br>To normalize the expression of target genes it is recommended the use of:<br>- the combination of two reference genes in gill (*RPS5* and *RPL6*) and hemolymph (*EF-1α* and *RPS18b*)<br>- the combination of four reference genes in digestive gland (*RPL6*, *TUB*, *RPL29* and *OD*)<br>- the combination of three reference genes in brain (*EF-1α*, *TUB* and *RPL6*)	Xu and Zheng (2018)

[1] In the work the RNA is extracted from hemocytes, but in the paper results are reported as from hemolymph.

Species	Number of Animals (N)	Tissues	RGs tested	Condition	Best reference genes (based on different algorithms)				Overall results	Reference
					BestKeeper	geNorm	NormFinder	RefFinder		
Octopus minor	16	blood (~1 mL/octopus)<br>brain<br>gill<br>testis/ovary<br>intestine<br>digestive gland<br>muscle<br>systemic heart	Elongation factor 1-beta (*EF-1β*)<br>Elongation factor 2 (*EF-2*)<br>Ribosomal protein L13 (*Rpl13*)<br>TATA-binding proteinassociated factor 172 (*TBA*)<br>Histone H4 (*H4*)<br>Elongation factor 1-gamma (*EF-1γ*)<br>Succinate-hydroxymethylglutarate CoA-transferase (*SUGCT*)<br>Transferrin (*TF*)	**Condition 1:** *Octopus minor* (~120 g) exposed at **20°C** for 0, 6, 24, 72 h.<br>Condition 2: Octopus minor (~120 g) exposed at **24°C** for 0, 6, 24, 72 h		*H4*, *TF*, *TBA*, and *SUGCT*			*TF* gene is defined as the best reference gene in this study based on *geNorm* analysis. The optimal number of reference genes required for normalization, calculated using pairwise variation analysis (Vn / Vn + 1), is not conclusive as there are no V values below the cut-off value of 0.15.	Whang et al. (2020)

Species	Number of Animals (N)	Tissues	RGs tested	Condition	Best reference genes (based on different algorithms)				Overall results	Reference
					**BestKeeper**	**geNorm**	**NormFinder**	**RefFinder**		
Octopus vulgaris	10	Gastric ganglia	eukaryotic translation initiation factor 4 (*EIF4G1*)<br>LIM and SH3 domain protein (*F42H10.3*)<br>lamin-B1 (*Lmnb1*)<br>cytoplasmic FMR1 (*Sra-1*)<br>ubiquitin-40S ribosomal protein S27a (*RPS27A*)<br>elongation factor 1-alpha (*eef1a*)<br>40S ribosomal protein S18 (*RPS18*)	*Octopus vulgaris* naturally infected by *Aggregata octopiana*	*Lmnb1*, *Sra-1* and *RPS27A*		*Lmnb1*, *Sra-1* and *RPS27A*		Gene expression stability of the candidate reference genes evaluated with *BestKeeper* and *NormFinder* allowed for the identification of the three most stable reference genes: i.e., *Lmnb1*, *Sra-1* and *RPS27A*.	Baldascino et al. (2017)

In silico gene expression levels for candidate reference genes for *O. vulgaris*

transcript ID	Group	Gene name
c17784_g1_i1	**Nervous System**	*Ov-Klhdc*
c28856_g1_i2	**Nervous System**	*Ov-CUL1*
c30400_g11_i1	**Nervous System**	*Ov-wdr44*
c30725_g11_i1	**Nervous System**	*Ov-timm*
c31227_g1_i2	**Nervous System**	*Ov-tollip*
c31322_g1_i1	**Nervous System**	*Ov-prrc1*
c31554_g1_i3	**Nervous System**	*Ov-gk5*
c32096_g14_i2	**Nervous System**	*Ov-Canx*
c33604_g6_i1	**Nervous System**	*Ov-CHCHD7*
c33913_g6_i1	**Nervous System**	*Ov-PTPN12*
c35499_g5_i1	**Nervous System**	*Ov-ube2c*
c35786_g9_i1	**Nervous System**	*Ov-Naa15*
c36083_g5_i1	**Nervous System**	*Ov-SUCLG2*
c25466_g1_i1	**ADULT**	*Ov-Ltv1*
c29044_g1_i1	**ADULT**	*Ov-rpf1*
c29430_g1_i1	**ADULT**	*Ov-CG9286*
c29524_g1_i1	**ADULT**	*Ov-EIF3M*
c30066_g9_i1	**ADULT**	*Ov-NOB1*
c31610_g1_i1	**ADULT**	*Ov-slc25a40*
c32170_g13_i2	**ADULT**	*Ov-Dap3*
c32751_g1_i1	**ADULT**	*Ov-flr*
c33222_g7_i1	**ADULT**	*Ov-RIOK2*
c34059_g14_i1	**ADULT**	*Ov-ATPAF2*
c34087_g16_i1	**ADULT/BRAIN**	*Ov-CSDE1*
c34313_g4_i1	**ADULT**	*Ov-Ppm1b*
c34776_g5_i1	**ADULT**	*Ov-usp10*
c35010_g2_i4	**ADULT**	*Ov-EIF2A*
c35032_g7_i2	**ADULT**	*Ov-Dnaja3*
c35311_g1_i1	**ADULT**	*Ov-CPIJ005834*
c35707_g2_i1	**ADULT/Nervous system**	*Ov-PRMT5*
c36175_g1_i1	**ADULT**	*Ov-BTBD17*
c26803_g1_i1	**ARM**	*Ov-RAD23B*
c28702_g2_i1	**ARM**	*Ov-Abhd18*
c28934_g1_i1	**ARM**	*Ov-UGP2*
c29941_g6_i1	**ARM**	*Ov-14-3-3zeta*
c30691_g3_i1	**ARM**	*Ov-RSU1*
c31105_g4_i1	**ARM**	*Ov-BTBD2*
c32222_g5_i1	**ARM/ADULT/Nervous system**	*Ov-UBE2F*
c32350_g3_i1	**ARM**	*Ov-nAChRalpha1*
c32876_g12_i1	**ARM**	*Ov-RNF7*
c32876_g7_i5	**ARM**	*Ov-KCMF1*
c33117_g3_i1	**ARM**	*Ov-Rnd3*
c33305_g9_i1	**ARM**	*Ov-Abi2*
c34295_g8_i1	**ARM**	*Ov-Vbp1*
c34563_g2_i1	**ARM**	*Ov-PCK1*
c35194_g4_i1	**ARM**	*Ov-C2CD2*
c35789_g7_i1	**ARM**	*Ov-MRM2*
c36050_g13_i1	**ARM**	*Ov-Sdhd*
c28771_g3_i1	**BRAIN**	*Ov-AP5Z1*
c29565_g1_i1	**BRAIN**	*Ov-CERK*
c30947_g6_i1	**BRAIN**	*Ov-syvn1*
c31295_g14_i1	**BRAIN**	*Ov-AP1M1*
c32955_g4_i1	**BRAIN**	*Ov-Homer2*
c34071_g2_i1	**BRAIN/Nervous System**	*Ov-mts*
c34695_g13_i5	**BRAIN**	*Ov-CNBP*
c34716_g8_i1	**BRAIN**	*Ov-USP15*
c34844_g11_i1	**BRAIN**	*Ov-WBP2*
c34932_g8_i1	**BRAIN/Nervous System**	*Ov-MTX1*
c35016_g13_i1	**BRAIN/Nervous System**	*Ov-Gsk3b*
c35037_g6_i2	**BRAIN**	*Ov-PIP4K2B*
c35327_g8_i2	**BRAIN**	*Ov-FBXO38*
c35361_g5_i1	**BRAIN**	*Ov-Fam160a2*
c35373_g3_i2	**BRAIN**	*Ov-Snx20*
c35771_g14_i2	**BRAIN/Nervous System**	*Ov-Gnaq*
c35896_g5_i1	**BRAIN**	*Ov-Snx25*
c36137_g10_i4	**BRAIN**	*Ov-AGL*
c30165_g11_i1	**BRAIN**	*Ov-wls*
c34939_g11_i1	**ARM**	*Ov-ESR16*
c35194_g4_i2	**ARM**	*Ov-C2CD2*
**Previously published**		
**c5816_g1_i1**	**Previously published**	*Ov-Rps27a*
**c26807_g1_i1**	**Previously published**	*Ov-eef1a*
**c12855_g1_i1**	**Previously published**	*Ov-TUBG1*
**c34110_g1_i1**	**Previously published**	*Ov-MRPS5*
**c36025_g3_i2**	**Previously published**	*Ov-Tuba1a*
**c2281_g1_i1**	**Previously published**	*Ov-Rpl6*
**c30772_g3_i11**	**Previously published**	*Ov-RpL23*
**c29373_g3_i1**	**Previously published**	*Ov-RPS18*

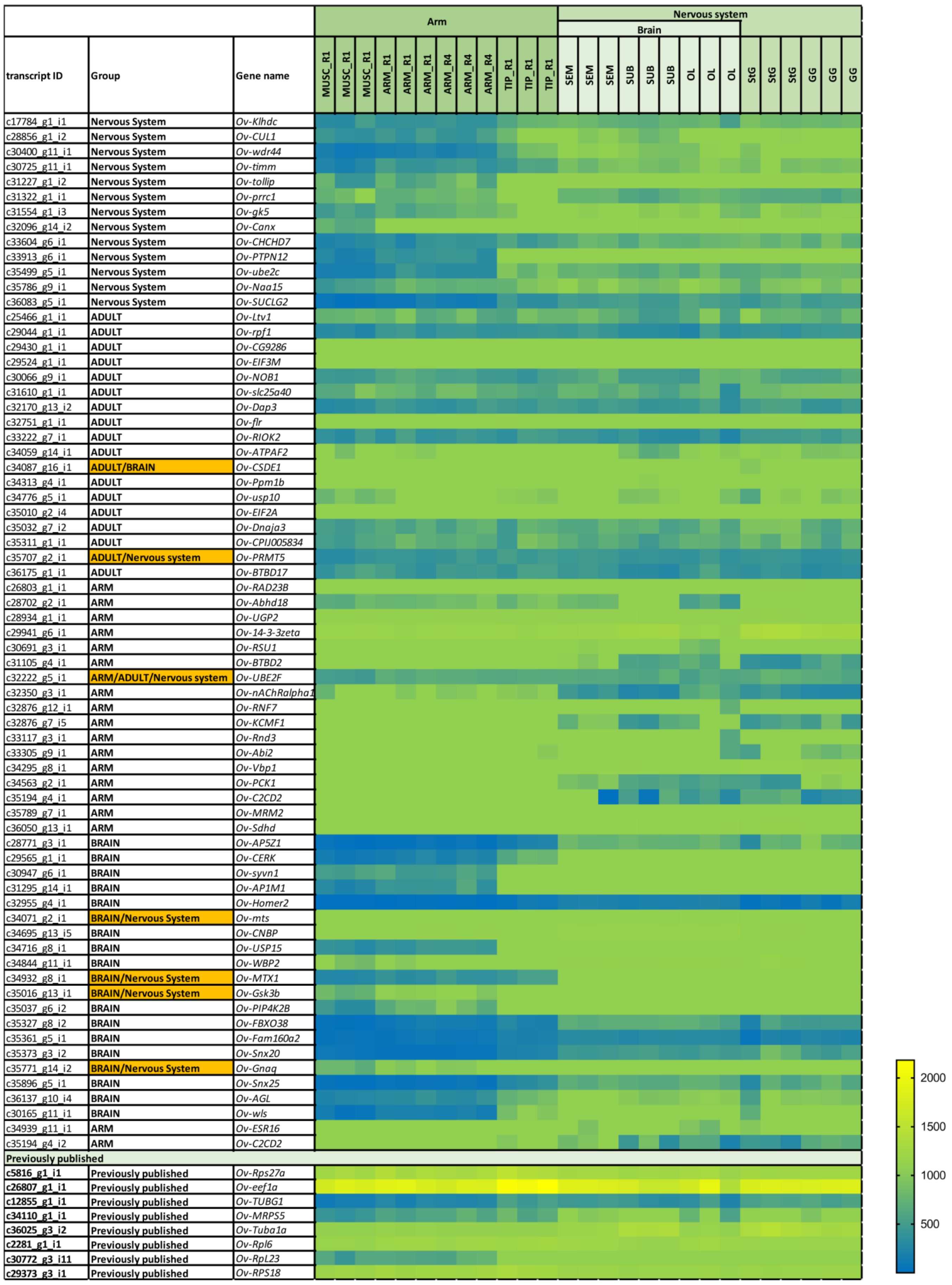

Supplementary Figure 1. Heatmap of expression levels of candidate reference genes selected from the transcriptome of *Octopus vulgaris* (Petrosino, 2015; Petrosino et al., 2022). Expression levels, based on RNA-seq data, are coded according to Row Z-score (see coloured bar) and included for octopus' brain (supra-, sub-oesophageal masses, and optic lobes), gastric- and stellate ganglia, and the arm (proximal parts: anterior and posterior arm (first and fourth, respectively; distal part: arm tip), and arm muscle only. Each transcript is uniquely identified and tissue grouping is included (see text for details).

Supporting Data and Methods

Animals and Samples

Octopus vulgaris were collected between June 11th and July 10st 2020 by local artisanal fishermen in the Bay of Naples (Italy). Animals were sexed and weighed (Supplementary Table 3) and humanely killed adopting the principles described in Annex IV of Directive 2010/63/EU and following Andrews et al. (2013), Fiorito et al. (2015) and Butler-Struben et al. (2018). In brief, octopuses were immersed in freshly made 3.5% magnesium chloride hexahydrate (Sigma Aldrich, CAS Number: 7791-18-6) dissolved in seawater; after around 30 min, animals were unresponsive to handling and noxious mechanical stimulus (e.g., forceps pinch on skin). In agreement to what reported by Grimaldi et al. (2007), ventilation stopped around ten minutes after immersion in the solution. No signs of Typical Aversive Response Patters were observed (Gleadall, 2013). Death was confirmed by transecting the dorsal aorta.

Following the Directive 2010/63/EU and its transposition into national legislation that regulates the use of live animals for scientific purposes, killing animals solely for tissue removal does not require authorization from the National Competent Authority. In addition, sampling of octopuses for this study originates from animals caught by artisanal fishermen for human consumption. This study was authorized by the local Animal Welfare Body (Ethical Clearance case 5/2021/ec AWB-SZN).

Tissues for biological validation were harvested from five *O. vulgaris* specimens that did not show any signs of lesions or aberrant formations or regenerating parts.

At sacrifice, 12 tissues were harvested from each octopus including a range of diverse structures belonging to the central and peripheral nervous tissues, arms, muscles and other organs (see Figure 1), namely: SEM, SUB, OL, GG, StG, the posterior left gill (GILL), a portion (around 1 cm^2) of muscle from the ventral side of the mantle (MANT) after skin removal, the most distal part (2 cm in length) of the first anterior right arm (Tip_R1) and of the fourth posterior right arm (Tip_R4); a piece of these two arms (2 cm in length) taken at around 50% of their length (ARM_R1 and ARM_R4); a piece (1cm in length) of muscle from arm R1 (MUSC_R1).

Supplementary Table 3. Animals, sampling date and main morphological characteristics: body weight (BW), dorsal (DML) and ventral (VML) mantle length, mantle (DMW) and head width (HW). Sex was assessed following Chapko et al. (1962) and Guerra (2019). Euthanasia (deep and prolonged anaesthesia by immersion in 3.5% solution of $MgCl_2$ in seawater) was accomplished following Grimaldi et al. (2007) and Butler-Struben et al. (2018); see text for details.

Animal Id	Sampling Date	BW (g)	DML (mm)	DMW (mm)	HW (mm)	VML (mm)	SEX
20 01	11/06/2020	256	89.0	74.0	34.2	65.4	Female
20 02	11/06/2020	348	94.1	75.9	36.6	63.8	Male
20 03	17/06/2020	1274	170.0	109.0	61.5	108.1	Male
20 04	01/07/2020	232	100.4	70.0	37.6	66.0	Male
20 05	10/07/2020	1200	157.0	101.8	54.0	100.5	Female

The collected tissues were weighted, immersed in 1 mL TRIzol® Reagent (Invitrogen, 12183018A) per 50-100 mg of sample and stored at -80°C until further processing.

The identified candidate genes were validated through PCR reactions.

RNA extraction and cDNA synthesis

Tissues were homogenized on ice, using the Ika Werke T10 homogenizer (IKA). After incubation (5 min at room temperature) to allow complete dissociation of proteins from nucleic acids, 200 µl of chloroform were added per ml of Trizol, mixed, and incubated on ice for 15 min. Samples were centrifuged (12.000 x g, 4° C) and the upper aqueous phase was transferred to a silica-cartridge for the RNA purification (PureLink® RNA Mini Kit; Cat No-12183018A, Invitrogen). Potential contaminating genomic DNA was degraded using the TURBO DNA-free™ Kit (Cat No-AM1907, Invitrogen) following the manufacturer's instructions. In brief: 5 µL of 10X TURBO DNAse Buffer and 1 µL TURBO DNAse (2U) were added in the 50 µL RNA sample and incubated at 37°C for 20 minutes. DNAse Inactivation Reagent (5 µL) was then added to the reaction tube, incubating for 5 min at room temperature, mixing occasionally. The tube was centrifuged (10,000 × g, 1.5 min) and the supernatant RNA was transferred to a fresh tube.

Quality and quantity of extracted RNA was assessed through UV absorption measurements (Nanodrop ND-1000 UV-Vis spectrophotometer, Nanodrop Technologies). All samples

showed an OD260/OD280 ratio between 1.8 and 2.0 and an OD260/OD230 ratio higher than 1.7. Absence of DNA contamination was verified through PCR followed by gel electrophoresis. RNA integrity was tested using the Agilent Bioanalyzer 2100 (Agilent Technologies). All samples showed no signs of degradation or evidenced any DNA contamination (Supplementary Figure 2). It is noteworthy to report that *O. vulgaris* has rRNAs running at about 45 and 43 seconds, corresponding to about 2,550 and 1,850 nucleotides, respectively (Supplementary Figure 2). The detected size is like mouse and human for 18S rRNA (1.9 Kb), but very different from 28S (mouse 4.7 and human 5 Kb). Due to this peculiar run, it is impossible to calculate the RNA Integrity Number (RIN), as recently reported for mollusks and cephalopods (Natsidis et al., 2019; Adema, 2021; Prado-Álvarez et al., 2022).
The High-Capacity cDNA Reverse Transcription Kit (Cat No-4368814, Applied Biosystems) was used to retrotranscribe 1.5 µg of RNA for each sample, following the manufacturer protocol. cDNA samples (20 µl of reaction volume) were stored at -20°C until use.

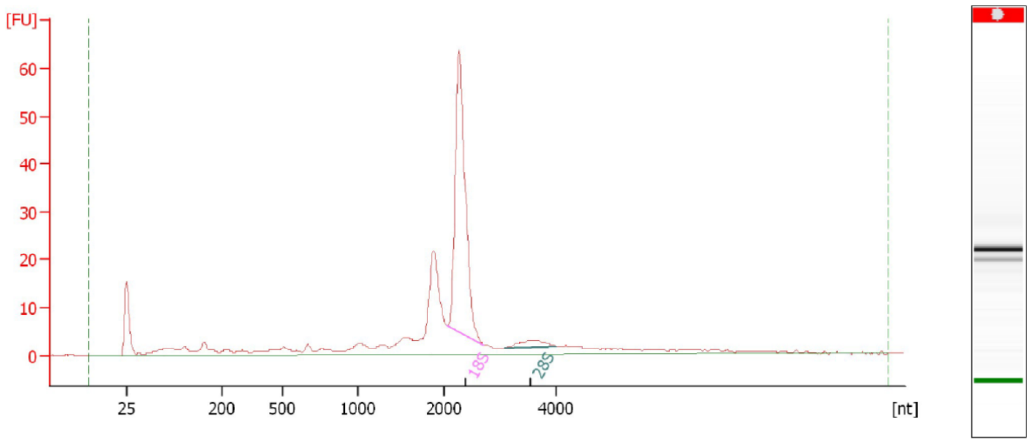

Overall Results for sample 7 :

RNA Area:	277.5	RNA Integrity Number (RIN):	N/A (B.02.08)
RNA Concentration:	177 ng/µl	Result Flagging Color:	
rRNA Ratio [28s / 18s]:	0.1	Result Flagging Label:	RIN N/A

Fragment table for sample 7 :

Name	Start Size [nt]	End Size [nt]	Area	% of total Area
18S	2,053	2,704	99.8	36.0
28S	3,080	3,992	5.3	1.9

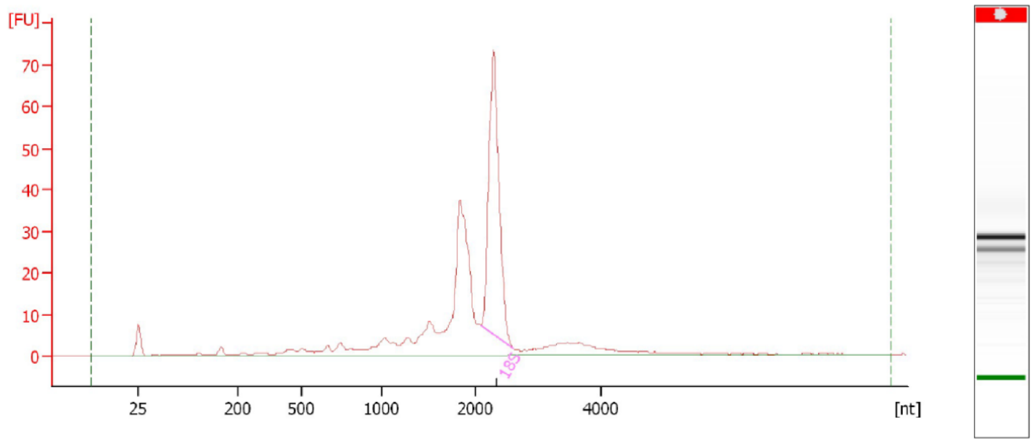

Overall Results for sample 6 :

RNA Area:	341.2	RNA Integrity Number (RIN):	N/A (B.02.08)
RNA Concentration:	191 ng/µl	Result Flagging Color:	
rRNA Ratio [28s / 18s]:	0.0	Result Flagging Label:	RIN N/A

Fragment table for sample 6 :

Name	Start Size [nt]	End Size [nt]	Area	% of total Area
18S	2,083	2,614	110.6	32.4

Supplementary Figure 2. Examples of the profiles generated on the Agilent 2100 Bioanalyzer including information on concentration, inspection of RNA integrity, and ribosomal ratios. No degradation was observed in any of the RNA samples obtaining an appropriate quality for subsequent analysis. See text for details.

Primers' amplification specificity and efficiency

Primers for the candidate reference genes (RGs; Supplementary Table 4) were designed on the basis of the sequences retrieved from the previous RNA-seq experiments, using Primer3 Plus software (Untergasser et al., 2012). In addition to these *de novo* designed primers, eight additional primer couples from RGs utilized in previous qRT-PCR experiments were selected from literature and slightly modified, if needed, to match *O. vulgaris* sequence (Supplementary Table 4).

Primers' specificity and efficiency was assessed by PCR and qRT-PCR.

For the standard PCRs, we used a thermal cycler (SimpliAmp Thermal Cycler, Thermo Scientific) using the following amplification mix: 25 µl of DreamTaq PCR Master Mix (2X; Cat No-K1071, Thermo Scientific) mixed with 0.5 µM of forward and reverse primers respectively (1 µl each) and 1 µl of cDNA. A final reaction volume of 50 µl was reached through the addition of nuclease-free water. The initial PCR cycle included 2 min at 95°C, followed by 35 cycles of 30 s at 95°C, 30 s at 60°C, and 1 min at 72 °C, then, a final extension of 10 min at 72°C. To confirm the presence of a single band in the PCR products, the amplification products were visualized on 2% agarose (Cat No-1127, Agarose Low EEO, BioFroxx) gel electrophoresis (Supplementary Figure 3). Both, gel electrophoresis after PCR and dissociation curve analyses, evidenced that all primers investigated, but three, allow for the amplification of specific genes without spurious by-products. Primers designed for *Ov-wls*, *Ov-ESR16* and *Ov-C2CD2* (isoform X2) were excluded from this study as none, or multiple amplification products were detected. Supplementary Table 4 shows amplicon size, Tm and other relevant details for each amplicon (see also Supplementary Figure 2).

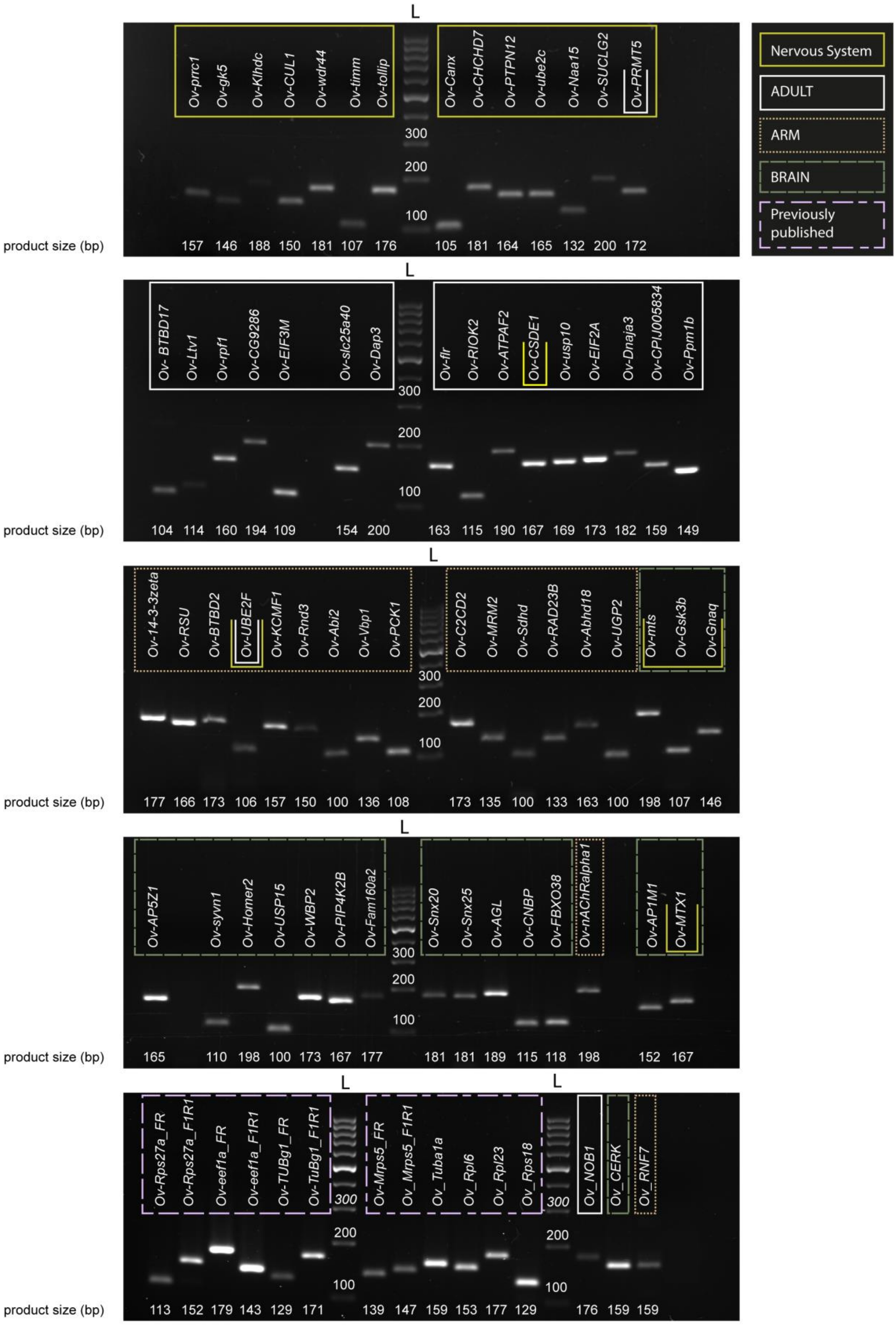

Supplementary Figure 3. Primer couples tested for PCR and visualized on 2% agarose gel electrophoresis presenting a single amplification product at the expected amplicon length.

For qRT-PCR we used 5x HOT FIREPol® EvaGreen® qPCR Mix Plus (ROX) (Cat No-08-24-, Solys BioDyne) on the CFX 384 Real-Time PCR System (BioRad). Ten microliter-reactions were performed using Hard-Shell® Thin-Wall Skirted 384-Well PCR Plates (BioRad) with each reaction containing 2µl cDNA at the different dilutions as previously indicated (2µl 5×qPCR Mix, 4 µl of forward and reverse primers; final concentration of 700 nM) and 2µl of DNAse free water.

Amplification was performed as follows: 12 min at 95°C followed by 40 cycles at 95°C for 25 sec, 60° C for 20 sec and 72° C for 45 sec; dissociation curve as 65°C for 31 sec; 65°C for 0.05 sec + 0.5°C/cycle ramp 0.5°C/sec. Threshold cycle (Ct) data were collected automatically by the software supplied with the Bio-Rad thermal cycler. Background-corrected raw fluorescence data were exported from the Bio-Rad thermal cycler.

Most of the primers evidenced an amplification efficiency comprised between 105% and 95%. Primers used for the amplification of gene *Ov-Gsk3b* and *Ov-Klhdc* showed a higher amplification efficiency (i.e., 113% and 124% respectively), while those for the amplification of *Ov-MTX1* (94%), *Ov-RSU1* (92%), *Ov-flr* (94%), *Ov-ube2c* (92%) genes showed a lower amplification efficiency (Supplementary Table 3). Primers showing an amplification efficiency out of this range (n=6) were not considered in this study: i.e. *Ov-Gsk3b*, *Ov-Klhdc*, *Ov-MTX1*, *Ov-RSU1*, *Ov-flr*, *Ov-ube2c*.

Supplementary Table 4 – List of primers

Supplementary Table 4. Transcript ID, gene name, primer sequences, primer length, GC content, primer Tm, amplification efficiency, product Tm, amplicon size and sequence of each gene used in the standard PCR and real-time qPCR reactions. The sequences were derived accessing *O. vulgaris* transcriptome (Petrosino, 2015; Petrosino et al., 2022). In the column of the primer sequences, for previously published studies, an X (in blue) correspond to a base missing, while a boldface red base means a substitution from published sequence.
In the table highlighted rows (in light grey) marks primers excluded from experiments because efficiency (in red, boldface) ranged out of 98-102 interval.
*F an R have been inverted from previously published data to match 5' 3'.
**Gene names are based on *Mus musculus.*

transcript ID	Gene name**			Primer sequence (5'–3')	Primer length (bp)	GC %	Tm (C°)	Amplification efficiency	R2	Amplicon Tm	Product length (bp)	Product seq
c31322_g1_i1	*Ov-prrc1*	Protein PRRC1-A	F:	CTTCCATCCGAGACAAAAGC	20	50.0	59.8	99%	0.95	81.5	157	CTTCCATCCGAGACAAAAGCTTCCCATCAGATCATGTAGAAACACCAATAATACCAAGAGCTGAAACATCTACTGTCCCACAACAAGATATGACACAAGGTCACAGCAGTTTATTTGGTTGGTTTTCTGGTAGCAATTTTGTTTCCCGTGTTGTTGA
			R:	TCAACAACACGGGAAACAAA	20	40.0	60.0					
c31554_g1_i3	*Ov-gk5*	Putative glycerol kinase 5	F:	TGTGTACTGGAATGGGCAAA	20	45.0	60.0	98%	0.84	81.5	146	TGTGTACTGGAATGGGCAAAAAGTTTAAGTATATTTGAAGAAGAATCCAAGCTAGCAGACATTGCAGAATCAGTTCCTGATAACGGTGGGGTTTATTTTGTGGCTGCATTCAGTGGATTACAGGCTCCAATCAACGATGACACAGC
			R:	GCTGTGTCATCGTTGATTGG	20	50.0	60.1					

transcript ID	Gene name**			Primer sequence (5'–3')	Primer length (bp)	GC %	Tm (C°)	Amplification efficiency	R2	Amplicon Tm	Product length (bp)	Product seq
c17784_g1_i1	*Ov-Klhdc*	Kelch domain-containing protein 4	F:	CTGGAATGGGAGGAATCAGA	20	50.0	60.0	124%	0.9	81	188	CTGGAATGGGAGGAATCAGATTCTTCAGATGATGATAAAGAAAGTGACAGTTCGGATACTGAATCTGACATGGATGTGGACGACAATGTTCCAGATATAAATGATGAAGAAAGTGAAGAAAAATACTTTGAACGTACACAAGAATTCTGGCTTCAGCAAGCCAAAGAGAATGCTGAGGCTGAAAAGGA
			R:	TCCTTTTCAGCCTCAGCATT	20	45.0	60.0					
c28856_g1_i2	*Ov-CUL1*	Cullin-1	F:	GGGTTCGTCGAGAATGTGAT	20	50.0	59.9	101%	0.99	80.5	150	GGGTTCGTCGAGAATGTGATGAAGGAACTAAAGGAATTTATGAAATTTATTCTTTGGCCTTAATTACTTGGAGAGACAACCTGTTTAAGCCATTGAACAAGCAGGTCACTAATGCTGTTCTTGCACTTATTAAAAGGGAGAGGCATGGTG
			R:	CACCATGCCTCTCCCTTTTA	20	50.0	60.1					
c30400_g11_i1	*Ov-wdr44*	WD repeat-containing protein 44	F:	GCTGAAAGTGGTGCTGTGAA	20	50.0	60.0	102%	0.99	81	181	GCTGAAAGTGGTGCTGTGAACTCAGATCAAGATTCGGTATCTCCTATCATGGAACAAAATACATTATCTTCTTTAGAAAGTGATGGTGTTTTACTAAGGCAAAAGTCTTTGCAAGATTTTCCAAAAAGTAACAGCCTGCCCAGTGATTACAGTCAAATGCTAAGACCACGTTCAAATTCCG
			R:	CGGAATTTGAACGTGGTCTT	20	45.0	60.0					
c30725_g11_i1	*Ov-timm*	Mitochondrial import inner membrane translocase subunit Tim22	F:	CTTGGTGGTGCTTTTGGTCT	20	50.0	60.1	99%	0.99	81	107	CTTGGTGGTGCTTTTGGTCTATTTACTGCTGGTATTGACCCAATGTCAACTATGAGCACAGAGACTCCAACTACTAAATTGGTACTAAAAGAAATGAAGGCACGCAC
			R:	GTGCGTGCCTTCATTTCTTT	20	45.0	60.3					
c31227_g1_i2	*Ov-tollip*	Toll-interacting protein	F:	AATGTTTTACGCTCCCATGC	20	45.0	60.0	100%	0.98	84.5	176	AATGTTTTACGCTCCCATGCAGCCTGTAGTTGGTGGCACTCCAGTGTATCAAACTACCCAGCTGCCTCCAGCCCCTGTACAACAAGCTCCAGCATACACAGAATCTGATGTTCAACAAATGAAGGATATGTTCCCAGGAATTGAAACCAATGTCATTCAGTCAGTTTTCATTGCGC
			R:	GCGCAATGAAAACTGACTGA	20	45.0	60.0					
c32096_g14_i2	*Ov-Canx*	Calnexin	F:	TAGGCAGCTCAGCAAAGGAT	20	50.0	60.1	97%	0.99	84	105	TAGGCAGCTCAGCAAAGGATGAAACTGCGGAGAGGAAGAAAACCGATGCGCCATCACCTGATGACAAAGTTAGCGATGATTCTGGCAAGGCGAAAGGTGATTCTG
			R:	CAGAATCACCTTTCGCCTTG	20	50.0	60.8					

transcript ID	Gene name**			Primer sequence (5'–3')	Primer length (bp)	GC %	Tm (C°)	Amplification efficiency	R2	Amplicon Tm	Product length (bp)	Product seq
c33604_g6_i1	*Ov-CHCHD7*	Coiled-coil-helix-coiled-coil-helix domain-containing protein 7	F:	ATTGTCAGGGAACGAAAACG	20	45.0	60.0	99%	0.95	83.5	181	ATTGTCAGGGAACGAAAACGAGCTGGGATAACACCTAATCTTCCTTCAATCGAAGATCGAGAAGAAATCAGAAGGACTAGATTTGGACCTGTACTGTAAGAGCTGATTCCATTCCTGAGATTGGACTGTCATTTTGGACTTGTAAAACCTCAGAATATTCATAGTGATGCAATCCAGCCTG
			R:	CAGGCTGGATTGCATCACTA	20	50.0	59.8					
c33913_g6_i1	*Ov-PTPN12*	Tyrosine-protein phosphatase non-receptor type 12	F:	GGGAATTGCGTCTACAAGGA	20	50.0	60.1	102%	0.99	82	164	GGGAATTGCGTCTACAAGGAATCAAAGACAAGGAGGAGGATACATATCCGGCTGAAGAAGGCAAACAGATCTTTAACATCAAAAAGAACAGATATAAAGATATCATACCATATGACGCCACCCGGGTGAAGTTAAAATCCGTCCCAAATGTACCTGGCTCGGAT
			R:	ATCCGAGCCAGGTACATTTG	20	50.0	60.0					
c35499_g5_i1	*Ov-ube2c*	Ubiquitin-conjugating enzyme E2 C	F:	GTATCGGCATTTCCTGATGG	20	50.0	60.3	92%	0.99	82.5	165	GTATCGGCATTTCCTGATGGAGATAACGTCTTCAAATGGATTGGAACAATAGAAGGTGCTCCTGGAACGGTCTATGATGGATTACGGTTTAAATTAGTGTTGCAATTTCCAAGCGGTTACCCATATCAGGCACCATCAGTTAAGTTTGATACCGGGTGTTTCCAT
			R:	ATGGAAACACCCGGTATCAA	20	45.0	60.1					
c35786_g9_i1	*Ov-Naa15*	N-alpha-acetyltransferase 15 NatA auxiliary subunit	F:	TGTGCTGCTTTGCATAAAGG	20	45.0	60.0	98%	0.98	81	132	TGTGCTGCTTTGCATAAAGGGGTTCCACCATTGTTTATTCGTTTGAAAGAACTTTATAAATCTCCAGAAAAAGTTAAGATAATCGAAGATCTCATACATGGTTATCTGGATTGCTTGAAGTCCCATGAACGT
			R:	ACGTTCATGGGACTTCAAGC	20	50.0	60.1					
c36083_g5_i1	*Ov-SUCLG2*	Succinate--CoA ligase [GDP-forming] subunit beta, mitochondrial	F:	TCACCCGAAGCCATTTTTAC	20	45.0	59.9	101%	0.98	84	200	TCACCCGAAGCCATTTTTACGGAACCAATCGATATCTTTACTGGGATTTCTAAGGAACAGGCTCTCAAGATGGCTTCCAACCTTGGCTTCAAAAGTAATTTGCATGAAGAAGCTGCCAACCAAATCTGCTGTTTGTACAACCTGTTTCAAAATGTCGATGCAACCCAAGTAGAGGTGAACCCTTTTGGGGAAACTCATGA
			R:	TCATGAGTTTCCCCAAAAGG	20	45.0	59.9					
c35707_g2_i1	*Ov-PRMT5*	Protein arginine N-methyltransferase 5	F:	TGACCCGCATTCTCAATACA	20	45.0	60.1	104%	0.99	82.5	172	TGACCCGCATTCTCAATACACATATGATCCATTCTTACTGGCAGCAACAATACTGGATCCAAGTACCTCTCATCGCTCCTGAATTCCAAACAGAAAACCTTATTGAAGGAATAGATGAAGAAATTGACAGAGATCACCGTGACTACAATACCTGGTACTGGTGGCACACATT
			R:	AATGTGTGCCACCAGTACCA	20	50.0	59.9					

transcript ID	Gene name**			Primer sequence (5'–3')	Primer length (bp)	GC %	Tm (C°)	Amplification efficiency	R2	Amplicon Tm	Product length (bp)	Product seq
c36175_g1_i1	*Ov-BTBD17*	BTB/POZ domain-containing protein 17	F:	CTGGTGGTTGGCAAGAAAGT	20	50.0	60.1	100%	0.99	81.5	104	CTGGTGGTTGGCAAGAAAGTCTTCCTTGTACACAAACTCATTCTCAGCATGTGTAGTGATGTTTTCAAGACTATGCTGACCAATCCTCAATGGCCAGAATCCTA
			R:	TAGGATTCTGGCCATTGAGG	20	50.0	60.0					
c25466_g1_i1	*Ov-Ltv1*	Protein LTV1 homolog	F:	CAGTGGTGCCAAGAAGTGAA	20	50.0	59.9	100%	0.97	80	114	CAGTGGTGCCAAGAAGTGAAGCACTGAAGGACATTGACGATCAATTTGAAAAGTTCTTTGAGCAGTATGACGATAATGAAATTGGAGCATTAGATATGGACGACATTGAAGGCA
			R:	TGCCTTCAATGTCGTCCATA	20	45.0	60.1					
c29044_g1_i1	*Ov-rpf1*	Ribosome production factor 1	F:	CGCGAGACAATACCCAAAAT	20	45.0	60.0	101%	0.99	82	160	CGCGAGACAATACCCAAAATATTGATCACAACTTCATGTCGATCCAAACTACGGACTTACCATTTTTGTAAAGAACTTACAACTGTCTTTCCAGATGCAACTTATTATCACCGGAGAACCTTAGCCATAAAGAAGATCGTACCTCAAGCGATCGAGAAAG
			R:	CTTTCTCGATCGCTTGAGGT	20	50.0	59.6					
c29430_g1_i1	*Ov-CG9286*	Protein BCCIP homolog	F:	TGATGCCGATGCTAATGGTA	20	45.0	60.1	98%	0.99	83.5	194	TGATGCCGATGCTAATGGTATCCGTACTCTACTCTCACAGCTGTTCCTGAAAGCAAATATCAACCTTGGTCAGTTGGCCGACACCATCATATCACAAAATTACGTTGGATGTGTCCTCAAGCAATGTGAAGTTGAAGAAGATGAAAGTGATGACGGCATTGATGAGGATCCAATTTTTGGTGTGACGACGGTTA
			R:	TAACCGTCGTCACACCAAAA	20	45.0	60.0					
c29524_g1_i1	*Ov-EIF3M*	Eukaryotic translation initiation factor 3 subunit M	F:	GGACTGGACGAAGCATCCTA	20	55.0	60.2	99%	0.99	82	109	GGACTGGACGAAGCATCCTACCTGCGTTACACTGTCTACTACAGTATGTTACGTCTAGGGAAACAGGCTGAGCTTTTACAAATGGTACCCACTGACCTGAGCAAGGTGA
			R:	TCACCTTGCTCAGGTCAGTG	20	55.0	60.0					
c30066_g9_i1	*Ov-NOB1*	RNA-binding protein NOB1	F:	AACAGCGGGCAACTAAGAAA	20	45.0	59.9	101%	0.98	81	176	AACAGCGGGCAACTAAGAAAGCTTTGCAGAGACTAAATGTCTTTGATGAAAATTATGTTGTTGGACAATCTCCATTTAGAATCCACGACTTGACTAGTAGAGCTGCACAGCTTGGAATTAAAGGACAGGAGGTTAAACCATGGAACAGAAGAAACCCCAATGAAGGGGTCAGAAAA
			R:	TTTTCTGACCCCTTCATTGG	20	45.0	59.9					

transcript ID	Gene name**			Primer sequence (5'–3')	Primer length (bp)	GC %	Tm (C°)	Amplification efficiency	R2	Amplicon Tm	Product length (bp)	Product seq
c31610_g1_i1	*Ov-slc25a40*	Solute carrier family 25 member 40	F:	GGAAACCTATGGTTGCTGGA	20	50.0	59.9	98%	0.97	82	154	GGAAACCTATGGTTGCTGGAATGGTTG<br>CCAGAGTTTGGGCTGCAACTGTGATAA<br>GTCCCTTGGAACTAGTACGAACCCAAC<br>TCCAGTCTTCGAATTTCAGTTATTCCAT<br>GATTGGAGGGTTTATCAAAAATGAAGT<br>TTCTCAAAGCGGTCTTGC
			R:	GCAAGACCGCTTTGAGAAAC	20	50.0	60.0					
c32170_g13_i2	*Ov-Dap3*	28S ribosomal protein S29 mitochondrial	F:	TCGTGTTCTGGTAGCTGTCG	20	55.0	60.0	100%	0.99	81	200	TCGTGTTCTGGTAGCTGTCGATGGTGT<br>GAATGGACTCTGGGGCACAACTAGCGT<br>CAAAGACGAGAATTATGACTATATATC<br>CTGTGATAAAATATCTCTTTTCCACAAT<br>TTTAGGAAAATATTGAAAAATGATTGG<br>TCCAATGGTATTGTTGCTGGAATTGTG<br>GATATTGTAGCCAATCCAAGTGACCAA<br>CGCGAGAAGT
			R:	ACTTCTCGCGTTGGTCACTT	20	50.0	59.9					
c32751_g1_i1	*Ov-flr*	Actin-interacting protein 1	F:	GGTTCTCCAGCTCACAAAGG	20	55.0	59.8	94%	0.99	82.5	163	GGTTCTCCAGCTCACAAAGGTGGTATT<br>TATGGGATTGCTTTCAGTCCAGACAATT<br>CTGAGGTGCTGACAGTCTCTGGTGACA<br>AAACTGCCAAGATCTGGGATGCCAATA<br>CGTATACTTTGGTCAAGGAATTTGTGTT<br>TGGGAAGACACTGGACGACATGTTGG
			R:	CCAACATGTCGTCCAGTGTC	20	55.0	60.0					
c33222_g7_i1	*Ov-RIOK2*	Serine/threonine-protein kinase RIO2	F:	CCCTTCACACTCGTGGATTT	20	50.0	60.0	100%	0.99	80	115	CCCTTCACACTCGTGGATTTCCAGTTCC<br>TAAACCCATTGATTTTAACAGGCATGCA<br>GTTATAATGGAACTCATTGATGCATATC<br>CAATGTGCCAAGTTCACGATGTTGGTG<br>ATGC
			R:	GCATCACCAACATCGTGAAC	20	50.0	60.0					
c34059_g14_i1	*Ov-ATPAF2*	ATP synthase mitochondrial F1 complex assembly factor 2	F:	ATTTGCTCTGCCTTGCATCT	20	45.0	60.0	100%	0.99	82.5	190	ATTTGCTCTGCCTTGCATCTCAGATGCC<br>ACGATAGGTGGAATACATACACAACTG<br>AATTCATACACCATGTGGGGGTTAACA<br>GGGATTTTCAGTGCTGTTGAATGTGTG<br>AAGTCTGTGATACTAACTATGGGTTTA<br>GTCGAGCGCCACCTGACTGTGAATGAT<br>GCTGTATTTCTTTCCCGTTTGGAGATG
			R:	CATCTCCAAACGGGAAAGAA	20	45.0	60.0					
c34087_g16_i1	*Ov-CSDE1*	Cold shock domain-containing protein E1	F:	AGGGACCCTACAGGGAAAGA	20	55.0	59.9	100%	0.99	82.5	167	AGGGACCCTACAGGGAAAGATTATTCG<br>ACCCTTACGCATTGTCAATCCTGAACAG<br>GATGAATATAGTGGTCTTGTACAAGTT<br>GGTTTGGATGAAGATGAAGAGGAAGT<br>GTACCAATATGGTATCACTAGCCTGGCT<br>GATAAACGAGATTTCCTCCAGAAGGGT<br>GATG
			R:	CATCACCCTTCTGGAGGAAA	20	50.0	60.0					

transcript ID	Gene name**			Primer sequence (5'–3')	Primer length (bp)	GC %	Tm (C°)	Amplification efficiency	R2	Amplicon Tm	Product length (bp)	Product seq
c34776_g5_i1	*Ov-usp10*	Ubiquitin carboxyl-terminal hydrolase 10	F:	CTCCACCACCTCCATCAGTT	20	55.0	60.0	100%	0.98	85.5	169	CTCCACCACCTCCATCAGTTGTTCCTGTTGCAACTCTGTACCCACGCACAGGACCTGTTCCTCACATCCCTGCTGGAGGTGCTATTTTCCAGTCTCCACTAGTACCTTTGGTATTTACACAACCAGTCAACATTTCTCAGCCCCAAATAAACAGACCCTCAGTGGTTGG
			R:	CCAACCACTGAGGGTCTGTT	20	55.0	60.0					
c35010_g2_i4	*Ov-EIF2A*	Eukaryotic translation initiation factor 2A	F:	GAACCGGTTTGCTTGTGATT	20	45.0	60.0	101%	0.99	82.5	173	GAACCGGTTTGCTTGTGATTACATCAACAGAAACTTCAGACAAGTCATATTATGGAGAAACTGGCCTTCATTACCTGAGTATAAAAGGTGAAGGTTGCCTTGTACCCAGAGCTAAAGACGGTCCTGTCTACTGTGTGGAGTGGCATCCCAATTCTACCCAATTCTGCGTGGTT
			R:	AACCACGCAGAATTGGGTAG	20	50.0	60.0 °C					
c35032_g7_i2	*Ov-Dnaja3*	DnaJ homolog subfamily A member 3, mitochondrial	F:	AACCGTCATTTACCGTCTGC	20	50.0	60.0	100%	0.98	83	182	AACCGTCATTTACCGTCTGCTCGTCTTTCTAACTATCAATTTACATCAAGTCGATGGTTCACTACGCTAAATGTAGCTCTCTACACAGAGGTCTCGTCACGGCAAGAAAAACTGAAGAAATCTTTAATACCAACGACACATGTGCGACTTATTCATTCAAGCCAGACTCGCAACAAACAGGA
			R:	TCCTGTTTGTTGCGAGTCTG	20	50.0	60.0					
c35311_g1_i1	*Ov-CPIJ005834*	Elongation factor G mitochondrial	F:	ATCCCGAGAGCAGAGAAACA	20	50.0	60.0	102%	0.99	83.5	159	ATCCCGAGAGCAGAGAAACATTGATCTCTGGCATGGGTGAACTGCATTTAGACATCTATACTCAGCGCTTGGAACGGGAATACAATGCCAAATGTGTTCTTGGGAAACCTAAAGTTGCTTTCCGAGAAACTCTTTTAGCTCCTTGCGAATTCGATTACC
			R:	GGTAATCGAATTCGCAAGGA	20	45.0	60.0					
c34313_g4_i1	*Ov-Ppm1b*	Protein phosphatase 1B	F:	TCCAGGAGCACCTAAGCTGT	20	55.0	60.0	101%	0.99	83	149	TCCAGGAGCACCTAAGCTGTCAGATGAGGCAATAAGGAAAGAAAGAGAACTGGACACACGTTTGGAGAACAAAATTAAAGAAATCCTAGACAACTGCAAACCTGAAGATGATGTCGACCTTTCTCTTGTGATGCACGAGTTGATGGATG
			R:	CATCCATCAACTCGTGCATC	20	50.0	60.1					
c29941_g6_i1	*Ov-14-3-3zeta*	14-3-3 protein zeta	F:	GTTGCCCAAGGTGAAGAGAG	20	55.0	59.8	97%	0.99	81.5	177	GTTGCCCAAGGTGAAGAGAGGAAAGGTGTTTTAGGGCACTCACAATGTGCATACAAAGACGCTTTTGAAATTGCTAAAAACCAAATGCAACCTACACATCCAATTCGACTGGGATTGGCATTGAACTTTTCTGTATTTTATTATGAAATTCTTAATGCTCCAGAAAAAGCCTGTTGC
			R:	GCAACAGGCTTTTTCTGGAG	20	50.0	60.0					

transcript ID	Gene name**			Primer sequence (5'–3')	Primer length (bp)	GC %	Tm (C°)	Amplification efficiency	R2	Amplicon Tm	Product length (bp)	Product seq
c30691_g3_i1	*Ov-RSU1*	Ras suppressor protein 1	F:	GAAATAGGCAGCCTTCAACG	20	50.0	59.8	92%	0.99	82	166	GAAATAGGCAGCCTTCAACGCGTAAGGGAATTATATATTCAAGGAAATCGGCTGAGCACTTTACCACCTGAACTAGGTCAACTTGATTTATATGGAACAAAACAAATTTTTCGGTGTGAAAATAACCCATGGGTAGCCCTGATTGGTGACCAGCTTCAGATTGGTG
			R:	CACCAATCTGAAGCTGGTCA	20	50.0	59.8					
c31105_g4_i1	*Ov-BTBD2*	BTB/POZ domain-containing protein 2	F:	AGTTCGAAACCGACATCCAG	20	50.0	60.1	99%	0.99	84	173	AGTTCGAAACCGACATCCAGCTTTTCAGATGTGCCCCGTTGCAGTTTGACTGGCAAAGAACAAGTGATATCACGTTTCTGTCAAATTGAGAGCCGATGGGGTTACAGCGGCACAAGCGATAGAATCAGGTTTATGGTGAACCGAAGAATTTTTGTTGTTGGGTTTGGCCTCTA
			R:	TAGAGGCCAAACCCAACAAC	20	50.0	60.0					
c32222_g5_i1	*Ov-UBE2F*	NEDD8-conjugating enzyme UBE2F	F:	AGAAAACCTGCCCAAAACCT	20	45.0	60.0	98%	0.99	80.5	106	AGAAAACCTGCCCAAAACCTGCAATATTACATTTGAAGATTCTAATGTCCTCCACATGTTTGATCTGTCCATACATCCAGATGAAGGTTATTGGCATGGCGGTACT
			R:	AGTACCGCCATGCCAATAAC	20	50.0	59.9					
c32350_g3_i1	*Ov-nAChRalpha1*	acetylcholine receptor subunit alpha-like 1	F:	CAGCAACGAAAGCCACAGTA	20	50.0	60.0	97%	0.97	82.5	198	CAGCAACGAAAGCCACAGTATATTCCAACGGCAAAATCTCATGGGAACCTCCAGCAATTTATAAAACATACTGTCCAATTGATGTTGAATACTTCCCATTTGATATTCAAAAATGTATGATGCGGTTCGGTTCGTGGACCTACGATGGCCTGGAAGTTGATTTAGTCCACTTCTGTCACGAACAAGGGACGAACAAAT
			R:	ATTTGTTCGTCCCTTGTTCG	20	45.0	60.0					
c32876_g12_i1	*Ov-RNF7*	RING-box protein 2	F:	TGTGGAGTTGGGATGTTGAA	20	45.0	59.9	101%	0.99	82	159	TGTGGAGTTGGGATGTTGAATGCGATACATGTGCTATTTGTCGTGTTCAAGTAATGGATGCATGTTTACGCTGTCAGTCAGAAAATAAACAAGATGACTGTGTTGTGGTATGGGGTGAGTGCAATCACTCTTTTCATAACTGCTGCATGTCATTATGGG
			R:	CCCATAATGACATGCAGCAG	20	50.0	60.1					
c32876_g7_i5	*Ov-KCMF1*	E3 ubiquitin-protein ligase KCMF1	F:	TGCAGGTGGTGGTACTCAAA	20	50.0	60.2	100%	0.99	82.5	157	TGCAGGTGGTGGTACTCAAAATATCACAGTGATCAAACAGATGTCAGCTTCTTCTAATTCTGCTTCCCGTAGTGCATCAGGGACAAGTCCGATCTCTGCAACTTCTCAGAGTTCTTCACTTACACAAGCCAATTTAGTATTGCTTTCTGGTCAGGGG
			R:	CCCCTGACCAGAAAGCAATA	20	50.0	60.1					

transcript ID	Gene name**			Primer sequence (5'–3')	Primer length (bp)	GC %	Tm (C°)	Amplification efficiency	R2	Amplicon Tm	Product length (bp)	Product seq
c33117_g3_i1	*Ov-Rnd3*	Rho-related GTP-binding protein RhoE	F:	CAGTCGCAGATCCAGATTCA	20	50.0	59.9	101%	0.99	83	150	CAGTCGCAGATCCAGATTCACTAGAACATGTCATGTCAAAGTGGTTTCCTGAAGTTCGAGAACACTGTCCAAGTCAACCCATTATGCTCGTTGGATGTAAAACAGACTTACGATCAGACCTGCAGTGTATATCAGCCTTGGCAAAGAAAA
			R:	TTTTCTTTGCCAAGGCTGAT	20	40.0	59.8					
c33305_g9_i1	*Ov-Abi2*	Abl interactor 2	F:	AATCCACGGTCAGGTCGTAG	20	55.0	60.0	100%	0.99	85.5	100	AATCCACGGTCAGGTCGTAGTGCTTCTATGAGCAGTGGGACTTCCACTCAAGCCCCTACATCCAAACCACCAACCCCACCAACAGTCCGTGCATGTGGTA
			R:	TACCACATGCACGGACTGTT	20	50.0	60.0					
c34295_g8_i1	*Ov-Vbp1*	Prefoldin subunit 3	F:	CCAAAGAGCCATTTGAAACC	20	45.0	59.5	99%	0.99	84	136	CCAAAGAGCCATTTGAAACCCAATTTTTACTGTCTGACTTGTTATATGCAAAGGCTAAAATCCCACCAACAGATAAAGTCTGCCTTTGGCTTGGGGCAAATGTGATGCTGGAGTATAGTCTGGACGATGCACAAGC
			R:	GCTTGTGCATCGTCCAGACT	20	55.0	61.4					
c34563_g2_i1	*Ov-PCK1*	Phosphoenolpyruvate carboxykinase cytosolic GTP	F:	GAAGTTCATGTGGCCAGGTT	20	50.0	60.0	102%	0.98	83	108	GAAGTTCATGTGGCCAGGTTTTGGTGAAAATTGCCGTGTCTTGGACTGGATATTGCGTAGAATAAACCAAGAGGAGTGTGCTGTTTCTTCAGCAATTGGGTACCTTCC
			R:	GGAAGGTACCCAATTGCTGA	20	50.0	59.9					
c35194_g4_i1	*Ov-C2CD2*	C2 domain containing protein 2 X1	F:	CAGGTGACCCATTCTGTGTG	20	55.0	60.0	100%	0.99	83.5	173	CAGGTGACCCATTCTGTGTGATTATAATGGATAGCCCCTACCAAAAATATGAGACCACAGTCGTCAGAAATACTGTCAGTCCATTTTGGGACGAACACTTCCTCTTGGATGTTTCTCCAGCATCAACCCTTCTTCGTTTCGAGTTGTATGACAAAGGAAAGTCCCCAGCTGAT
			R:	ATCAGCTGGGGACTTTCCTT	20	50.0	60.1					
c35789_g7_i1	*Ov-MRM2*	rRNA methyltransferase 2 mitochondrial	F:	AATGCTCAACGGACGTAAGG	20	55.0	60.1	98%	0.98	83	135	AATGCTCAACGGACGTAAGGCAGACATCATTCTCAGCGACATGGCCCCGAATACTACAGGAATCCGTTCAATGGACCATGATCTTATTATAGAACTTTGTTTTTCTGTTCTCGAATTCTCTCACAGCGTTCTCCA
			R:	TGGAGAACGCTGTGAGAGAA	20	50.0	59.7					
c36050_g13_i1	*Ov-Sdhd*	Succinate dehydrogenase ubiquinone cytochrome b small subunit mitochondrial	F:	GGTGGGACTACTTTCGACCA	20	55.0	60.0	101%	0.99	83	100	GGTGGGACTACTTTCGACCACGTTTAGGGCCATTTACTCCCGCTGAGAAGAAAGGAAAACATATGATGCTGGCCTCTACTCATTGGAAACTCGAACGGAT
			R:	ATCCGTTCGAGTTTCCAATG	20	45.0	59.9					

transcript ID	Gene name**			Primer sequence (5'–3')	Primer length (bp)	GC %	Tm (C°)	Amplification efficiency	R2	Amplicon Tm	Product length (bp)	Product seq
c26803_g1_i1	*Ov-RAD23B*	UV excision repair protein RAD23 homolog B	F:	TCAACATCCCAGGGAGAGAC	20	55.0	60.0	102%	0.98	85	133	TCAACATCCCAGGGAGAGACTGCTAGTGCTGCCTCATCAGAGAAGTCTGTACCTGCTACCACTCAGGGAGCATCAGCCACCCCTACATCAGACAGTGGCTCTTCCCAAAGTTCGAAAGGCTCGCAAGGTAGTG
			R:	CACTACCTTGCGAGCCTTTC	20	55.0	60.0					
c28702_g2_i1	*Ov-Abhd18*	Protein ABHD18	F:	TGCGAGATGTGATGGAAGAG	20	50.0	59.9	100%	0.89	84	163	TGCGAGATGTGATGGAAGAGTTCACTCACCTTGGCAATTACACACAACCCGTAGATACTAGTTTGATTATCATTGTGGCTGCAAAACAAGACGCTTACATTCCTCATGAAGGGGTCCTCAGTCTGAAGCAGCTATGGCCTGAAGCTGAAGTGCGCTACATTGA
			R:	TCAATGTAGCGCACTTCAGC	20	50.0	60.2					
c28934_g1_i1	*Ov-UGP2*	UTP--glucose-1-phosphate uridylyltransferase	F:	AAAGCGGTTTCTTCCAGTGA	20	45.0	59.9	102%	0.99	79.5	100	AAAGCGGTTTCTTCCAGTGAAAACTACATCTGATTTACTTCTAGTGATGAGCAATTTATACAAAATGAGAACCGGGGCTTTAGAGATGAGTCCCCAACGC
			R:	GCGTTGGGGACTCATCTCTA	20	55.0	60.2					
c34071_g2_i1	*Ov-mts*	Serine/threonine-protein phosphatase PP2A	F:	GGTCGATCACCAGACACAAA	20	50.0	59.5	102%	0.98	79.5	198	GGTCGATCACCAGACACAAATTACCTTTTCATGGGAGATTATGTTGACAGAGGATACTATTCAGTTGAAACTGTCACATTGTTGGTAGCTCTAAAAGTAAGATATAAGGACAGAATAACTATACTTCGAGGAAATCATGAAAGTAGACAGATTACCCAAGTGTATGGATTTTATGATGAATGTTTGAGGAAGTATGGG
			R:	CCCATACTTCCTCAAACATTCA	22	40.9	59.9					
c34932_g8_i1	*Ov-MTX1*	Metaxin-1	F:	CTCCACTGAGCAGCAATCAA	20	50.0	60.1	94%	0.94	81	167	CTCCACTGAGCAGCAATCAATTGCAGAACCATTTGCTTGCGTGTACAAAACTGACAGATTTTTGCAAGCGAATCTTACATGATTATTTTCCTCAGAGCCTTCAAGAAAATGAAAGTACGAGTAGAAATACAAGTAGTTTAAGTGAAGACAACCCCAACAAGTTACGG
			R:	CCGTAACTTGTTGGGGTTGT	20	50.0	59.8					
c35016_g13_i1	*Ov-Gsk3b*	Glycogen synthase kinase-3 beta	F:	CCAGACCGACCACAAGAAAT	20	50.0	60.0	113%	0.99	81.5	107	CCAGACCGACCACAAGAAATTGGCTATGCTGATACAAAAGTTATTGGCAATGGCTCATTTGGAGTAGTTTACCAGGCCAAACTTACTGATACGGGTGAACTGGTTGC
			R:	GCAACCAGTTCACCCGTATC	20	55.0	60.4					

transcript ID	Gene name**			Primer sequence (5'–3')	Primer length (bp)	GC %	Tm (C°)	Amplification efficiency	R2	Amplicon Tm	Product length (bp)	Product seq
c35771_g14_i2	*Ov-Gnaq*	Guanine nucleotide-binding protein G(q) subunit alpha	F:	TGATGGTCCAAAGAAAGATGC	21	42.9	60.1	105%	0.97	79.5	146	TGATGGTCCAAAGAAAGATGCACAAGCTGCCAGAGAATTCATATTGCGAATGTTTGTTGATTTAAACCCAGATCCTGATAAAATTATATATAGTCATTTCACATGTGCAACAGATACTGAAAACATCAGATTCGTCTTTGCAGCTG
			R:	CAGCTGCAAAGACGAATCTG	20	50.0	59.7					
c31295_g14_i1	*Ov-AP1M1*	AP-1 complex subunit mu-1	F:	ATTCCAGTCCCACAAGATGC	20	50.0	59.9	104%	0.99	80.5	152	ATTCCAGTCCCACAAGATGCTGACTCCCCTAAATTCAAGACTACTGTTGGAAGCTGCAAATATACCCCAGAATTAAATGCTGTTATCTGGACAATAAAATCTTTTCCCGGTGGGAAAGAATATTTGATGAGAGCTCACTTTGGTCTCCCAAG
			R:	CTTGGGAGACCAAAGTGAGC	20	55.0	59.8					
c28771_g3_i1	*Ov-AP5Z1*	AP-5 complex subunit zeta-1	F:	TGAACTGCTGGCTTCAGATG	20	50.0	60.1	101%	0.94	83	165	TGAACTGCTGGCTTCAGATGAGGATGAAGATGGTAACCATATGGCTGAGATGTTCAACAACAGTGTTGATGTTTCCAAGGCCATCGACAGTTTACCTTTCTATTTGGTACAGGGACAAAAGATTGGTTGTTTGGCCAGGATGGTTTCTGAAGCTGTAGGGTGGCT
			R:	AGCCACCCTACAGCTTCAGA	20	55.0	60.0					
c29565_g1_i1	*Ov-CERK*	Ceramide kinase	F:	AGAAAAATGGCACACGATCC	20	45.0	59.9	105%	0.99	85	159	AGAAAAATGGCACACGATCCGTGGAGATTTTATTGGTGTAAATGCCGTTACCATGAGTTGCCGGTGTGCCATGAGCCCAGAAGGTGTGTCTCCTGCTTGTCATTTGGGAGATGGATGTACCGACCTAATCCTAGTGAAACGTTGTTCCCGGCTAAACTA
			R:	TAGTTTAGCCGGGAACAACG	20	50.0	60.1					
c30947_g6_i1	*Ov-syvn1*	E3 ubiquitin-protein ligase synoviolin	F:	TCCTGGATTTCCACCTATGC	20	50.0	59.9	100%	0.96	85.5	110	TCCTGGATTTCCACCTATGCCGTTATGGGCTCCTAACATGCCTCCACCACCGCCACCACCTCCAGCTCAGACTGCATCCCAACAACAGACAGTCAACCCTGTGTCAACCC
			R:	GGGTTGACACAGGGTTGACT	20	55.0	59.9					
c32955_g4_i1	*Ov-Homer2*	Homer protein homolog 2	F:	TGGCCAGTGGTCAGATGTTA	20	50.0	60.1	101%	0.95	80	198	TGGCCAGTGGTCAGATGTTAGTGCCAATATTGTGTATGGACTTGGTTTCAGTGGAGAAAAAGAACTCAATCAGTTCCATGATTATTTTGAGGAGGTGAAATTACTTACAAGAAAACATTCAAAATCTAAGCAAGAAATAAATGGTGCTATGGACAATGATACTTTGAAAAAAACAGGTTCCTCACCAATCAACCCATT
			R:	AATGGGTTGATTGGTGAGGA	20	45.0	60.2					

transcript ID	Gene name**			Primer sequence (5'–3')	Primer length (bp)	GC %	Tm (C°)	Amplification efficiency	R2	Amplicon Tm	Product length (bp)	Product seq
c34716_g8_i1	*Ov-USP15*	Ubiquitin carboxyl-terminal hydrolase 15	F:	ACAGCAACCAGTGGGAAATC	20	50.0	60.0	97%	0.99	80.5	100	ACAGCAACCAGTGGGAAATCGAAGACAGAAATAAAGCAAACGTGTGAAGACAACATCAATACAGACATTGAAACCGAAGAAAATGAGAACCAGCCACCAC
			R:	GTGGTGGCTGGTTCTCATTT	20	50.0	60.0					
c34844_g11_i1	*Ov-WBP2*	WW domain-binding protein 2	F:	CTCCCCAGGGACCATTTTAT	20	50.0	60.0	99%	0.99	85.5	173	CTCCCCAGGGACCATTTTATCAGCCTGCTCCGCCTGCCTACACAGCTTATAACTATGGCTGGGTGCCATATCAAACATTCCCAAATGCACCACCAGCACATGAGGTGTTTATGGCAGAAGCACCCCCTCCCTACCCAGGGGTTGATTCGAATTTGAATCCGTATCCGACTGGT
			R:	ACCAGTCGGATACGGATTCA	20	50.0	60.3					
c35037_g6_i2	*Ov-PIP4K2B*	Phosphatidylinositol-5-phosphate 4-kinase type 2 beta	F:	AGCAGCCTGTTGAGATGGAT	20	50.0	59.8	100%	0.91	81	167	AGCAGCCTGTTGAGATGGATTCACCTGGTCGTAGTGGTGCCCGCATGCTATTGTCACAAGATAAGAAATATTTTATCAAGACATTAGTCAGCGAAGAAGTTGAACAGATGCATCATATTTTGAAACAGTATCACTCATATATTGTTGAAAGACACGCACAGACGTTG
			R:	CAACGTCTGTGCGTGTCTTT	20	50.0	59.9					
c35361_g5_i1	*Ov-Fam160a2*	FTS and hook-interacting protein-like	F:	AAGGAAAAGTGCCAGCTGAA	20	45.0	60.0	101%	0.9	83.5	177	AAGGAAAAGTGCCAGCTGAATGTCTGATATTCTCTTTAATCCTATACATCCATCGAGATGGAAGGGTTGGTCAGCAGGCACGTGAAGCTCTCCTTTTAATTATGGCATTGTCCAACAAATACCCACACATTGGCAGATACATAAGCGACTACTGTCCAGTTCTGGCAACAGGGCTAA
			R:	TTAGCCCTGTTGCCAGAACT	20	50.0	59.9					
c35373_g3_i2	*Ov-Snx20*	Sorting nexin-20	F:	GCCCCTGTCAAACAGTGAAT	20	50.0	60.0	101%	0.99	83.5	181	GCCCCTGTCAAACAGTGAATGAACCATTATTCATGTCCGACCAAGAGGAACTAGCGTCCCTTGCAGACGAAATGGATGATGATAACTTCGCGAACCTGGGGACTTTGTCATTTTCAGAAGATGAAGAAGCTCGAAATTCTGCCACTGATATCGTCAAGACAGAACCGGCATCTTATCCAGA
			R:	TCTGGATAAGATGCCGGTTC	20	50.0	60.0					
c35896_g5_i1	*Ov-Snx25*	Sorting nexin-25	F:	GGAAGGCTCAAGTTCACAGC	20	55.0	60.0	99%	0.99	83.5	181	GGAAGGCTCAAGTTCACAGCGCTGATATTGTTTCAGAAGGAGACAAATTAACTCCTTACTTCGTTATTGGTGTTGATGTTGACGATGCCGACTCATTGATGAAAAGTGAAGGCTGGTCGATTTCTCGAACATTGGCTGATTTTGGTGTTTTGCATGAAAAGCTCATCCAGATTGGTGCATG
			R:	CATGCACCAATCTGGATGAG	20	50.0	60.1					

transcript ID	Gene name**			Primer sequence (5'–3')	Primer length (bp)	GC %	Tm (C°)	Amplification efficiency	R2	Amplicon Tm	Product length (bp)	Product seq
c36137_g10_i4	*Ov-AGL*	Glycogen debranching enzyme	F:	GGTCAGCCAGTTGGTTCATT	20	50.0	60.0	101%	0.99	84	189	GGTCAGCCAGTTGGTTCATTCATCCAG<br>CCTCAGATCCAGCCTCTTGTTAACTCTA<br>TGGCCCATGCTATTTTCTTTGACCAGAC<br>CCATGATAACCCAAGCCCTATTGAGAA<br>ACGCAGTGTCTATGATGTTCTCCCAACA<br>GCTGCTTTGATATCAATGGCTTGTTGTG<br>CATCTGGAAGCAACCGTGGTTAT
			R:	ATAACCACGGTTGCTTCCAG	20	50.0	60.0					
c34695_g13_i5	*Ov-CNBP*	Cellular nucleic acid-binding protein	F:	TGTGGCAAGAGTGGTCACAT	20	50.0	60.2	97%	0.93	86	115	TGTGGCAAGAGTGGTCACATCCAGAGG<br>GAGTGCCTGGTGGCCAGCTCAAAACCA<br>TGCTATCGATGTGGTGTAGCTGGCCAT<br>ATAGCTCGGGAGTGTTCTGATGAGCGA<br>CGAGATG
			R:	CATCTCGTCGCTCATCAGAA	20	50.0	60.1					
c35327_g8_i2	*Ov-FBXO38*	F-box only protein 38	F:	GCACCTCACTGGAGTTGTCA	20	55.0	59.9	104%	0.99	83	118	GCACCTCACTGGAGTTGTCATACCTGA<br>ATTACCTCGTATGCAAACTTTGAAGCAC<br>TTGTATCTACGCTTTGTACGGCTCACTC<br>AACACCAACCATTTCGTGACTTTGCTGC<br>TCCTTCC
			R:	GGAAGGAGCAGCAAAGTCAC	20	55.0	60.0					
c30165_g11_i1	*Ov-wls*	Protein wntless	F:	GTGTGCAGCTCTCACCGTAA	20	55.0	60.1	Not tested for qRT-PCR			175	GTGTGCAGCTCTCACCGTAATCTTCTTC<br>ATCATTGGCCAAGTGAGCGAGGGACA<br>GTGGAAGTGGGGGGAAGAGTCCCCTT<br>CACTTGAATATACCAGTGCCTTCTTCAC<br>AGGAGTATATGGAATGTGGAATGTCTA<br>TGTCTTTGGATTACTCTGTATGTACGCT<br>CCGTCTCACAAG
			R:	CTTGTGAGACGGAGCGTACA	20	55.0	60.0					
c34939_g11_i1	*Ov-ESR16*	Ecdysteroid-regulated 16 kDa protein	F:	TACCCAGGGGTGGACAAGTA	20	55.0	60.2	Not tested for qRT-PCR			126	TACCCAGGGGTGGACAAGTAACTATTG<br>AAGTCGACTTTACTCTTAGTGCTGAAGT<br>CAAAACCGCTCAAGAAAGTGTCTTTGG<br>AATCATCAGCGGCATGCACTTGCCCTTC<br>CCAACAACAAGCCCTA
			R:	TAGGGCTTGTTGTTGGGAAG	20	50.0	60.1					
c35194_g4_i2	*Ov-C2CD2*	C2 domain containing protein 2 X2	F:	TTTAAAAGGGTTCCGGCTCT	20	50.0	59.8	Not tested for qRT-PCR			157	GTGCCGTGTGTCGTAGAAAAACTTAGT<br>CCTTATTCTGAAAGTAGTAAAAACTCTC<br>CAAGTACCAACCAGTCTTCTCCGTCGTT<br>TGGAGGACGGACAGATAAACAGGAAT<br>CTTCAGGGGTTCTACCTGGTTTCTGGG<br>AGATTACACCAACAGGCAGCC
			R:	CACACAGAATGGGTCACCTG	20	55.0	60.5					

transcript ID	Gene name**			Primer sequence (5'–3')	Primer length (bp)	GC %	Tm (C°)	Amplification efficiency	R2	Amplicon Tm	Product length (bp)	Product seq
from previously published studies												
c5816_g1_i1	*Ov-Rps27a (modified Sirakov et al. 2009)**	ubiquitin-40S ribosomal protein S27a (Sirakov et al. 2009)	F:	CCTTCATTTGGTCCTTCGTC	20	50.0	63.8	101%	0.99	81.5	113	CCTTCATTTGGTCCTTCGTCTGCGAGGTGGCGCCAAGAAGCGCAAGAAGAAGAATTACACCACTCCCAAAAAGAATAAGCATAAGAAGAAGAAGGTTAAGTTGGCGGTTTTGA
			R:	TCAAAACCGCCAACTTAACC	20	45.0	64.0 °C					
	Ov-Rps27a (ex-novo design)		F1:	GCCAAGAAGCGCAAGAAGAA	20	50.0	67.0	97%	0.99	80.5	152	GCCAAGAAGCGCAAGAAGAAGAATTACACCACTCCCAAAAAGAATAAGCATAAGAAGAAGAAGGTTAAGTTGGCGGTTTTGAAATATTACAAGGTTGATGAAAATGGTAAAATCACTCGTTTGCGTCGTGAGTGTCCCAACGAGGAATGTGG
			R1:	CCACATTCCTCGTTGGGACA	20	55.0	69.0					
c26807_g1_i1	*Ov-eef1a (modified from Xu and Zheng 2018)*	Elongation factor 1-alpha (Xu and Zheng 2018)	F:	CGTCCCTGT*T*GGCAGAGT*X*	18	61.10%	64.6	103%	0.99	85.5	143	CGTCCCTGTTGGCAGAGTCGAGACCGGTGTCTTGAAGCCTGGTACCGTCGTGACATTTGCACCTGCCATGGTATCCACTGAGGTGAAGTCTGTAGAGATGCACCACGAGTCACTTCCAGAAGCTAACCCAGGAGACAACGTTG
			R:	CAAC*G*TTGTCTCCTGG*GT*TAGC	22	50.0	67.0					
	Ov-eef1a (ex-novo design)		F1:	AAAGGCCGAGAGAGAACGTG	20	55.0	66.0	96%	0.99	84.5	179	AAAGGCCGAGAGAGAACGTGGTATTACCATTGATATTGCTCTCTGGAAATTTGAAACCGACAAGTACTGTATTACCATCATCGATGCGCCGGGCCACAGAGATTTCATTAAAAACATGATCACCGGTACCTCGCAGGCTGACTGTGCTGTGCTGGTTGTTGCTGCGGGTAAAGGTGAAT
			R1:	ATTCACCTTTACCCGCAGCA	20	50.0	67.0					
c12855_g1_i1	*Ov-TUBg1 (modified from Xu and Zheng 2018)*	Tubulin gamma-1 chain (Xu and Zheng 2018)	F:	TGATGGT*C*TCAACGCCTGTT	20	50.0	66.1	100%	0.99	80.5	129	TGATGGTCTCAACGCCTGTTCATAGACAAGACAAGCACTGCTATATTTCAATATTGAATATCATTCAAGGTGAAGTAGATCCTACACAGGTCCACAAAAGTCTGCAGAGAATCCGTGAGCGTAAACTGG
			R:	CCAG*T*TTACGCTCACGGATT	20	50.0	64.3					
	Ov-TuBg1 (ex-novo design)		F1:	AGCTACACTGCGTTACCCTG	20	55.0	62.0	98%	0.99	81	171	AGCTACACTGCGTTACCCTGGATACATGAATAATGATCTTGTAGGCTTGATTGCTTCTCTTATTCCTACACCACGACTTCATTTCTTAATGACTGGTTATACACCATTAACAACTGACCCACATGTTGCCAGTGTGAGAAAAACTACTGTCCTCGATGTGATGCGTAGGCT
			R1:	AGCCTACGCATCACATCGAG	20	55.0	66.0					

transcript ID	Gene name**			Primer sequence (5'–3')	Primer length (bp)	GC %	Tm (C°)	Amplification efficiency	R2	Amplicon Tm	Product length (bp)	Product seq
c34110_g1_i1	*Ov-Mrps5 (modified from Xu and Zheng 2018)*	28S ribosomal protein S5 mitochondrial (Xu and Zheng 2018)	F:	G*T*GGTGTAAGTAA*T*GCTGGTA	21	42.9	57.3	102	0.99	84	139	GTGGTGTAAGTAATGCTGGTAGGAAAAAAGGTAGAGGTCGTGGCCGGAAAAGAAAAGTGGATCTACACATCGGTCAAATTCTCGGCGATGGTCAACAGCAGATGGTTTGGCCCGGACTCAATGCCCCTGTCATGAAAGG
			R:	CCTTTCA*T*GACAGG*G*GCATTG	21	52.4	68.9					
	Ov-Mrps5 (ex-novo design)		F1:	CTTTGCAACGTGGATGGACG	20	55.0	69.0	99%	0.96	82	147	CTTTGCAACGTGGATGGACGGGGAGACGGATGCCCGGTATGAGTATAGGACCTCCAGATCCTTTAGACGATTTTACATTTGAAGGTTTCGATACGAGAGTATTAGAGCTTAAAACCGTAGCCAATATGACAGCAACTCTGGGACGAA
			R1:	TTCGTCCCAGAGTTGCTGTC	20	55.0	66.0					
c36025_g3_i2	*Ov-Tuba1a (ex-novo design)*	Tubulin alpha-1A chain (Sirakov et al. 2009)	F:	GCTGTGTGCATGTTGAGCAA	20	50.0	66.7	98%	0.99	84	159	GCTGTGTGCATGTTGAGCAACACCACAGCTGTTGCTGAAGCTTGGGCTCGTCTTGATCACAAATTTGACTTGATGTATGCCAAGCGTGCTTTCGTTCACTGGTATGTGGGAGAAGGTATGGAAGAAGGTGAGTTCTCTGAAGCTCGTGAGGATTTGGCA
			R:	TGCCAAATCCTCACGAGCTT	20	50.0	67.3					
c2281_g1_i1	*Ov-Rpl6 (ex-novo design)*	60S ribosomal protein L6 (Xu and Zheng 2018)	F:	TCGGGTGTACAAGAGGAGGA	20	55.0	65.0	100%	0.99	82.5	153	TCGGGTGTACAAGAGGAGGAGGATCTTCATCAAGAAGGAACAACCTAAGACAAAAGCAGCTAAGAAGGTCAAACCTGCAAAGTATGTCGTGAAGCCTATTGGTGGTGAGAAAAATGGTGAAACGAGGAAAGTCCGTGTTAAGCGATTGCCTCG
			R:	CGAGGCAATCGCTTAACACG	20	55.0	68.0					
c30772_g3_i11	*Ov-Rpl23 (from Imperadore 2017)*	60S ribosomal protein L23 (Imperadore 2017)	F:	TAATGCCAGCGGTGGTGATT	20	50.0	60	102%	0.99	83	177	TAATGCCAGCGGTGGTGATTCGGCAACGGAAACCTCTACGGAGAAAAGATGGTGTTTTTATATACTTTGAAGACAATGCTGGTGTTATTGTCAACAATAAAGGGGAAATGAAAGGATCAGCCATCACAGGACCAGTTGCTAAAGAATGTGCAGATTTATGGCCCCGTATTGCTTCAA
			R:	TTGAAGCAATACGGGGCCAT	20	50.0	60					
c29373_g3_i1	*Ov-Rps18 (from Imperadore 2017)*	40S ribosomal protein S18 (Imperadore 2017)	F:	CCATGTCGCTCGTAATCCCA	20	55.0	60	99%	0.99	82.5	129	CCATGTCGCTCGTAATCCCAGAGAAGTTTCAGCATATTCTGCGTATTATGAATACGAACATCGACGGTCGTAGGAAGTCGATGTTTGCTATTACTGCCATTAAGGGTATTGGACGACGATTTGCCAATG
			R:	CATTGGCAAATCGTCGTCCA	20	50.0	60					

Amplification of the candidate reference genes

Specific primer pairs were designed for the selected putative reference genes (Supplementary Table 4). For reference genes utilized in previous RT-qPCR experiments in cephalopods and identified from literature (N=8; Table 1), ex novo design of primer couples was performed for *Ov-Rps27a*, *Ov-eef1a*, *Ov-TUBG1*, *Ov-MRPS5*, *Ov-Tuba1a* and *Ov-Rpl6*. *Ov-RpL23* and *Ov-RPS18* primers were synthetized based on published study (Imperadore, 2017), while the primers for *Ov-Rps27a*, *Ov-eef1a*, *Ov-TUBG1*, *Ov-MRPS5* were deduced from other published studies, but were slightly modified to match sequences identified in *O. vulgaris* transcriptome, for a total of 12 primer couples (Supplementary Table 4).

Primer specificity and efficiency were tested through standard PCR and RT-qPCR reactions. Total mRNA was extracted from twelve tissues in five subadult *O. vulgaris* specimens (Fig. 1; Supplementary Table 3). Each sample was retrotranscribed to single strand cDNA and pooled to test primers.

The primer sequences, amplicon size, product Tm, and amplification efficiencies of the standard PCR and qPCR reactions are shown in Supplementary Table 4.

Three primer couples (i.e., *Ov-wls*; *Ov-ESR16*; *Ov-C2CD2*, isoform X2) exhibited no or multiple amplification products in agarose gel and were excluded from subsequent analyses, while all other primer couples presented a single amplification product at the expected amplicon size (Supplementary Figure 3) and were therefore tested for RT-qPCR.

Among the remaining candidates, 59 primer pairs showed amplification efficiencies between 98% and 102%, while 19 did not fall within this range and were excluded from further analyses (Supplementary Table 4).

Ct values of the candidate reference genes for *O. vulgaris*

Supplementary Table 5. Ct values, presented as descriptive statistics (range, min and max Ct, mean, standard error and standard deviation) for the 59 candidate reference genes across all *O. vulgaris* samples using qRT-PCR data.

Gene Names	Range	Minimum	Maximum	Ct Mean	Std. Error	Std. Deviation
Ov-prrc1	7.431	25.398	32.828	27.348	0.172	1.626
Ov-gk5	5.965	26.096	32.062	28.411	0.136	1.320
Ov-CUL1	5.759	25.658	29.416	27.905	0.155	1.466
Ov-timm	6.597	25.897	32.494	28.334	0.154	1.454
Ov-wdr44	10.091	25.725	35.816	28.842	0.234	2.212
Ov-CHCHD7	6.212	27.059	33.271	29.540	0.164	1.544
Ov-tollip	11.897	21.619	33.516	24.401	0.247	2.331
Ov-Naa15	8.402	25.154	33.556	27.560	0.171	1.614
Ov-PTPN12	9.469	24.495	33.963	27.745	0.232	2.187
Ov-SUCLG2	7.281	25.994	33.275	28.399	0.182	1.717
Ov-syvn1	6.144	27.874	32.018	30.395	0.150	1.415
Ov-AP5Z1	9.358	25.239	34.598	29.064	0.247	2.334
Ov-Homer2	10.758	25.573	36.330	29.184	0.269	2.535
Ov-AGL	8.750	23.680	32.431	26.502	0.230	2.169
Ov-PIP4K2B	9.793	22.115	31.908	26.309	0.236	2.229
Ov-Snx20	8.006	25.557	33.563	29.406	0.216	2.034
Ov-Fam160a2	7.664	25.964	33.628	29.116	0.196	1.847
Ov-Snx25	11.126	24.852	35.977	28.703	0.254	2.396
Ov-WBP2	8.885	22.525	31.410	26.109	0.227	2.138
Ov-CSDE1	6.343	22.745	29.088	24.833	0.170	1.607
Ov-mts	10.554	22.617	33.171	26.156	0.259	2.443
Ov-Ltv1	5.240	26.069	29.309	28.171	0.110	1.037
Ov-BTBD17	4.904	26.236	31.140	28.004	0.117	1.104
Ov-rpf1	7.770	26.738	34.508	29.519	0.174	1.642
Ov-CG9286	8.757	25.291	34.048	28.236	0.178	1.677
Ov-EIF3M	4.348	24.704	29.052	26.600	0.118	1.109
Ov-NOB1	5.396	28.220	33.616	31.309	0.119	1.121
Ov-Dap3	6.999	26.185	33.184	29.056	0.161	1.517
Ov-slc25a40	7.256	25.819	30.075	28.192	0.164	1.550
Ov-ATPAF2	6.859	27.088	33.947	29.602	0.179	1.690

Gene Names	Range	Minimum	Maximum	Ct Mean	Std. Error	Std. Deviation
Ov-RIOK2	5.123	28.113	33.236	30.210	0.128	1.208
Ov-Dnaja3	11.524	25.750	37.273	28.341	0.235	2.217
Ov-EIF2A	6.138	25.339	29.477	27.709	0.153	1.441
Ov-usp10	6.700	26.130	32.831	28.665	0.157	1.485
Ov-CPIJ005834	5.945	26.165	32.110	28.802	0.150	1.413
Ov-Ppm1b	7.317	22.417	29.734	25.070	0.181	1.709
Ov-UBE2F	6.393	25.711	30.104	27.880	0.145	1.372
Ov-RAD23B	5.554	23.091	28.645	25.714	0.151	1.422
Ov-Abhd18	8.846	24.730	33.576	28.067	0.192	1.810
Ov-BTBD2	8.778	24.259	33.037	26.886	0.178	1.678
Ov-KCMF1	9.378	23.904	33.282	26.464	0.216	2.037
Ov-RNF7	8.021	25.390	33.411	27.698	0.173	1.633
Ov-Abi2	5.267	24.303	29.571	26.236	0.123	1.160
Ov-Rnd3	5.172	26.539	29.712	28.359	0.140	1.318
Ov-Vbp1	5.816	23.605	27.921	26.436	0.164	1.552
Ov-C2CD2	9.034	23.154	32.188	26.747	0.191	1.805
Ov-MRM2	8.272	24.477	32.750	27.222	0.175	1.651
Ov-Sdhd	5.825	23.752	29.577	26.140	0.154	1.455
Ov-UGP2	6.582	22.765	29.347	25.180	0.168	1.586
Ov-PCK1	10.502	25.373	35.875	28.721	0.191	1.802
Ov-TUBG1_FR	8.206	28.538	36.743	31.310	0.163	1.541
Ov-Rps27a_FR	6.789	22.158	28.948	24.849	0.154	1.448
Ov-MRPS5_FR	7.047	25.772	32.819	28.491	0.171	1.618
Ov-Tuba1a	8.349	18.168	26.517	21.626	0.205	1.938
Ov-Rpl6	6.950	21.847	28.796	24.148	0.150	1.411
Ov-TUBG1_F1R1	8.744	27.717	36.461	30.965	0.202	1.906
Ov-RpL23	6.946	23.217	30.164	26.953	0.138	1.306
Ov-MRPS5_F1R1	6.213	26.381	32.594	29.046	0.142	1.339
Ov-RPS18	6.102	22.100	28.202	24.789	0.127	1.201

Supplementary Figure 4. Box plots of the raw Ct values for the ten best reference genes (RGs) resulted after RefFinder for each of three groups of tissues considered in adult *Octopus vulgaris*: Nervous, Allex and Adult. A total of 19 RGs are shown here, since some of the genes are shared among the groups. Box plots are showed for all tissues per gene; tissues depicted in red do not belong to the considered group.

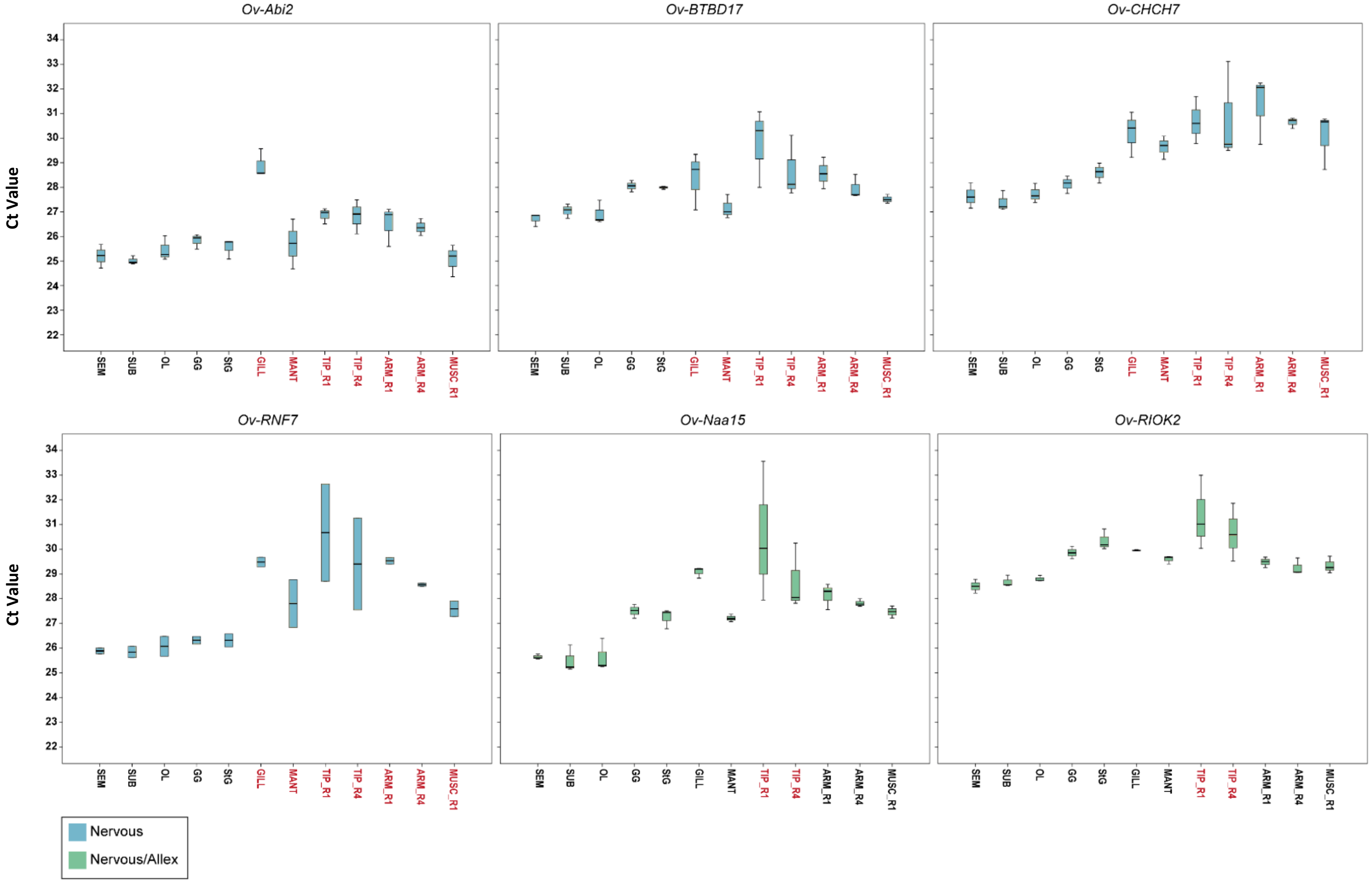

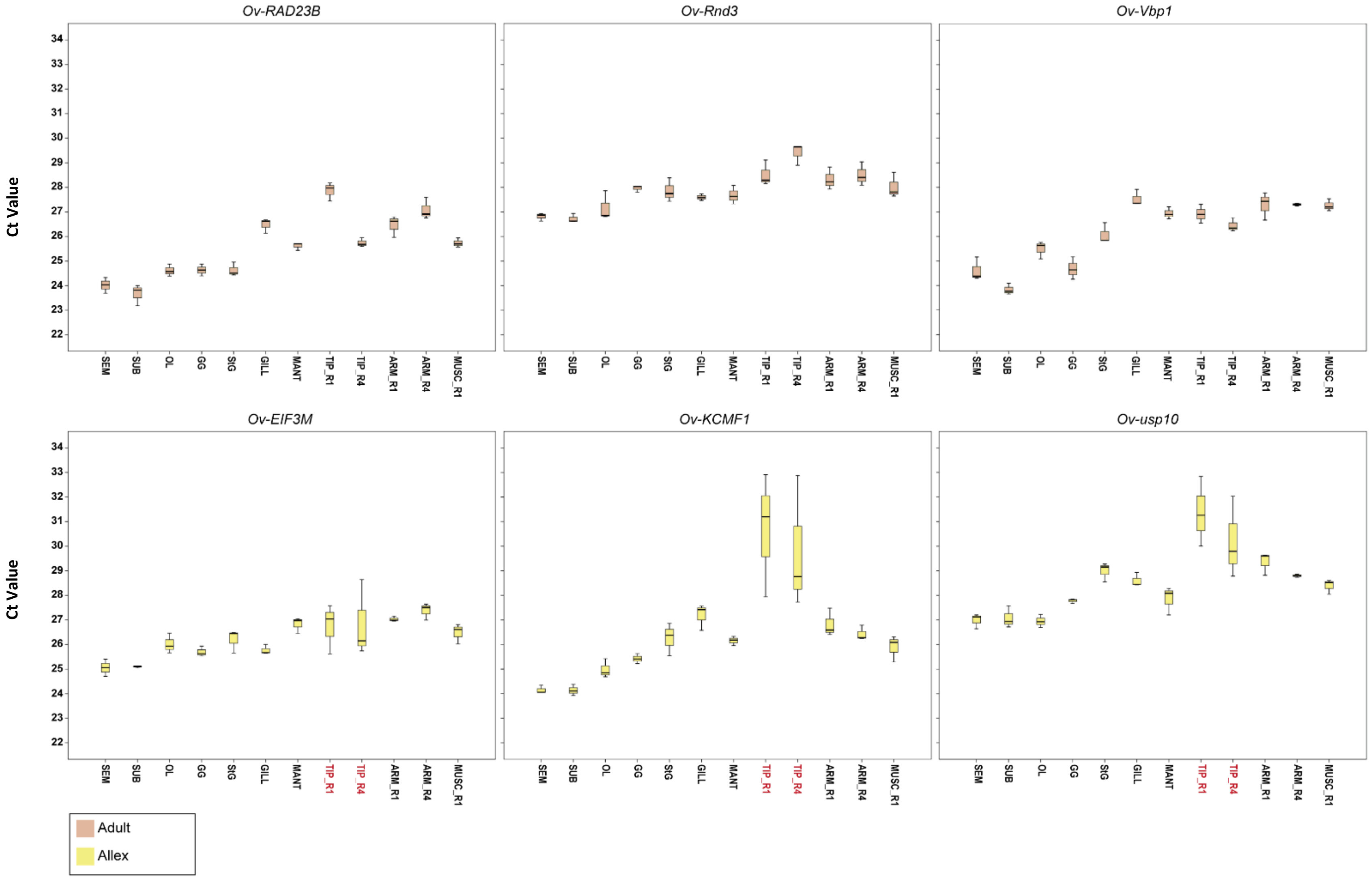

Ov-RAD23B
Ov-Rnd3
Ov-Vbp1
Ov-EIF3M
Ov-KCMF1
Ov-usp10
Ct Value
SEM
SUB
OL
GG
SIG
GILL
MANT
TIP_R1
TIP_R4
ARM_R1
ARM_R4
MUSC_R1
Adult
Allex

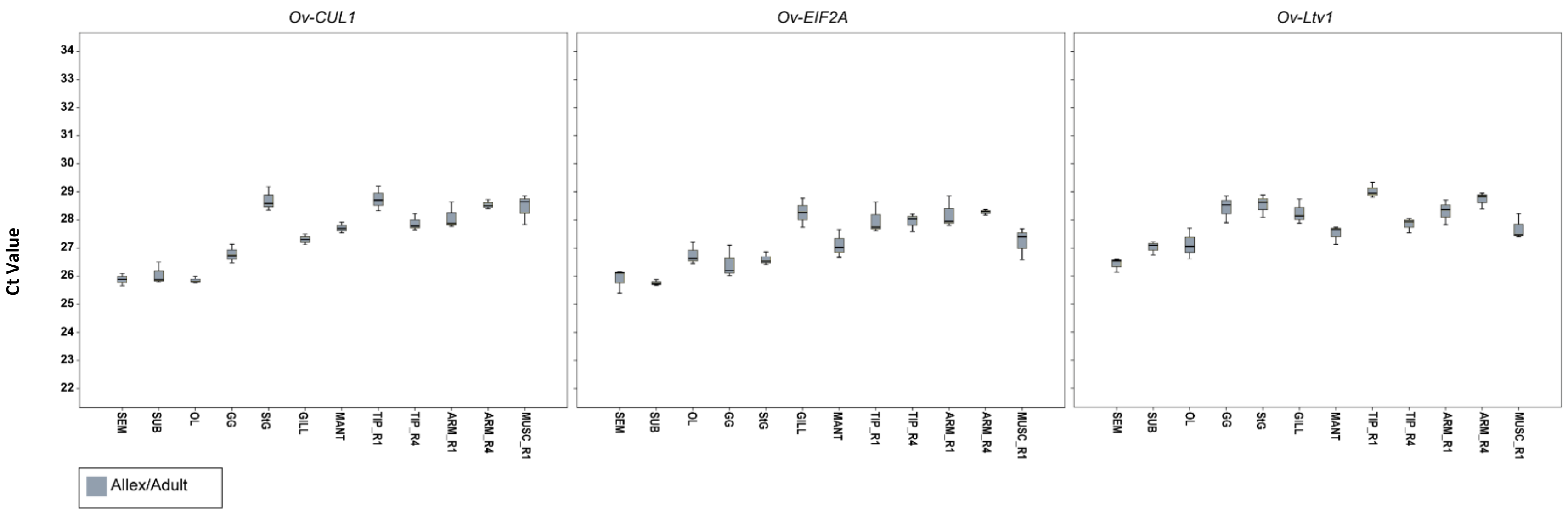
Ov-CUL1
Ov-EIF2A
Ov-Ltv1
Ct Value
34
33
32
31
30
29
28
27
26
25
24
23
22
SEM
SUB
OL
GG
SIG
GILL
MANT
TIP_R1
TIP_R4
ARM_R1
ARM_R4
MUSC_R1
Allex/Adult

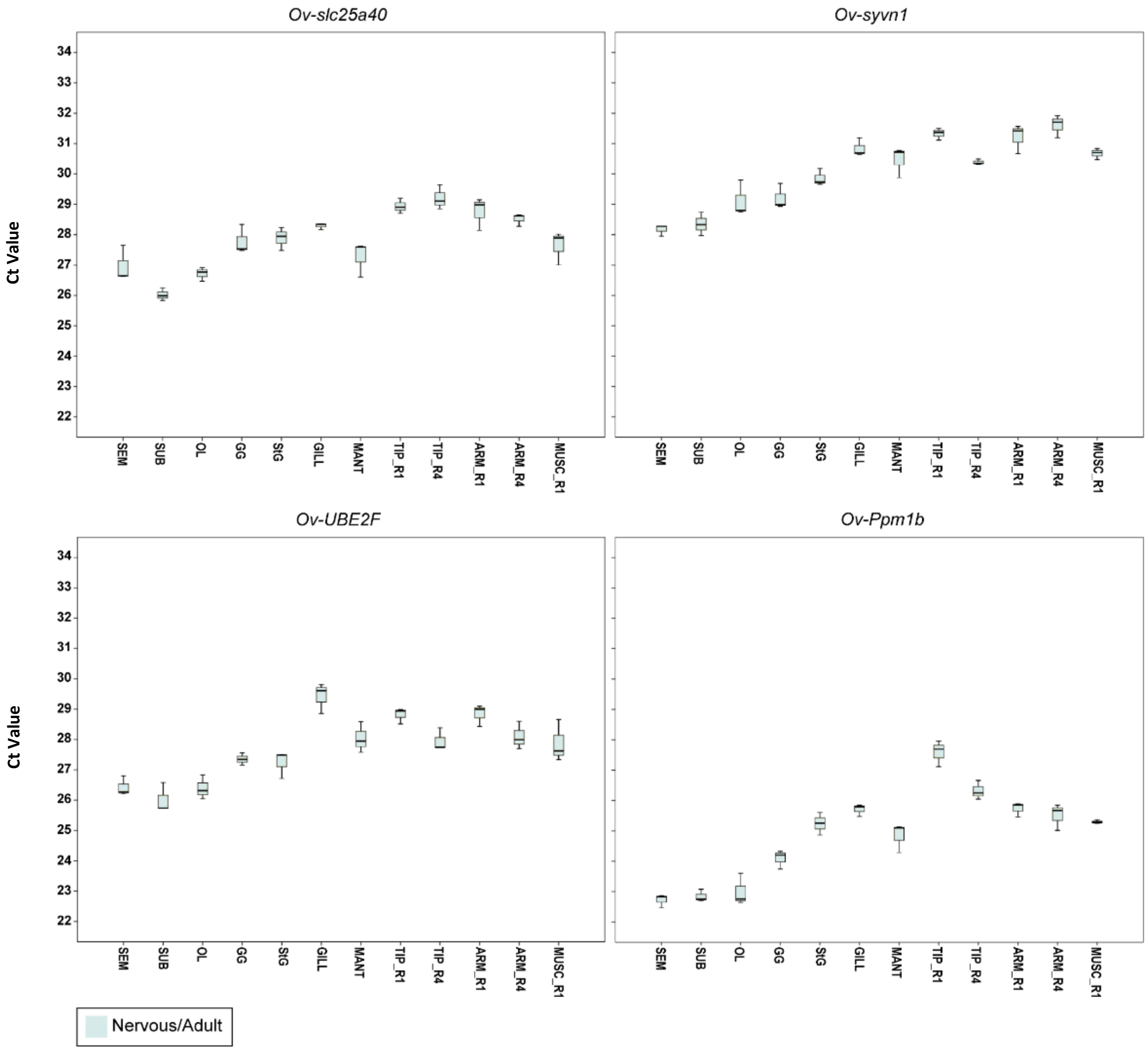
Ov-slc25a40
Ov-syvn1
Ov-UBE2F
Ov-Ppm1b
Ct Value
34
33
32
31
30
29
28
27
26
25
24
23
22
SEM
SUB
OL
GG
StG
GILL
MANT
TIP_R1
TIP_R4
ARM_R1
ARM_R4
MUSC_R1
Nervous/Adult

Validation of the *in silico* gene expression profiles

The genes *Ov-CHCHD7*, *Ov-UBE2F*, and *Ov-Naa15* were identified as suitable reference genes both by the *in silico* analysis for the **Nervous** group (Table 1 and Supplementary Figure 1) and by the validation trough RT-qPCRs for group **Nervous** (Table 2). *Ov-UBE2F*, *Ov-Ltv1*, *Ov-EIF2A*, *Ov-slc25a40*, *Ov-RIOK2*, *Ov-Dap3*, and *Ov-Ppm1b* were identified as suitable candidates reference genes by RNA-seq for tissues included in the **Adult** group (Table 1 and Supplementary Figure 1) and then confirmed by RT-qPCR experiments in **Adult** (Table 2). *Ov-UBE2F* was the only candidate gene identified by RNA-seq data for the **ARM**, **ADULT** and **Nervous** (Table 1 and Supplementary Figure 1) that resulted to be suitable as reference also in all the three groups analysed by RT-qPCR (Table 2).

Gastric ganglion (GG) was considered as the reference 'tissue'. A combination of the two most stable (*Ov-RNF7* and *Ov-RIOK2*), the most stable (*Ov-RNF7*), and the least stable (most unstable; *Ov-Rps27a-FR*) RGs was used to normalize the expression of the target genes.

Selected target genes for validation

We selected nine genes as target candidates and assessed their expression in brain tissues. The genes are: *Ov-Naa15*, *Ov-Ltv1*, *Ov-CG9286*, *Ov-EIF3M*, *Ov-NOB1*, *Ov-CSDE1*, *Ov-Abi2*, *Ov-Homer2*, *Ov-Snx20*.

The N-alpha-acetyltransferase 15, NatA auxiliary subunit (Naa15) appears involved in neuronal growth and development in mammals (Lee et al., 2018), with gene disrupting variants linked to disabilities and autism spectrum disorder in humans (Cheng et al., 2018). Missense variants of the human Naa15 ortholog in *Caenorhabditis elegans* (usually involved in neuronal signalling and cytoskeleton functions) also induce morphological alterations and locomotion defects (Wong et al., 2019).

Members of the Homer family (Shiraishi-Yamaguchi and Furuichi, 2007) acts as scaffolding proteins and are mainly localized at the postsynaptic level in the mammalian nervous system where they act as adaptor proteins. They appear to have a role in synaptogenesis, signal

transduction, and receptor trafficking. In mammals three members of the Homer family are known, and have been identified also in lower vertebrates, with role in axon guidance in *Xenopus laevis* tadpoles (Foa et al., 2001). Among invertebrates ortholog has been found in *Drosophila melanogaster* (Diagana et al., 2002) and expressed in various tissues, but highly enriched in the nervous system. Similarly to what occurs in mammals, *Drosophila Homer* is found in dendrites and the endoplasmic reticulum of the nervous system, and found involved in the neural control of locomotory patterns and behavioural plasticity.

Sorting nexin-20 and Sorting nexin-25 belong to a protein family involved in the functional organization of the endosomal network, regulating cell trafficking and signaling processes. Sorting nexin proteins have been linked to inflammatory response elicited by pathogens and to the amyloid beta (Aβ) accumulation in Alzheimer's disease in humans (Cullen, 2008; Teasdale and Collins, 2011).

Abl interactor 2 is an adaptor protein involved in the regulation of cytoskeletal dynamics, cell migration and morphogenesis and dentritic spine density. In mice gene knockout determines lens defective development and impaired learning and memory (Grove et al., 2004). In *D. melanogaster* loss of function mutants enabled to identify a role for Abl in the central nervous system development, because of its role in axonal guidance and synaptic formation (Lin et al., 2009).

CSDE1 (Cold shock domain-containing protein E1) is an RNA binding protein regulating RNAs translation in several processes including cell cycle, apoptosis and cell differentiation (e.g., Kakumani et al., 2020; Smith et al., 2022). It has been associated to autisms disorder, and has a crucial role in embryonic development and reprogramming (e.g., Guo et al., 2020). Among invertebrates the cold shock domain-containing protein has been identified in various organisms and found to play role in temperature tolerance and other stressors, to mention some (Artigaud et al., 2015; Si et al., 2022).

EIF3M (Eukaryotic translation initiation factor 3 subunit M) is an evolutionary conserved component of the eukaryotic translation initiation factor 3 (eIF-3) complex. It is involved in regulating mRNA translation and differential gene expression (e.g., Kim et al., 2007). In invertebrates has been shown to play different roles linked with its main function (Rylee et al., 2022; Ameijeiras et al., 2023).

Protein BCCIP (*CG9286* in *D. melanogaster)* plays a critical role in interphase, organizing and stabilizing the spindle pole during mitosis. Alterations in its expression are linked to mitotic

defects and altered cell differentiation during embryogenesis (Huhn et al., 2017). BCCIP deficient mice show reduced proliferation, increased apoptosis and consequent neurogenesis brain defect, suggesting for a role in mediating neural progenitor proliferation in neural development (Huang et al., 2012). BCCIP appears to be a key element for the construction of complex brain architecture.

LTV1 is a conserved protein required for ribosomal biogenesis (Loar et al., 2004). In fruit flies, the correct protein synthesis driven by LTV1 is essential for normal cell growth and survival with increased mortality in LTV1 mutant (Kim et al., 2015).

Rps27a (Ubiquitin-40S ribosomal protein S27a) is commonly used as a reference gene and plays a number of important functions in gene regulation (e.g., Genuth and Barna, 2018; Feng et al., 2019).

References

Adema, C.M. (2021). Sticky problems: extraction of nucleic acids from molluscs. *Philosophical Transactions of the Royal Society B: Biological Sciences* 376(1825)**,** 20200162. doi: doi:10.1098/rstb.2020.0162.

Albertin, C.B., Bonnaud, L., Brown, C.T., Crookes-Goodson, W.J., da Fonseca, R.R., Di Cristo, C., Dilkes, B.P., Edsinger-Gonzales, E., Freeman, R.M., Jr., Hanlon, R.T., Koenig, K.M., Lindgren, A.R., Martindale, M.Q., Minx, P., Moroz, L.L., Nodl, M.T., Nyholm, S.V., Ogura, A., Pungor, J.R., Rosenthal, J.J., Schwarz, E.M., Shigeno, S., Strugnell, J.M., Wollesen, T., Zhang, G., and Ragsdale, C.W. (2012). Cephalopod genomics: A plan of strategies and organization. *Stand. Genomic Sci.* 7**,** 175-188.

Albertin, C.B., Medina-Ruiz, S., Mitros, T., Schmidbaur, H., Sanchez, G., Wang, Z.Y., Grimwood, J., Rosenthal, J.J.C., Ragsdale, C.W., Simakov, O., and Rokhsar, D.S. (2022). Genome and transcriptome mechanisms driving cephalopod evolution. *Nature Communications* 13(1)**,** 2427. doi: 10.1038/s41467-022-29748-w.

Albertin, C.B., and Simakov, O. (2020). Cephalopod Biology: At the Intersection Between Genomic and Organismal Novelties. *Annual Review of Animal Biosciences* 8(1)**,** 71-90. doi: 10.1146/annurev-animal-021419-083609.

Albertin, C.B., Simakov, O., Mitros, T., Wang, Z.Y., Pungor, J.R., Edsinger-Gonzales, E., Brenner, S., Ragsdale, C.W., and Rokhsar, D.S. (2015). The octopus genome and the evolution of cephalopod neural and morphological novelties. *Nature* 524(7564)**,** 220-224.

Ameijeiras, P., Capriotti, N., Ons, S., Oliveira, P.L., and Sterkel, M. (2023). eIF3 subunit M regulates blood meal digestion in *Rhodnius prolixus* affecting ecdysis, reproduction and survival. *Insect Science* In Press(online first). doi: 10.1111/1744-7917.13174.

Andersen, C.L., Jensen, J.L., and Ørntoft, T.F. (2004). Normalization of Real-Time Quantitative Reverse Transcription-PCR Data: A Model-Based Variance Estimation Approach to Identify Genes Suited for Normalization, Applied to Bladder and Colon Cancer Data Sets. *Cancer Research* 64(15)**,** 5245-5250. doi: 10.1158/0008-5472.Can-04-0496.

Andrews, P.L.R., Darmaillacq, A.S., Dennison, N., Gleadall, I.G., Hawkins, P., Messenger, J.B., Osorio, D., Smith, V.J., and Smith, J.A. (2013). The identification and management of pain, suffering and distress in cephalopods, including anaesthesia, analgesia and humane killing. *J. Exp. Mar. Biol. Ecol.* 447**,** 46-64.

Artigaud, S., Richard, J., Thorne, M.A.S., Lavaud, R., Flye-Sainte-Marie, J., Jean, F., Peck, L.S., Clark, M.S., and Pichereau, V. (2015). Deciphering the molecular adaptation of the king scallop (*Pecten maximus*) to heat stress using transcriptomics and proteomics. *BMC Genomics* 16(1)**,** 988. doi: 10.1186/s12864-015-2132-x.

Baldascino, E., Di Cristina, G., Tedesco, P., Hobbs, C., Shaw, T.J., Ponte, G., and Andrews, P.L.R. (2017). The Gastric Ganglion of *Octopus vulgaris*: Preliminary Characterization of Gene- and Putative Neurochemical-Complexity, and the Effect of *Aggregata octopiana* Digestive Tract Infection on Gene Expression. *Frontiers in Physiology* 8(1001). doi: 10.3389/fphys.2017.01001.

Belcaid, M., Casaburi, G., McAnulty, S.J., Schmidbaur, H., Suria, A.M., Moriano-Gutierrez, S., Pankey, M.S., Oakley, T.H., Kremer, N., Koch, E.J., Collins, A.J., Nguyen, H., Lek, S., Goncharenko-Foster, I., Minx, P., Sodergren, E., Weinstock, G., Rokhsar, D.S., McFall-Ngai, M., Simakov, O., Foster, J.S., and Nyholm, S.V. (2019). Symbiotic organs shaped by distinct modes of genome evolution in cephalopods. *Proceedings of the National Academy of Sciences* 116(8)**,** 3030-3035. doi: 10.1073/pnas.1817322116.

Benoist, L., Corre, E., Bernay, B., Henry, J., and Zatylny-Gaudin, C. (2020). -Omic Analysis of the *Sepia officinalis* White Body: New Insights into Multifunctionality and Haematopoiesis Regulation. *Journal of Proteome Research* 19(8)**,** 3072-3087. doi: 10.1021/acs.jproteome.0c00100.

Butler-Struben, H.M., Brophy, S.M., Johnson, N.A., and Crook, R.J. (2018). In vivo recording of neural and behavioral correlates of anesthesia induction, reversal, and euthanasia in cephalopod molluscs. *Frontiers in Physiology* 9**,** 109.

Caruana, N.J., Cooke, I.R., Faou, P., Finn, J., Hall, N.E., Norman, M., Pineda, S.S., and Strugnell, J.M. (2016). A combined proteomic and transcriptomic analysis of slime secreted by the southern bottletail squid, *Sepiadarium austrinum* (Cephalopoda). *Journal of Proteomics* 148**,** 170-182. doi: https://doi.org/10.1016/j.jprot.2016.07.026.

Castellanos-Martínez, S., Arteta, D., Catarino, S., and Gestal, C. (2014). De Novo Transcriptome Sequencing of the *Octopus vulgaris* Hemocytes Using Illumina RNA-Seq Technology: Response to the Infection by the Gastrointestinal Parasite *Aggregata octopiana*. *PLOS ONE* 9(10)**,** e107873. doi: 10.1371/journal.pone.0107873.

Chapko, M.K., Grossbeck, M.L., Hansen, R.L., Maher, T.D., Middleton, R.S., and Simpson, R.W. (1962). *Devilfish. A Practical Guide to the Dissection of Octopus.* Wayne Senior High School.

Cheng, H., Dharmadhikari, A.V., Varland, S., Ma, N., Domingo, D., Kleyner, R., Rope, A.F., Yoon, M., Stray-Pedersen, A., Posey, J.E., Crews, S.R., Eldomery, M.K., Akdemir, Z.C., Lewis, A.M., Sutton, V.R., Rosenfeld, J.A., Conboy, E., Agre, K., Xia, F., Walkiewicz, M., Longoni, M., High, F.A., van Slegtenhorst, M.A., Mancini, G.M.S., Finnila, C.R., van Haeringen, A., den Hollander, N., Ruivenkamp, C., Naidu, S., Mahida, S., Palmer, E.E., Murray, L., Lim, D., Jayakar, P., Parker, M.J., Giusto, S., Stracuzzi, E., Romano, C., Beighley, J.S., Bernier, R.A., Küry, S., Nizon, M., Corbett, M.A., Shaw, M., Gardner, A., Barnett, C., Armstrong, R., Kassahn, K.S., Van Dijck, A., Vandeweyer, G., Kleefstra, T., Schieving, J., Jongmans, M.J., de Vries, B.B.A., Pfundt, R., Kerr, B., Rojas, S.K., Boycott, K.M., Person, R., Willaert, R., Eichler, E.E., Kooy, R.F., Yang, Y., Wu, J.C.,

Lupski, J.R., Arnesen, T., Cooper, G.M., Chung, W.K., Gecz, J., Stessman, H.A.F., Meng, L., and Lyon, G.J. (2018). Truncating Variants in NAA15 Are Associated with Variable Levels of Intellectual Disability, Autism Spectrum Disorder, and Congenital Anomalies. *The American Journal of Human Genetics* 102(5), 985-994. doi: https://doi.org/10.1016/j.ajhg.2018.03.004.

Collins, A., Schleicher, T., Rader, B., and Nyholm, S. (2012). Understanding the Role of Host Hemocytes in a Squid/Vibrio Symbiosis Using Transcriptomics and Proteomics. *Frontiers in Immunology* 3. doi: 10.3389/fimmu.2012.00091.

Cullen, P.J. (2008). Endosomal sorting and signalling: an emerging role for sorting nexins. *Nature Reviews Molecular Cell Biology* 9(7), 574-582. doi: 10.1038/nrm2427.

da Fonseca, R.R., Couto, A., Machado, A.M., Brejova, B., Albertin, C.B., Silva, F., Gardner, P., Baril, T., Hayward, A., Campos, A., Ribeiro, Â.M., Barrio-Hernandez, I., Hoving, H.-J., Tafur-Jimenez, R., Chu, C., Frazão, B., Petersen, B., Peñaloza, F., Musacchia, F., Alexander, G.C., Jr, Osório, H., Winkelmann, I., Simakov, O., Rasmussen, S., Rahman, M.Z., Pisani, D., Vinther, J., Jarvis, E., Zhang, G., Strugnell, J.M., Castro, L.F.C., Fedrigo, O., Patricio, M., Li, Q., Rocha, S., Antunes, A., Wu, Y., Ma, B., Sanges, R., Vinar, T., Blagoev, B., Sicheritz-Ponten, T., Nielsen, R., and Gilbert, M.T.P. (2020). A draft genome sequence of the elusive giant squid, Architeuthis dux. *GigaScience* 9(1). doi: 10.1093/gigascience/giz152.

Diagana, T.T., Thomas, U., Prokopenko, S.N., Xiao, B., Worley, P.F., and Thomas, J.B. (2002). Mutation of *Drosophila homer* Disrupts Control of Locomotor Activity and Behavioral Plasticity. *The Journal of Neuroscience* 22(2), 428-436. doi: 10.1523/jneurosci.22-02-00428.2002.

Feng, H., Guo, X., Sun, H., Zhang, S., Xi, J., Yin, J., Cao, Y., and Li, K. (2019). Flight muscles degenerate by programmed cell death after migration in the wheat aphid, *Sitobion avenae*. *BMC Research Notes* 12(1), 672. doi: 10.1186/s13104-019-4708-z.

Fiorito, G., Affuso, A., Basil, J., Cole, A., de Girolamo, P., D'Angelo, L., Dickel, L., Gestal, C., Grasso, F., Kuba, M., Mark, F., Melillo, D., Osorio, D., Perkins, K., Ponte, G., Shashar, N., Smith, D., Smith, J., and Andrews, P.L. (2015). Guidelines for the Care and Welfare of Cephalopods in Research - A consensus based on an initiative by CephRes, FELASA and the Boyd Group. *Lab. Anim.* 49(2 Suppl), 1-90.

Foa, L., Rajan, I., Haas, K., Wu, G.-Y., Brakeman, P., Worley, P., and Cline, H. (2001). The scaffold protein, Homer1b/c, regulates axon pathfinding in the central nervous system *in vivo*. *Nature Neuroscience* 4(5), 499-506. doi: 10.1038/87447.

García-Fernández, P., Castellanos-Martínez, S., Iglesias, J., Otero, J.J., and Gestal, C. (2016). Selection of reliable reference genes for RT-qPCR studies in *Octopus vulgaris* paralarvae during development and immune-stimulation. *Journal of Invertebrate Pathology* 138, 57-62. doi: https://doi.org/10.1016/j.jip.2016.06.003.

García-Fernández, P., Prado-Alvarez, M., Nande, M., Garcia de la serrana, D., Perales-Raya, C., Almansa, E., Varó, I., and Gestal, C. (2019). Global impact of diet and temperature

over aquaculture of *Octopus vulgaris* paralarvae from a transcriptomic approach. *Scientific Reports* 9(1), 10312. doi: 10.1038/s41598-019-46492-2.

Genuth, N.R., and Barna, M. (2018). The Discovery of Ribosome Heterogeneity and Its Implications for Gene Regulation and Organismal Life. *Molecular Cell* 71(3), 364-374. doi: 10.1016/j.molcel.2018.07.018.

Gleadall, I.G. (2013). The effects of prospective anaesthetic substances on cephalopods: summary of original data and a brief review of studies over the last two decades. *J. Exp. Mar. Biol. Ecol* 447, 23-30.

Grimaldi, A.M., Agnisola, C., and Fiorito, G. (2007). Using ultrasound to estimate brain size in the cephalopod *Octopus vulgaris* Cuvier in vivo. *Brain Research* 1183, 66-73.

Grove, M., Demyanenko, G., Echarri, A., Zipfel, P.A., Quiroz, M.E., Rodriguiz, R.M., Playford, M., Martensen, S.A., Robinson, M.R., Wetsel, W.C., Maness, P.F., and Pendergast, A.M. (2004). Abi2-Deficient Mice Exhibit Defective Cell Migration, Aberrant Dendritic Spine Morphogenesis, and Deficits in Learning and Memory. *Molecular and Cellular Biology* 24(24), 10905-10922. doi: doi:10.1128/MCB.24.24.10905-10922.2004.

Guerra, Á. (2019). "Functional Anatomy: Macroscopic Anatomy and Post-mortem Examination," in *Handbook of Pathogens and Diseases in Cephalopods*. Springer), p. 11-38.

Guo, A.-X., Cui, J.-J., Wang, L.-Y., and Yin, J.-Y. (2020). The role of CSDE1 in translational reprogramming and human diseases. *Cell Communication and Signaling* 18(1), 14. doi: 10.1186/s12964-019-0496-2.

Heath-Heckman, E., and Nishiguchi, M.K. (2021). Leveraging Short-Read Sequencing to Explore the Genomics of Sepiolid Squid. *Integrative and Comparative Biology* 61(5), 1753-1761. doi: 10.1093/icb/icab152.

Huang, Y.-Y., Lu, H., Liu, S., Droz-Rosario, R., and Shen, Z. (2012). Requirement of Mouse BCCIP for Neural Development and Progenitor Proliferation. *PLOS ONE* 7(1), e30638. doi: 10.1371/journal.pone.0030638.

Huhn, S.C., Liu, J., Ye, C., Lu, H., Jiang, X., Feng, X., Ganesan, S., White, E., and Shen, Z. (2017). Regulation of spindle integrity and mitotic fidelity by BCCIP. *Oncogene* 36(33), 4750-4766. doi: 10.1038/onc.2017.92.

Imperadore, P. (2017). *Nerve regeneration in the cephalopod mollusc Octopus vulgaris: a journey into morphological, cellular and molecular changes including epigenetic modifications.* PhD PhD, Università della Calabria.

Kakumani, P.K., Harvey, L.-M., Houle, F., Guitart, T., Gebauer, F., and Simard, M.J. (2020). CSDE1 controls gene expression through the miRNA-mediated decay machinery. *Life Science Alliance* 3(4), e201900632. doi: 10.26508/lsa.201900632.

Kim, B.-H., Cai, X., Vaughn, J.N., and von Arnim, A.G. (2007). On the functions of the h subunit of eukaryotic initiation factor 3 in late stages of translation initiation. *Genome Biology* 8(4), R60. doi: 10.1186/gb-2007-8-4-r60.

Kim, B.-M., Kang, S., Ahn, D.-H., Jung, S.-H., Rhee, H., Yoo, J.S., Lee, J.-E., Lee, S., Han, Y.-H., Ryu, K.-B., Cho, S.-J., Park, H., and An, H.S. (2018). The genome of common long-arm octopus *Octopus minor*. *GigaScience* 7, giy119. doi: 10.1093/gigascience/giy119.

Kim, W., Kim, H.D., Jung, Y., Kim, J., and Chung, J. (2015). *Drosophila* Low Temperature Viability Protein 1 (LTV1) Is Required for Ribosome Biogenesis and Cell Growth Downstream of *Drosophila* Myc (dMyc). *Journal of Biological Chemistry* 290(21), 13591-13604. doi: 10.1074/jbc.M114.607036.

Lee, M.-N., Kweon, H.Y., and Oh, G.T. (2018). N-α-acetyltransferase 10 (NAA10) in development: the role of NAA10. *Experimental & Molecular Medicine* 50(7), 1-11. doi: 10.1038/s12276-018-0105-2.

Li, F., Bian, L., Ge, J., Han, F., Liu, Z., Li, X., Liu, Y., Lin, Z., Shi, H., Liu, C., Chang, Q., Lu, B., Zhang, S., Hu, J., Xu, D., Shao, C., and Chen, S. (2020). Chromosome-level genome assembly of the East Asian common octopus (*Octopus sinensis*) using PacBio sequencing and Hi-C technology. *Molecular Ecology Resources* 20(6), 1572-1582. doi: https://doi.org/10.1111/1755-0998.13216.

Lin, T.-Y., Huang, C.-H., Kao, H.-H., Liou, G.-G., Yeh, S.-R., Cheng, C.-M., Chen, M.-H., Pan, R.-L., and Juang, J.-L. (2009). Abi plays an opposing role to Abl in Drosophila axonogenesis and synaptogenesis. *Development* 136(18), 3099-3107. doi: 10.1242/dev.033324.

Loar, J.W., Seiser, R.M., Sundberg, A.E., Sagerson, H.J., Ilias, N., Zobel-Thropp, P., Craig, E.A., and Lycan, D.E. (2004). Genetic and Biochemical Interactions Among Yar1, Ltv1 and RpS3 Define Novel Links Between Environmental Stress and Ribosome Biogenesis in *Saccharomyces cerevisiae*. *Genetics* 168(4), 1877-1889. doi: 10.1534/genetics.104.032656.

Lü, Z., Liu, W., Liu, L., Shi, H., Ping, H., Wang, T., Chi, C., Wu, C., Chen, C.-H., Shen, K.-N., and Hsiao, C.-D. (2016). De novo assembly and comparison of the ovarian transcriptomes of the common Chinese cuttlefish (*Sepiella japonica*) with different gonadal development. *Genomics Data* 7, 155-158. doi: https://doi.org/10.1016/j.gdata.2015.12.011.

Mestdagh, P., Van Vlierberghe, P., De Weer, A., Muth, D., Westermann, F., Speleman, F., and Vandesompele, J. (2009). A novel and universal method for microRNA RT-qPCR data normalization. *Genome Biology* 10(6), R64. doi: 10.1186/gb-2009-10-6-r64.

Natsidis, P., Schiffer, P.H., Salvador-Martínez, I., and Telford, M.J. (2019). Computational discovery of hidden breaks in 28S ribosomal RNAs across eukaryotes and consequences for RNA Integrity Numbers. *Scientific Reports* 9(1), 19477. doi: 10.1038/s41598-019-55573-1.

Pankey, M.S., Minin, V.N., Imholte, G.C., Suchard, M.A., and Oakley, T.H. (2014). Predictable transcriptome evolution in the convergent and complex bioluminescent organs of squid. *Proceedings of the National Academy of Sciences* 111(44), E4736-E4742. doi: doi:10.1073/pnas.1416574111.

Petrosino, G. (2015). *The transcriptional landscape of the nervous system of Octopus vulgaris.* PhD Thesis, Università degli Studi di Napoli Federico II.

Petrosino, G., Ponte, G., Volpe, M., Zarrella, I., Ansaloni, F., Langella, C., Di Cristina, G., Finaurini, S., Russo, M.T., Basu, S., Musacchia, F., Ristoratore, F., Pavlinic, D., Benes, V., Ferrante, M.I., Albertin, C., Simakov, O., Gustincich, S., Fiorito, G., and Sanges, R. (2022). Identification of LINE retrotransposons and long non-coding RNAs expressed in the octopus brain. *BMC Biology* 20(1), 116. doi: 10.1186/s12915-022-01303-5.

Pfaffl, M.W., Tichopad, A., Prgomet, C., and Neuvians, T.P. (2004). Determination of stable housekeeping genes, differentially regulated target genes and sample integrity: BestKeeper – Excel-based tool using pair-wise correlations. *Biotechnology Letters* 26(6), 509-515. doi: 10.1023/B:BILE.0000019559.84305.47.

Ponte, G., Droscher, A., and Fiorito, G. (2013). Fostering cephalopod biology research: past and current trends and topics. *Invertebrate Neuroscience* 13(1), 1-9. doi: 10.1007/s10158-013-0156-y.

Prado-Álvarez, M., Dios, S., García-Fernández, P., Tur, R., Hachero-Cruzado, I., Domingues, P., Almansa, E., Varó, I., and Gestal, C. (2022). De novo transcriptome reconstruction in aquacultured early life stages of the cephalopod *Octopus vulgaris*. *Scientific Data* 9(1), 609. doi: 10.1038/s41597-022-01735-2.

Ritschard, E.A., Whitelaw, B., Albertin, C.B., Cooke, I.R., Strugnell, J.M., and Simakov, O. (2019). Coupled Genomic Evolutionary Histories as Signatures of Organismal Innovations in Cephalopods. *BioEssays* 41(12), 1900073. doi: 10.1002/bies.201900073.

Rylee, J., Mahato, S., Aldrich, J., Bergh, E., Sizemore, B., Feder, L.E., Grega, S., Helms, K., Maar, M., Britt, S.G., and Zelhof, A.C. (2022). A TRiP RNAi screen to identify molecules necessary for *Drosophila* photoreceptor differentiation. *G3 Genes|Genomes|Genetics* 12(11). doi: 10.1093/g3journal/jkac257.

Salazar, K.A., Joffe, N.R., Dinguirard, N., Houde, P., and Castillo, M.G. (2015). Transcriptome Analysis of the White Body of the Squid *Euprymna tasmanica* with Emphasis on Immune and Hematopoietic Gene Discovery. *PLOS ONE* 10(3), e0119949. doi: 10.1371/journal.pone.0119949.

Schmidbaur, H., Kawaguchi, A., Clarence, T., Fu, X., Hoang, O.P., Zimmermann, B., Ritschard, E.A., Weissenbacher, A., Foster, J.S., Nyholm, S.V., Bates, P.A., Albertin, C.B., Tanaka, E., and Simakov, O. (2022). Emergence of novel cephalopod gene regulation and expression through large-scale genome reorganization. *Nature Communications* 13(1), 2172. doi: 10.1038/s41467-022-29694-7.

Shiraishi-Yamaguchi, Y., and Furuichi, T. (2007). The Homer family proteins. *Genome Biology* 8(2)**,** 206. doi: 10.1186/gb-2007-8-2-206.

Si, M.-R., Li, Y.-D., Jiang, S.-G., Yang, Q.-B., Jiang, S., Yang, L.-S., Huang, J.-H., Chen, X., and Zhou, F.-L. (2022). A CSDE1/Unr gene from *Penaeus monodon*: Molecular characterization, expression and association with tolerance to low salt stress. *Aquaculture* 561**,** 738660. doi: https://doi.org/10.1016/j.aquaculture.2022.738660.

Sirakov, M., Zarrella, I., Borra, M., Rizzo, F., Biffali, E., Arnone, M.I., and Fiorito, G. (2009). Selection and validation of a set of reliable reference genes for quantitative RT-PCR studies in the brain of the Cephalopod Mollusc Octopus vulgaris. *BMC Molecular Biology* 10(1)**,** 70.

Smith, G.A., Padmanabhan, A., Lau, B.H., Pampana, A., Li, L., Lee, C.Y., Pelonero, A., Nishino, T., Sadagopan, N., Xia, V.Q., Jain, R., Natarajan, P., Wu, R.S., Black, B.L., Srivastava, D., Shokat, K.M., and Chorba, J.S. (2022). Cold shock domain-containing protein E1 is a posttranscriptional regulator of the LDL receptor. *Science Translational Medicine* 14(662)**,** eabj8670. doi: doi:10.1126/scitranslmed.abj8670.

Song, W., Li, R., Zhao, Y., Migaud, H., Wang, C., and Bekaert, M. (2021). Pharaoh Cuttlefish, *Sepia pharaonis*, Genome Reveals Unique Reflectin Camouflage Gene Set. *Frontiers in Marine Science* 8. doi: 10.3389/fmars.2021.639670.

Sousounis, K., Ogura, A., and Tsonis, P.A. (2013). Transcriptome Analysis of Nautilus and Pygmy Squid Developing Eye Provides Insights in Lens and Eye Evolution. *PLOS ONE* 8(10)**,** e78054. doi: 10.1371/journal.pone.0078054.

Sun, Y., Yao, C., Zhu, Y., Wang, Y., and Zhang, Z. (2022). Metabolism response of fasting in *Octopus sinensis* paralarvae revealed by RNA-seq. *Aquaculture* 550**,** 737859. doi: https://doi.org/10.1016/j.aquaculture.2021.737859.

Teasdale, Rohan D., and Collins, Brett M. (2011). Insights into the PX (phox-homology) domain and SNX (sorting nexin) protein families: structures, functions and roles in disease. *Biochemical Journal* 441(1)**,** 39-59. doi: 10.1042/bj20111226.

Tian, K., Lou, F., Gao, T., Zhou, Y., Miao, Z., and Han, Z. (2018). De novo assembly and annotation of the whole transcriptome of *Sepiella maindroni*. *Marine Genomics* 38**,** 13-16. doi: https://doi.org/10.1016/j.margen.2017.06.004.

Vandesompele, J., De Preter, K., Pattyn, F., Poppe, B., Van Roy, N., De Paepe, A., and Speleman, F. (2002). Accurate normalization of real-time quantitative RT-PCR data by geometric averaging of multiple internal control genes. *Genome Biology* 3(7)**,** research0034.0031. doi: 10.1186/gb-2002-3-7-research0034.

Whang, I., Kang, H.-S., and Kim, Y. (2020). Validation of Reference Genes for Quantitative Gene Expression Studies in *Octopus minor*. *Ocean Science Journal* 55(1)**,** 183-191. doi: 10.1007/s12601-020-0007-9.

Wong, W.-R., Brugman, K.I., Maher, S., Oh, J.Y., Howe, K., Kato, M., and Sternberg, P.W. (2019). Autism-associated missense genetic variants impact locomotion and neurodevelopment in *Caenorhabditis elegans*. *Human Molecular Genetics* 28(13)**,** 2271-2281. doi: 10.1093/hmg/ddz051.

Xie, F., Xiao, P., Chen, D., Xu, L., and Zhang, B. (2012). miRDeepFinder: a miRNA analysis tool for deep sequencing of plant small RNAs. *Plant Molecular Biology* 80(1)**,** 75-84. doi: 10.1007/s11103-012-9885-2.

Xu, R., and Zheng, X. (2018). Selection of reference genes for quantitative real-time PCR in *Octopus minor* (Cephalopoda: Octopoda) under acute ammonia stress. *Environmental Toxicology and Pharmacology* 60**,** 76-81. doi: https://doi.org/10.1016/j.etap.2018.04.010.

Yoshida, M.a., Hirota, K., Imoto, J., Okuno, M., Tanaka, H., Kajitani, R., Toyoda, A., Itoh, T., Ikeo, K., Sasaki, T., and Setiamarga, D.H.E. (2022). Gene recruitments and dismissals in the argonaut genome provide insights into pelagic lifestyle adaptation and shell-like eggcase reacquisition. *Genome Biology and Evolution*. doi: 10.1093/gbe/evac140.

Zarrella, I., Herten, K., Maes, G.E., Tai, S., Yang, M., Seuntjens, E., Ritschard, E.A., Zach, M., Styfhals, R., Sanges, R., Simakov, O., Ponte, G., and Fiorito, G. (2019). The survey and reference assisted assembly of the *Octopus vulgaris* genome. *Scientific Data* 6(1)**,** 13. doi: 10.1038/s41597-019-0017-6.

Zhang, J., Liu, C., He, M., Xiang, Z., Yin, Y., Liu, S., and Zhuang, Z. (2019). A full-length transcriptome of *Sepia esculenta* using a combination of single-molecule long-read (SMRT) and Illumina sequencing. *Marine Genomics* 43**,** 54-57. doi: https://doi.org/10.1016/j.margen.2018.08.008.

Zhang, X., Mao, Y., Huang, Z., Qu, M., Chen, J., Ding, S., Hong, J., and Sun, T. (2012). Transcriptome analysis of the *Octopus vulgaris* central nervous system. *PLoS ONE* 7(6)**,** e40320.

Zhang, Y., Mao, F., Mu, H., Huang, M., Bao, Y., Wang, L., Wong, N.-K., Xiao, S., Dai, H., Xiang, Z., Ma, M., Xiong, Y., Zhang, Z., Zhang, L., Song, X., Wang, F., Mu, X., Li, J., Ma, H., Zhang, Y., Zheng, H., Simakov, O., and Yu, Z. (2021). The genome of *Nautilus pompilius* illuminates eye evolution and biomineralization. *Nature Ecology & Evolution* 5(7)**,** 927-938. doi: 10.1038/s41559-021-01448-6.
